# Synthesis and Antifungal Activity of Novel 3-Caren-5-One Oxime Esters

**DOI:** 10.3390/molecules22091538

**Published:** 2017-09-12

**Authors:** Min Huang, Wen-Gui Duan, Gui-Shan Lin, Kun Li, Qiong Hu

**Affiliations:** School of Chemistry and Chemical Engineering, Guangxi University, Nanning 530004, Guangxi, China; gaominhuang@mail.gxu.cn (M.H.); kl756004166@sina.com (K.L.); hq460175625@163.com (Q.H.)

**Keywords:** 3-carene, 3-caren-5-one, 3-caren-5-one oxime ester, *E*-*Z* stereoisomer, antifungal activity

## Abstract

A series of novel 3-caren-5-one oxime esters were designed and synthesized by multi-step reactions in an attempt to develop potent antifungal agents. Two *E*-*Z* stereoisomers of the intermediate 3-caren-5-one oxime were separated by column chromatography for the first time. The structures of all the intermediates and target compounds were confirmed by UV-Vis, FTIR, NMR, ESI-MS, and elemental analysis. The antifungal activity of the target compounds was preliminarily evaluated by the in vitro method against *Fusarium oxysporum* f. sp. *cucumerinum*, *Physalospora piricola*, *Alternaria solani*, *Cercospora arachidicola*, *Gibberella zeae,* *Rhizoeotnia solani*, *Bipolaris maydis*, and *Colleterichum orbicalare* at 50 µg/mL. The target compounds exhibited best antifungal activity against *P. piricola*, in which compounds (*Z*)-**4r** (R = *β*-pyridyl), (*Z*)-**4q** (R = *α*-thienyl), (*E*)-**4f′** (R = *p*-F Ph), (*Z*)-**4i** (R = *m*-Me Ph), (*Z*)-**4j** (R = *p*-Me Ph), and (*Z*)-**4p** (R = *α*-furyl) had inhibition rates of 97.1%, 87.4%, 87.4%, 85.0%, 81.9%, and 77.7%, respectively, showing better antifungal activity than that of the commercial fungicide chlorothanil. Also, compound (*Z*)-**4r** (R = *β*-pyridyl) displayed remarkable antifungal activity against all the tested fungi, with inhibition rates of 76.7%, 82.7%, 97.1%, 66.3%, 74.7%, 93.9%, 76.7% and 93.3%, respectively, showing better or comparable antifungal activity than that of the commercial fungicide chlorothanil. Besides, the *E-Z* isomers of the target oxime esters were found to show obvious differences in antifungal activity. These results provide an encouraging framework that could lead to the development of potent novel antifungal agents.

## 1. Introduction

3-Carene, a naturally occurring bicyclic monoterpene containing a carbon-carbon double bond and a gem-dimethylcyclopropane ring, is a constituent of many essential oils and turpentine oils [1,2]. It was reported that the isomeric mixture of 3-carene showed a broad spectrum of activities, such as antimicrobial [3,4,5,6], anticancer [6], semiochemical [7,8], antioxidant [6,9], and fumigant activities [10]. Because of its special structure and bioactive activities, screening of structurally modified 3-carene derivatives for their bioactivity has received considerable attention. For instance, stable, potent, and selective sphingosine-1-phosphate receptor 1 (S1P_1_) agonists were successively synthesized by using (+)-3-carene as starting material [11]. Ingenol and (+)-phorbol, two crucial natural products with various biological activities, especially anticancer, have been also synthesized from inexpensive (+)-3-carene [12,13,14,15]. On the other hand, oxime esters as an important class of bioactive compounds for agrochemical and pharmaceutical use were investigated extensively by chemists. It was reported that oxime esters exhibited insecticidal [16,17], herbicidal [18], antiviral [19], antitumor [20,21,22], and antibacterial activities [23,24,25]. For example, a series of 3-ethoxy-4-hydroxybenzaldehyde oxime esters were synthesized and evaluated for their in vitro antifungal activity against three pathogenic fungi and antibacterial activity against three bacterial strains, and the structure-activity relationship was also preliminarily summarized [26]. In continuation of our interest in the bioactive properties of natural product-based compounds [27,28,29,30,31,32,33], a series of novel 3-caren-5-one oxime esters were designed and synthesized by integrating bioactive oxime esters into the skeleton of 3-carene. Structural characterization and antifungal evaluation of all the title compounds were carried out as well. In addition, Cu(II)HY zeolite was first employed as catalyst in the oxidative preparation of 3-caren-5-one, and the *E*-*Z* stereo-isomers of 3-caren-5-one oxime were also separated for the first time by column chromatography.

## 2. Results and Discussion

### 2.1. Synthesis and Characterization

**Scheme 1:**
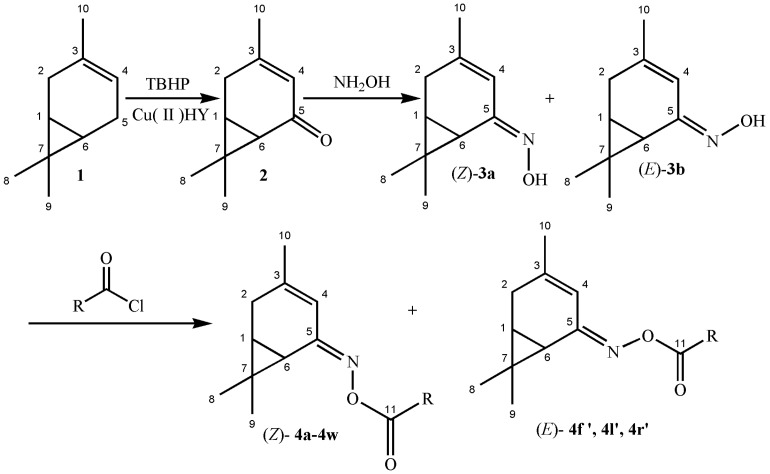
Synthesis of 3-caren-5-one oxime esters (*Z*)-**4a**–**4w**, and (*E*)-**4f′**, **4l′**, **4r′**. **4a**: R = Ph; **4b**: R = *o*-Cl Ph; **4c**: R = *m*-Cl Ph; **4d**: R = *p*-Cl Ph; **4e**: R = *o*-F Ph **4f**: R = *p*-F Ph; **4g**: R = *o*-OMe Ph; **4h**: R = *o*-Me Ph; **4i**: R = *m*-Me Ph; **4j**: R = *p*-Me Ph; **4k**: R = 2,4-Cl Ph; **4l**: R = 2,3-Cl Ph; **4m**: R = *p*-CH_2_Cl Ph; **4n**: R = cyclopentyl; **4o**: R = cyclohexyl; **4p**: R = *α*-furyl; **4q**: R = *α*-thienyl; **4r**: R = *β*-pyridyl; **4s**: R = *α*-Cl-*β*-pyridyl; **4t**: R = n-pentyl; **4u**: R = ethyl; **4v**: R = n-propyl; **4w**: R = n-butyl; **4f′**: R = *p*-F Ph; **4l**′: R = 2,3-Cl; **4r′**: R = *β*-pyridyl.

As illustrated in the Scheme 1, 3-caren-5-one (**2**) was prepared by oxidation of 3-carene (**1,** isomeric mixture) using TBHP (*tert*-butyl hydroperoxide) as oxidant in a 30% yield (GC). In this process, Cu(II)HY, in which Cu(II) ions were incorporated into HY zeolite, was chosen as a heterogeneous catalyst to increase the selectivity for **2** due to the shape-selectivity of this mesoporous material [34] and the activation capacity of copper ion to saturated C–H [35,36,37]. After the reaction was completed, this catalyst could easily be separated from the reaction system by filtration, and could be recycled several times.

**Figure 1:**
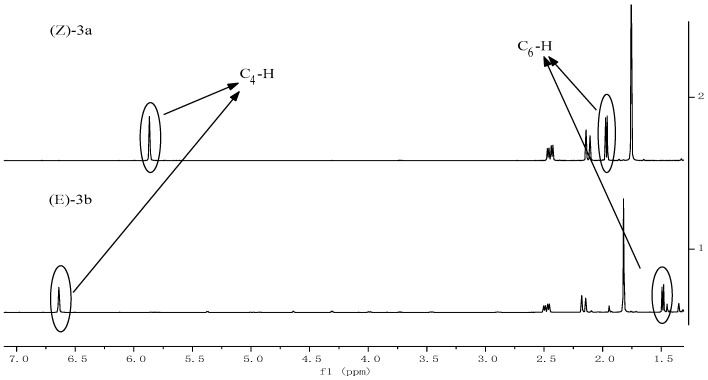
^1^H-NMR spectra of (*Z*)-**3a** and (*E*)-**3b**.

**Figure 2:**
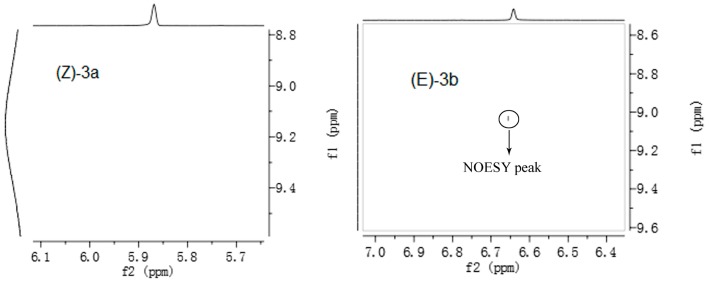
The coupling of C_4_-H and hydroxyl hydrogen in ^1^H-^1^H NOESY spectra.

3-Caren-5-one oxime (**3**, isomeric mixture) was prepared by oximation of compound **2** with NH_2_OH·HCl in a mixed solvent (EtOH:H_2_O = 5:1, *v*/*v*). Sodium acetate was added into the reaction system as an additive to neutralize HCl and form a buffer system. Two stereoisomers, (*Z*)-3-caren-5-one oxime ((*Z*)-**3a**) and (*E*)-3-caren-5-one oxime ((*E*)-**3b**), were isolated from compound **3** (**3a**:**3b** = 7:1, *w*/*w*, GC). In the ^1^H-NMR spectrum of (*Z*)-**3a**, the olefinic proton (C_4_-H) in the 3-carene skeleton showed signals at about 5.88 and the signals of the C_6_-H appeared at 1.99 ppm. However, the corresponding signals of another isomer (*E*)-**3b** were shifted to 6.65 ppm and 1.50 ppm, respectively (Figure 1). Due to the hydrogen bond interaction of the hydroxyl group oxygen with the olefinic proton (C_4_-H) or the C_6_-H, the signal of olefinic proton (C_4_-H) in the (*E*)-isomer and the signal of the C_6_-H in the (*Z*)-isomer shifted to downfield [38]. The structures of the two stereo-isomers were further confirmed by NOESY spectra. In the NOESY spectrum of (*Z*)-**3a**, no peak related to the coupling of hydroxyl hydrogen with C_4_-H was observed, while a peak corresponding to this space interaction in (*E*)-**3b** was found (Figure 2).

The target oxime esters were characterized by UV-Vis, IR, ^1^H-NMR, ^13^C-NMR, ESI-MS, and elemental analysis, and all the related spectra can be found in the Supplementary Materials. It was found that the molar extinction coefficient of all target compounds ranged from 3.89 to 4.42 (log ε), implying that a conjugated system existed in these compounds. In the IR spectra, the weak absorption bands at about 3018 cm^−1^ were attributed to the stretching vibrations of the unsaturated C–H in the 3-carene moiety. The strong absorption bands at about 1738 cm^−1^ were assigned to the vibrations of C=O in the ester moiety. The moderate absorption peaks at about 1649 cm^−1^ were due to the vibrations of C=N. In the ^1^H-NMR spectra, the protons of benzene ring showed signals at 7.10–8.20 ppm. The olefinic protons of the 3-carene scaffold showed signals at about 6.10 ppm for the (*Z*)-isomers and the (*E*)-isomers showed signals at about 6.60 ppm. The protons bonded to the saturated carbons displayed signals in the range of 0.50–3.00 ppm. The ^13^C-NMR spectra of the target compounds showed peaks for the olefinic carbons of the 3-carene moiety at 117.92–119.05 ppm (C_4_) and 147.49–149.74 ppm (C_3_) for the (*Z*)-isomers, however, the corresponding signals for (*E*)-isomers showed at about 113.20 (C_4_) ppm and 153.03 ppm (C_3_). The other saturated carbons displayed signals in the region of 13.59–55.94 ppm. The other signal peaks were correlated to the carbons of C=O, C=N, and the benzene ring. The molecular weights of the intermediates and target oxime esters were confirmed by the ESI-MS.

### 2.2. Antifungal Activity

The antifungal activities of the target compounds (*Z*)-**4a**–**4w** and (*E*)-**4f′** were evaluated by in vitro method against fusarium wilt on cucumber (*Fusarium oxysporum* f. sp. *cucumerinum*), speckle on peanut (*Cercospora arachidicola*), apple ring rot (*Physalospora piricola*), tomato early blight (*Alternaria solani*), wheat scab (*Gibberella zeae*), rice sheath blight (*Rhizoeotnia solani*), corn southern leaf blight (*Bipolaris maydis*), and watermelon anthracnose (*Colleterichum orbicalare*) at 50 μg/mL. The results are listed in Table 1.

It was found that, at 50 μg/mL, all the target compounds presented obviously different antifungal activity against the eight tested fungi. Compared with the intermediates **2**, **3a** and **3b**, some of the target compounds showed enhanced activities after esterification. On the whole, most of the target compounds exhibited best antifungal activity against *P. piricola*, in which compounds (*Z*)-**4r** (R = *β*-pyridyl), (*Z*)-**4q** (R = *α*-thienyl), (*E*)-**4f′** (R = *p*-F Ph), (*Z*)-**4i** (R = *m*-Me Ph), (*Z*)-**4j** (R = *p*-Me Ph), and (*Z*)-**4p** (R = *α*-furyl) had inhibition rates of 97.1%, 87.4%, 87.4%, 85.0%, 81.9%, and 77.7%, respectively, showing better antifungal activity than that of the commercial fungicide chlorothanil with the inhibition rate of 75.0%. It was also found that compound (*Z*)-**4r** (R = *β*-pyridyl) displayed remarkable antifungal activity against all the tested fungi, with inhibition rates of 76.7%, 82.7%, 97.1%, 66.3%, 74.7%, 93.9%, 76.7% and 93.3%, respectively, showing better or comparable antifungal activity than that of the commercial fungicide chlorothanil. Therefore, compound (*Z*)-**4r** (R = *β*-pyridyl) is a lead compound worthy of further investigation. Furthermore, it was found that (*E*)-isomer **4f′** (R = *p*-F Ph) exhibited much better antifungal activity than the corresponding (*Z*)-isomer 4f (R = *p*-F Ph) against all the tested fungi. For instance, (*E*)-isomer **4f′** (64.5%) showed 4.3 times that of **4f** (*Z*)-isomer (15.0%) against *C. arachidicola*, and the two isomers displayed 76.1% and 0.0% inhibition rates, respectively, against *R. solani*. The obvious difference in antifungal activity between *E-Z* isomers is very meaningful and requires further studies in photoisomerization and drug resistance.

## 3. Experimental Section

### 3.1. General Information

The GC analysis was performed on an Agilent 6890 GC (Agilent Technologies Inc., Santa Clara, CA, USA) equipped with a HP-1 (30 m, 0.530 mm, 0.88 μm) column. IR spectra were recorded (KBr pellet method) on a Nicolet iS50 FT-IR spectrometer (Thermo Scientific Co., Ltd., Madison, WI, USA). NMR spectra (including ^1^H-NMR, ^13^C-NMR, NOESY) were recorded in CDCl_3_ solvent on a Bruker Avance III HD 600 MHz spectrometer (Bruker Co., Ltd., Zurich, Switzerland) and chemical shifts are expressed in ppm (δ) downfield relative to TMS as an internal standard. MS spectra were obtained by means of the electrospray ionization (ESI) method on TSQ Quantum Access MAX HPLC-MS instrument (Thermo Scientific Co., Ltd., Waltham, MA, USA). Elemental analyses were measured using a PE 2400 II elemental analyzer (Perkin-Elmer Instruments Co., Ltd., USA). The UV spectra were measured on a Shimadzu UV-1800 spectrophotometer (Shimadzu Corp., Kyoto, Japan). Melting points were determined on a MP420 automatic melting point apparatus (Hanon Instruments Co., Ltd., Jinan, China) and were not corrected. Ultrasonic irradiation was carried out on XO-SM50 ultrasonic microwave reaction system (Nanjing Xianou Instrument Manufacturing Co., Ltd., Nanjing, China). 3-Carene (GC purity 98%, isomeric mixture) was provided by Wuzhou Pine Chemicals Co., Ltd. (Wuzhou, Guangxi, China). The 7Å HY zeolite was purchased from Nankai University Catalyst Co., Ltd. (Tianjin, China). Other reagents were purchased from commercial suppliers and used as received.

### 3.2. Preparation of Catalyst

Cu(II)HY zeolite catalyst used for the synthesis of 3-caren-5-one was prepared by the incipient wetness method [39]. HY zeolite with pore diameter of 7 Å was dried at 120 °C 3 h under air atmosphere. Then, a solution of Cu(NO_3_)_2_·3H_2_O (5.7 g) in deionized water based on the saturated water absorption amount of the zeolite (10.0 g) was added dropwise to the dried zeolite, with constant stirring and grinding. The resulting mixture was aged for 24 h, and then dried in vacuum at 120 °C for 2 h. The powder sample was calcinated at 550 °C for 4 h under air atmosphere.

### 3.3. Synthesis of 3-Caren-5-One (***2***)

3-Carene (10 mL, 63 mmol), Cu(II)HY zeolite catalyst (2.7 g) and CH_3_CN (20 mL) were mixed and then irradiated under ultrasonic wave for 40 min. When the mixture was heated to 45 °C, a mixed solution of 40 mL *tert*-butyl hydroperoxide (70% in water, wt %) (288 mmol) and 20 mL CH_3_CN was added to the reaction system by the way of constant flowing at a rate of 1 mL/min. Afterwards, the reaction mixture was stirred for 18 h at 45 °C. When the reaction was completed, the reaction mixture was cooled to room temperature and appropriate amount of Na_2_S_2_O_3_ was added into it to destroy the unreacted *tert*-butyl hydroperoxide. The solvent CH_3_CN was removed by vacuum distillation, and the residue was then extracted three times with EtOAc. The combined organic phase was washed with saturated sodium chloride and deionized water, respectively, and dried over with anhydrous Na_2_SO_4_. After the EtOAc was removed in vacuum, the crude product was purified by silica gel column chromatography (EtOAc−petroleum ether = 1:30, *v*/*v*) to obtain pale yellow liquid compound **2**. Yield 20%. UV-Vis (EtOH) λ_max_ (log ε): 226.50 (4.4.16) nm; IR (thin film, cm^−1^): 3029 (w, =C-H), 1655 (s, C=O); ^1^H-NMR δ = 5.83 (s, 1H, C_4_-H), 2.64 (dd, *J* = 20.4, 8.5 Hz, 1H, C_2_-H_a_), 2.33 (d, *J* = 20.8 Hz, 1H, C_2_-H_b_), 1.87 (s, 3H, C_3_-CH_3_), 1.56 (d, *J* = 7.8 Hz, 1H, C_6_-H), 1.45 (t, *J* = 8.0 Hz, 1H, C_1_-H), 1.19 (s, 3H, C_7_-CH_3_), 1.04 (s, 3H, C_7_-CH_3_); ^13^C-NMR δ = 196.67 (C_5_), 158.96 (C_3_), 126.40 (C_4_), 32.85 (C_6_), 28.43 (C_9_), 27.87 (C_2_), 25.86 (C_1_), 23.68 (C_10_), 22.57 (C_7_), 14.38 (C_8_); ESI-MS *m*/*z*: 151.17 [M + H]^+^. Anal. Calcd. For C_10_H_14_O (150.22): C, 79.96; H, 9.39. Found: C, 79.85; H, 9.36.

### 3.4. Synthesis of 3-Caren-5-One Oxime (***3***)

3-Caren-5-one (1.5 g, 10 mmol), NH_2_OH·HCl (1.04 g, 15 mmol), and NaOAc (4.08 g, 30 mmol) were dissolved in a mixed solvent (EtOH:H_2_O = 5:1, *v*/*v*). The reaction mixture was stirred at 80 °C for 3 h. The reaction process was monitored by TLC. When 3-carene-5-one was fully reacted, the mixture was cooled to room temperature. After the solvent EtOH was removed by rotary evaporation, the residue was extracted by EtOAc. The separated organic phase was washed with saturated NaCl solution three times, and concentrated under reduced pressure to obtain the crude product, which was purified by silica gel column chromatography using a mixed solvent (EtOAc−petroleum ether = 1:60, *v*/*v*) as eluent.

*(Z)-3-Caren-5-one oxime* ((*Z*)-**3a**) was obtained as a white needle crystal. Yield 63.5%, m.p.: 96.7–98.1 °C. UV-Vis (EtOH) λ_max_ (log ε): 239.70 (4.28) nm; IR (KBr, cm^−1^): 3464–3078 (s, br, O-H), 3033 (w, =C-H), 1659 (m, C=N), 1614 (m); ^1^H-NMR δ = 9.03 (s, 1H, OH), δ 5.87 (s, 1H, C_4_-H), 2.45 (dd, *J* = 20.0, 8.2 Hz, 1H, C_2_-H_a_), 2.12 (d, *J* = 20.0 Hz, 1H, C_2_-H_b_), 1.97 (d, *J* = 8.3 Hz, 1H, C_6_-H), 1.76 (s, 3H, C_3_-CH_3_), 1.23 (s, 3H, C_7_-CH_3_), 1.19 (t, *J* = 8.3 Hz, 1H, C_1_-H), 0.89 (s, 3H, C_7_-CH_3_); ^13^C-NMR δ = 155.00 (C_5_), 142.27 (C_3_), 119.32 (C_4_), 27.97 (C_6_), 27.08 (C_9_), 23.39 (C_2_), 22.37 (C_1_), 20.71 (C_10_), 19.67 (C_7_), 14.59 (C_8_); ESI-MS *m*/*z*: 166.14 [M + H]^+^. Anal. Calcd. For C_10_H_15_NO (165.23): C, 72.69; H, 9.15; N, 8.48. Found: C, 72.71; H, 9.17; N, 8.49.

*(E)-3-Caren-5-one oxime* ((*E*)-**3b**) was obtained as a white plate-like crystal. Yield 3%, m.p.: 73.5–75.0 °C. UV-Vis (EtOH) λ_max_ (log ε): 235.80 (3.89) nm; IR (KBr, cm^−1^): 3432–3081 (s, br, O-H), 3015 (w, =C-H), 1644 (m, C=N), 1608 (m); ^1^H-NMR δ = 8.98 (s, 1H, OH), 6.64 (s, 1H, C_4_-H), 2.48 (dd, *J* = 20.4, 8.0 Hz, 1H, C_2_-H_a_), 2.16 (d, *J* = 20.5 Hz, 1H, C_2_-H_b_), 1.82 (s, 3H, C_3_-CH_3_), 1.49 (d, *J* = 8.7 Hz, 1H, C_6_-H), 1.19 (t, *J* = 8.3 Hz, 1H, C_1_-H), 1.13 (s, 3H, C_7_-CH_3_), 0.88 (s, 3H, C_7_-CH_3_); ^13^C-NMR δ = 152.05 (C_5_), 147.32 (C_3_), 112.59 (C_4_), 28.03 (C_6_), 27.71 (C_9_), 23.86 (C_2_), 23.54 (C_1_), 21.82 (C_10_), 19.61 (C_7_), 14.15 (C_8_); ESI-MS *m*/*z*: 166.14 [M + H]^+^. Anal. Calcd. For C_10_H_15_NO (165.23): C, 72.69; H, 9.15; N, 8.48. Found: C, 72.60; H, 9.18; N, 8.46.

### 3.5. General Procedure for Synthesis of 3-Caren-5-One Oxime Esters (Z)-***4a***–***4w***, (E)-***4f*′**, ***4l*′**, ***4r*′**

A solution of acyl chloride (1.2 mmol) in CH_2_Cl_2_ (3 mL) was added slowly to a solution of (*Z*)- or (*E*)-3-carene-5-one oxime (1 mmol) and triethylamine in CH_2_Cl_2_ (5 mL) with ice-water cooling. The reaction process was monitored by TLC. Upon completion, 5 mL deionized water was added to the reaction mixture to destroy the unreacted acyl chloride. Then, the organic layer was separated, washed with deionized water three times, and concentrated in vacuum. The crude product was further purified by silica gel chromatography to afford the target compounds (*Z*)-**4a**–**4w**, and (*E*)-**4f′**, **4l′**, **4r′**. Only three (*E*)-oxime esters, i.e., (*E*)-**4f′**, **4l′**, **4r′**, were reported in this study because of the very low yield of (*E*)-**3b**.

*(Z)-3-Caren-5-one O-(benzoyl) oxime* ((*Z*)-**4a**). Pale yellow solid. Yield: 65.4%, m.p.: 97.4–98.7 °C. UV-Vis (EtOH) λ_max_ (log ε): 257.40 (4.34) nm, 236.70 (4.25) nm; IR (KBr, cm^−1^): 3069 (w), 3015 (w, Ar-H, =C-H), 1739 (s, C=O), 1658 (m, C=N), 1602 (w), 1581 (s), 1495 (w), 1456 (m, Ar); ^1^H-NMR δ = 8.10 (d, *J* = 7.1 Hz, 2H, C_13_-H, C_17_-H), 7.59 (t, *J* = 7.4 Hz, 1H, C_15_-H), 7.47 (t, *J* = 7.8 Hz, 2H, C_14_-H, C_16_-H), 6.17 (s, 1H, C_4_-H), 2.56 (dd, *J* = 20.2, 8.4 Hz, 1H, C_2_-H_a_), 2.21 (d, *J* = 19.5 Hz, 1H, C_2_-H_b_), 2.05 (d, *J* = 8.2 Hz, 1H, C_6_-H), 1.85 (s, 3H, C_3_-CH_3_), 1.34 (d, *J* = 7.8 Hz, 1H, C_1_-H), 1.30 (s, 3H, C_7_-CH_3_), 0.94 (s, 3H, C_7_-CH_3_); ^13^C-NMR δ = 164.12 (C_11_), 161.89 (C_5_), 147.97 (C_3_), 133.02 (C_15_), 129.60 (C_12_), 129.53 (C_13_ and C_17_), 128.49 (C_14_ and C_16_), 118.38 (C_4_), 28.09 (C_6_), 27.15 (C_9_), 23.77 (C_2_), 23.08 (C_1_), 21.68 (C_10_), 21.32 (C_7_), 14.59 (C_8_); ESI-MS *m*/*z*: 270.09 [M + H]^+^. Anal. Calcd. For C_17_H_19_NO_2_ (269.34): C, 75.81; H, 7.11; N, 5.20. Found: C, 75.79; H, 7.12; N, 5.21.

*(Z)-3-Caren-5-one O-(2-chlorobenzoyl) oxime* ((*Z*)-**4b**). White solid. Yield: 74.0%, m.p.: 116.3–117.8 °C. UV-Vis (EtOH) λ_max_ (log ε): 253.50 (4.23) nm. IR (KBr, cm^−1^): 3048 (w), 3012 (w, Ar-H, =C-H), 1726 (s, C=O), 1652 (m, C=N), 1581 (m), 1464 (w, Ar); ^1^H-NMR δ = 7.88 (dd, *J* = 7.8, 1.6 Hz, 1H, C_17_-H), 7.47 (dd, *J* = 8.0, 1.4 Hz, 1H, C_14_-H), 7.44 (td, *J* = 8.1, 7.6, 1.6 Hz, 1H, C_15_-H), 7.35 (td, *J* = 7.6, 1.4 Hz, 1H, C_16_-H), 6.14 (s, 1H, C_4_-H), 2.54 (dd, *J* = 20.3, 8.3 Hz, 1H, C_2_-H_a_), 2.21 (d, *J* = 20.3 Hz, 1H, C_2_-H_b_), 2.08 (d, *J* = 8.1 Hz, 1H, C_6_-H), 1.85 (s, 3H, C_3_-CH_3_), 1.29 (t, *J* = 8.2 Hz, 1H, C_1_-H), 1.22 (s, 3H, C_7_-CH_3_), 0.94 (s, 3H, C_7_-CH_3_); ^13^C-NMR δ = 164.06 (C_11_), 162.31 (C_5_), 148.35 (C_3_), 133.30 (C_12_), 132.55 (C_15_), 131.70 (C_13_), 130.91 (C_17_), 130.03 (C_14_), 126.67 (C_16_), 118.22 (C_4_), 28.08 (C_6_), 27.21 (C_9_), 23.77 (C_2_), 23.13 (C_1_), 21.98 (C_10_), 21.43 (C_7_), 14.56 (C_8_); ESI-MS *m*/*z*: 304.07 [M + H]^+^. Anal. Calcd. For C_17_H_18_ClNO_2_ (303.78): C, 67.21; H, 5.97; N, 4.61. Found: C, 67.25; H, 5.93; N, 4.62.

*(Z)-3-Caren-5-one O-(3-chlorobenzoyl) oxime* ((*Z*)-**4c**). White solid. Yield: 80.3%, m.p.: 137.3–138.1 °C. UV-Vis (EtOH) λ_max_ (log ε): 259.90 (4.08) nm, 229.80 (4.07) nm; IR (KBr, cm^−1^): 3084 (w), 3024 (w, Ar-H, =C-H), 1741 (s, C=O), 1652 (m, C=N), 1581 (m), 1491 (w, Ar);^1^H-NMR δ = 8.07 (s, 1H, C_13_-H), 7.98 (d, *J* = 7.8 Hz, 1H, C_17_-H), 7.56 (d, *J* = 8.0 Hz, 1H, C_15_-H), 7.42 (t, *J* = 7.9 Hz, 1H, C_16_-H), 6.16 (s, 1H, C_4_-H), 2.57 (dd, *J* = 20.3, 8.4 Hz, 1H, C_2_-H_a_), 2.22 (d, *J* = 20.3 Hz, 1H, C_2_-H_b_), 2.02 (d, *J* = 8.2 Hz, 1H, C_6_-H), 1.86 (s, 3H, C_3_-CH_3_), 1.35 (t, *J* = 8.4 Hz, 1H, C_1_-H), 1.32 (s, 3H, C_7_-CH_3_), 0.94 (s, 3H, C_7_-CH_3_). ^13^C-NMR δ = 162.94 (C_11_), 162.32 (C_5_), 148.54 (C_3_), 134.66 (C_12_), 133.07 (C_15_), 131.31 (C_14_), 129.85 (C_16_), 129.65 (C_13_), 127.62 (C_17_), 118.12 (C_4_), 28.03 (C_6_), 27.16 (C_9_), 23.80 (C_2_), 23.11 (C_1_), 21.80 (C_10_), 21.35 (C_7_), 14.59 (C_8_); ESI-MS *m*/*z*: 304.05 [M + H]^+^. Anal. Calcd. For C_17_H_18_ClNO_2_ (303.78): C, 67.21; H, 5.97; N, 4.61. Found: C, 67.23; H, 5.98; N, 4.60.

*(Z)-3-Caren-5-one O-(4-chlorobenzoyl) oxime* ((*Z*)-**4d**). White solid. Yield: 78.6%, m.p.: 149.9–151.3 °C. UV-Vis (EtOH) λ_max_ (log ε): 258.80 (4.32) nm, 245.90 (4.32) nm; IR (KBr, cm^−1^): 3066 (w), 3024 (w, Ar-H, =C-H), 1736 (s, C=O), 1659 (m, C=N), 1596 (m), 1578 (m), 1486 (m, Ar); ^1^H-NMR δ = 8.03 (d, *J* = 8.4 Hz, 2H, C_13_-H, C_17_-H), 7.45 (d, *J* = 8.4 Hz, 2H, C_14_-H, C_16_-H), 6.16 (s, 1H, C_4_-H), 2.56 (dd, *J* = 20.2, 8.3 Hz, 1H, C_2_-H_a_), 2.22 (d, *J* = 20.3 Hz, 1H, C_2_-H_b_), 2.01 (d, *J* = 8.1 Hz, 1H, C_6_-H), 1.85 (s, 3H, C_3_-CH_3_), 1.34 (t, *J* = 8.2 Hz, 1H, C_1_-H), 1.29 (s, 3H, C_7_-CH_3_), 0.94 (s, 3H, C_7_-CH_3_); ^13^C-NMR δ = 163.34 (C_11_), 162.15 (C_5_), 148.32 (C_3_), 139.52 (C_15_), 130.88 (C_12_), 128.89 (C_13_, C_14_), 128.02 (C_15_), 118.22 (C_3_), 28.10 (C_6_), 27.15 (C_9_), 23.79 (C_2_), 23.13 (C_1_), 21.77 (C_10_), 21.32 (C_7_), 14.58 (C_8_); ESI-MS *m*/*z*: 304.04 [M + H]^+^. Anal. Calcd. For C_17_H_18_ClNO_2_ (303.78): C, 67.21; H, 5.97; N, 4.61. Found: C, 67.23; H, 5.95; N, 4.63.

*(Z)-3-Caren-5-one O-(2-fluorobenzoyl) oxime* ((*Z*)-**4e**). White solid. Yield: 76.9%, m.p.: 128.7–131.2 °C. UV-Vis (EtOH) λ_max_ (log ε): 257.60 (4.29) nm, 224.70 (4.29) nm; IR (KBr, cm^−1^): 3057 (w), 3009 (w, Ar-H, =C-H), 1736 (s, C=O), 1650 (m, C=N), 1608 (m), 1575 (m), 1486 (m), 1453 (m, Ar); ^1^H-NMR δ = 8.07 (td, *J* = 7.5, 1.8 Hz, 1H, C_17_-H), 7.57–7.52 (m, 1H, C_15_-H), 7.25 (td, *J* = 7.7, 1.1 Hz, 1H, C_16_-H), 7.16 (ddd, *J* = 10.7, 8.3, 0.9 Hz, 1H, C_14_-H), 6.15 (s, 1H, C_4_-H), 2.55 (dd, *J* = 20.3, 8.4 Hz, 1H, C_2_-H_a_), 2.21 (d, *J* = 20.3 Hz, 1H, C_2_-H_b_), 2.12 (d, *J* = 8.1 Hz, 1H, C_6_-H), 1.85 (s, 3H, C_3_-CH_3_), 1.31 (t, *J* = 7.9 Hz, 1H, C_1_-H), 1.26 (s, 3H, C_7_-CH_3_), 0.93 (s, 3H, C_7_-CH_3_). ^13^C-NMR δ = 162.29 (C_11_), 160.83 (C_5_), 148.32 (C_3_), 134.61 (C_15_), 134.55 (C_17_), 132.63 (C_16_), 124.13 (C_12_), 118.23 (C_4_), 117.02 (C_13_), 116.87 (C_14_), 27.90 (C_6_), 27.24 (C_9_), 23.78 (C_2_), 23.13 (C_1_), 21.88 (C_10_), 21.45 (C_7_), 14.56 (C_8_); ESI-MS *m*/*z*: 288.08 [M + H]^+^. Anal. Calcd. For C_17_H_18_FNO_2_ (287.33): C, 71.06; H, 6.31; N, 4.87. Found: C, 71.10; H, 6.30; N, 4.85.

*(Z)-3-Caren-5-one O-(4-fluorobenzoyl) oxime* ((*Z*)-**4f**). White solid. Yield: 65.2%, m.p.: 133.2–135.0 °C. UV-Vis (EtOH) λ_max_ (log ε): 257.00 (4.29) nm, 229.10 (4.26) nm; IR (KBr, cm^−1^): 3041 (w), 3018 (w, Ar-H, =C-H), 1748 (s, C=O), 1659 (m, C=N), 1602 (m), 1581 (m), 1507 (m, Ar); ^1^H-NMR δ: 8.11 (dd, *J* = 8.9, 5.4 Hz, 2H, C_13_-H, C_17_-H), 7.15 (t, *J* = 8.7 Hz, 2H, C_14_-H, C_16_-H), 6.16 (s, 1H, C_4_-H), 2.56 (dd, *J* = 20.3, 8.4 Hz, 1H, C_2_-H_a_), 2.22 (d, *J* = 20.3 Hz, 1H, C_2_-H_b_), 2.02 (d, *J* = 8.2 Hz, 1H, C_6_-H), 1.85 (s, 3H, C_3_-CH_3_), 1.34 (t, *J* = 8.2 Hz, 1H, C_1_-H), 1.30 (s, 3H, C_7_-CH_3_), 0.94 (s, 3H, C_7_-CH_3_); ^13^C-NMR δ: 166.63 (C_11_), 164.95 (C_5_), 163.23 (C_15_), 162.01 (C_12_), 148.19 (C_3_), 132.07 (C_13_ or C_17_), 132.01 (C_13_ or C_17_), 118.27 (C_4_), 115.79 (C_14_ or C_16_), 115.64 (C_14_ or C_16_), 28.11 (C_6_), 27.15 (C_9_), 23.78 (C_2_), 23.12 (C_1_), 21.73 (C_10_), 21.32 (C_7_), 14.59 (C_8_); ESI-MS *m*/*z*: 288.07 [M + H]^+^. Anal. Calcd. For C_17_H_18_FNO_2_ (287.33): C, 71.06; H, 6.31; N, 4.87. Found: C, 71.03; H, 6.29; N, 4.86.

*(Z)-3-Caren-5-one O-(2-methoxybenzoyl) oxime* ((*Z*)-**4g**). White solid. Yield: 65.0%, m.p.: 123.9–125.5 °C. UV-Vis (EtOH) λ_max_ (log ε): 254.50 (4.38) nm; IR (KBr): 3054 (w), 3006 (w, Ar-H, =C-H), 1724 (s, C=O), 1656 (m, C=N), 1599 (m), 1581 (m), 1489 (m), 1462 (m, Ar); ^1^H-NMR δ = 7.85 (dd, *J* = 7.7, 1.8 Hz, 1H, C_17_-H), 7.48 (td, *J* = 9.0, 8.2, 1.8 Hz, 1H, C_15_-H), 7.04–6.99 (m, 1H, C_16_-H), 7.01–6.97 (m, 1H, C_14_-H), 6.15 (s, 1H, C_4_-H), 3.89 (s, 3H, O-CH_3_), 2.53 (dd, *J* = 20.2, 8.3 Hz, 1H, C_2_-H_a_), 2.20 (d, *J* = 20.2 Hz, 1H, C_2_-H_b_), 2.08 (d, *J* = 8.1 Hz, 1H, C_6_-H), 1.84 (s, 3H, C_3_-CH_3_), 1.29 (t, *J* = 8.1 Hz, 1H, C_6_-H), 1.23 (s, 3H, C_7_-CH_3_), 0.93 (s, 3H, C_7_-CH_3_); ^13^C-NMR δ = 164.58 (C_11_), 161.72 (C_5_), 158.98 (C_13_), 147.53 (C_3_), 133.45 (C_15_), 131.79 (C_17_), 120.20 (C_12_), 119.68 (C_16_), 118.58 (C_4_), 111.97 (C_14_), 55.94 (C_18_), 28.17 (C_6_), 27.21 (C_9_), 23.73 (C_2_), 23.21 (C_1_), 21.70 (C_10_), 21.30 (C_7_), 14.56 (C_8_); ESI-MS *m*/*z*: 300.01 [M + H]^+^. Anal. Calcd. For C_17_H_21_NO_2_ (299.36): C, 72.22; H, 7.07; N, 4.68. Found: C, 72.17; H, 7.04; N, 4.71.

*(Z)-3-Caren-5-one O-(2-methylbenzoyl) oxime* ((*Z*)-**4h**). White solid. Yield: 65%, m.p.: 64.7–67.5 °C. UV-Vis (EtOH) λ_max_ (log ε): 255.80 (4.42) nm; IR (KBr, cm^−1^): 3048 (w), 3015 (w, Ar-H, =C-H), 1748 (s, C=O), 1653 (m, C=N), 1605 (w), 1581 (m), 1486 (w), 1453 (m, Ar); ^1^H-NMR δ = 7.92 (d, *J* = 6.8 Hz, 1H, C_17_-H), 7.42 (t, *J* = 7.5 Hz, 1H, C_15_-H), 7.28 (d, *J* = 7.6 Hz, 1H, C_16_-H), 7.26 (d, *J* = 7.5 Hz, 1H, C_14_-H), 6.16 (s, 1H, C_4_-H), 2.66 (s, 3H, C_13_-CH_3_), 2.54 (dd, *J* = 20.6, 8.7 Hz, 1H, C_2_-H_a_), 2.20 (d, *J* = 20.3 Hz, 1H, C_2_-H_b_), 2.00 (d, *J* = 8.2 Hz, 1H, C_6_-H), 1.85 (s, 3H, C_3_-CH_3_), 1.30 (t, *J* = 8.2 Hz, 1H, C_1_-H), 1.25 (s, 3H, C_7_-CH_3_), 0.94 (s, 3H, C_7_-CH_3_); ^13^C-NMR δ = 165.43 (C_11_), 161.56 (C_5_), 147.75 (C_3_), 140.06 (C_13_), 131.95 (C_15_), 131.67 (C_12_), 130.30 (C_17_), 129.17 (C_14_), 125.70 (C_16_), 118.49 (C_4_), 28.08 (C_6_), 27.13 (C_9_), 23.74 (C_2_), 23.02 (C_1_), 21.72 (C_10_ and C_18_), 21.41 (C_7_), 14.61 (C_8_); ESI-MS *m*/*z*: 284.12 [M + H]^+^. Anal. Calcd. For C_18_H_21_NO_2_ (283.36): C, 76.29; H, 7.47; N, 4.94. Found: C, 76.21; H, 7.50; N, 4.90.

*(Z)-3-Caren-5-one O-(3-methylbenzoyl) oxime* ((*Z*)-**4i**). White solid. Yield: 70.0%, m.p.: 131.8–133.2 °C. UV-Vis (EtOH) λ_max_ (log ε): 258.30 (4.31) nm; IR (KBr, cm^−1^): 3054 (w), 3021 (w, Ar-H, =C-H), 1736 (s, C=O), 1650 (m, C=N), 1572 (m), 1453 (m, Ar); ^1^H-NMR δ = 7.93 (s, 1H, C_13_-H), 7.89 (d, *J* = 7.6 Hz, 1H, C_17_-H), 7.39 (d, *J* = 7.6 Hz, 1H, C_15_-H), 7.35 (t, *J* = 7.6 Hz, 1H, C_16_-H), 6.16 (s, 1H, C_4_-H), 2.56 (dd, *J* = 19.9, 8.1 Hz, 1H, C_2_-H_a_), 2.41 (s, 3H, C_14_-CH_3_), 2.21 (d, *J* = 20.3 Hz, 1H, C_2_-H_b_), 2.05 (d, *J* = 8.2 Hz, 1H, C_6_-H), 1.85 (s, 3H, C_3_-CH_3_), 1.33 (t, *J* = 8.3 Hz, 1H, C_1_-H), 1.31 (s, 3H, C_7_-CH_3_), 0.94 (s, 3H, C_7_-CH_3_); ^13^C-NMR δ = 164.29 (C_11_), 161.83 (C_5_), 147.93 (C_3_), 138.29 (C_14_), 133.80 (C_15_), 130.22 (C_12_), 129.48 (C_13_), 128.36 (C_16_), 126.58 (C_17_), 118.39 (C_4_), 28.03 (C_6_), 27.15 (C_9_), 23.78 (C_2_), 23.02 (C_1_), 21.62 (C_18_), 21.35 (C_10_), 21.31 (C_7_), 14.60 (C_8_); ESI-MS *m*/*z*: 284.13 [M + H]^+^. Anal. Calcd. For C_18_H_21_NO_2_ (283.36): C, 76.29; H, 7.47; N, 4.94. Found: C, 76.25; H, 7.49; N, 4.92.

*(Z)-3-Caren-5-one O-(4-methylbenzoyl) oxime* ((*Z*)-**4j**). White solid. Yield: 75.3%, m.p.: 125.9–127.6 °C. UV-Vis (EtOH) λ_max_ (log ε): 259.30 (4.38) nm; IR (KBr, cm^−1^): 3066 (w), 3033 (w, Ar-H, =C-H), 1742 (s, C=O), 1656 (m, C=N), 1617 (m), 1572 (m), 1510 (m), 1450 (m, Ar); ^1^H-NMR δ = 7.99 (d, *J* = 8.2 Hz, 2H, C_13_-H, C_17_-H), 7.27 (d, *J* = 6.5 Hz, 2H, C_14_-H, C_16_-H), 6.16 (s, 1H, C_4_-H), 2.55 (dd, *J* = 20.2, 7.7 Hz, 1H, C_2_-H_a_), 2.42 (s, 3H, C_15_-CH_3_), 2.21 (d, *J* = 20.3 Hz, 1H, C_2_-H_b_), 2.04 (d, *J* = 8.2 Hz, 1H, C_6_-H), 1.84 (s, 3H, C_3_-CH_3_), 1.32 (t, *J* = 8.0 Hz, 1H, C_1_-H), 1.29 (s, 3H, C_7_-CH_3_), 0.94 (s, 3H, C_7_-CH_3_). ^13^C-NMR δ = 164.23 (C_11_), 161.72 (C_5_), 147.80 (C_3_), 143.75 (C_15_), 129.57 (C_12_), 129.21 (C_13_ and C_17_), 126.78 (C_14_ and C_16_), 118.44 (C_4_), 28.07 (C_6_), 27.15 (C_9_), 23.76 (C_2_), 23.05 (C_1_), 21.70 (C_18_), 21.63 (C_10_), 21.32 (C_7_), 14.59 (C_8_); ESI-MS *m*/*z*: 284.17 [M + H]^+^. Anal. Calcd. For C_18_H_21_NO_2_ (283.36): C, 76.29; H, 7.47; N, 4.94. Found: C, 76.32; H, 7.45; N, 4.93.

*(Z)-3-Caren-5-one O-(2,4-dichlorobenzoyl) oxime* ((*Z*)-**4k**). White solid. Yield: 65.0%, m.p.: 82.0–83.0 °C. UV-Vis (EtOH) λ_max_ (log ε): 249.50 (4.30) nm; IR (KBr, cm^−1^): 3087 (w), 3018 (w, Ar-H, =C-H), 1736 (s, C=O), 1653 (m, C=N), 1587 (m), 1545 (m), 1468 (m), 1450 (m, Ar); ^1^H-NMR δ = 7.85 (d, *J* = 8.4 Hz, 1H, C_17_-H), 7.50 (d, *J* = 2.0 Hz, 1H, C_14_-H), 7.34 (dd, *J* = 8.4, 2.0 Hz, 1H, C_16_), 6.13 (s, 1H, C_4_-H), 2.55 (dd, *J* = 20.6, 8.7 Hz, 1H, C_2_-H_a_), 2.21 (d, *J* = 20.3 Hz, 1H, C_2_-H_b_), 2.05 (d, *J* = 8.1 Hz, 1H, C_6_-H), 1.85 (s, 3H, C_3_-CH_3_), 1.30 (t, *J* = 8.0 Hz, 1H, C_1_-H), 1.22 (s, 3H, C_7_-CH_3_), 0.93 (s, 3H, C_7_-CH_3_). ^13^C-NMR δ = 163.20 (C_11_), 162.44 (C_5_), 148.59 (C_3_), 138.35 (C_15_), 134.38 (C_13_), 132.79 (C_12_), 130.86 (_17_), 128.34 (_14_), 127.16 (C_16_), 118.10 (C_4_), 28.10 (C_6_), 27.20 (C_9_), 23.78 (C_2_), 23.16 (C_1_), 22.04 (C_10_), 21.48 (C_7_), 14.56 (C_8_); ESI-MS *m*/*z*: 338.06 [M + H]^+^. Anal. Calcd. For C_17_H_17_Cl_2_NO_2_ (338.23): C, 60.37; H, 5.07; N, 4.14. Found: C, 60.30; H, 5.11; N, 4.17.

*(Z)-3-Caren-5-one O-(2,3-dichlorobenzoyl) oxime* ((*Z*)-**4l**). Yellow liquid. Yield: 60.7%. UV-Vis (EtOH) λ_max_ (log ε): 254.19 (4.16) nm, 224.42 (4.28) nm; IR (thin film, cm^−1^): 3076 (w), 3014 (w, Ar-H, =C-H), 1750 (s, C=O), 1653 (m, C=N), 1581 (m), 1560 (w), 1516 (w),1447 (m, Ar); ^1^H-NMR δ = 7.71 (dd, *J* = 7.8, 1.6 Hz, 1H, C_17_-H), 7.62 (dd, *J* = 8.0, 1.6 Hz, 1H, C_15_-H), 7.30 (t, *J* = 7.9 Hz, 1H, C_16_-H), 6.13 (s, 1H, C_4_-H), 2.55 (dd, *J* = 21.1, 8.3 Hz, 1H, C_2_-H_a_), 2.21 (d, *J* = 21.1 Hz, 1H, C_2_-H_b_), 2.02 (d, *J* = 8.1 Hz, 1H, C_6_-H), 1.85 (s, 3H, C_3_-CH_3_), 1.30 (t, *J* = 8.0 Hz, 1H, C_1_-H), 1.22 (s, 3H, C_7_-CH_3_), 0.94 (s, 3H, C_7_-CH_3_). ^13^C-NMR δ = 164.70 (C_11_), 163.64 (C_5_), 149.74 (C_3_), 135.48 (C_12_), 134.17 (C_15_), 133.69 (C_14_), 132.38 (C_13_), 130.37 (C_17_), 128.32 (C_16_), 119.05 (C_4_), 29.11 (C_6_), 28.21 (C_9_), 24.78 (C_2_), 24.20 (C_1_), 23.08 (_10_), 22.36 (C_7_), 15.56 (_8_); ESI-MS *m*/*z*: 337.99 [M − H]^-^. Anal. Calcd. For C_17_H_17_Cl_2_NO_2_ (338.23): C, 60.37; H, 5.07; N, 4.14. Found: C, 60.40; H, 5.09; N, 4.15.

*(Z)-3-Caren-5-one O-(4-chloromethylbenzoyl) oxime* ((*Z*)-**4m**). White solid. Yield: 78%, m.p.: 121.9–123.2 °C. UV-Vis (EtOH) λ_max_ (log ε): 258.00 (4.23) nm, 238.50 (4.31) nm; IR (KBr, cm^−1^): 3039 (w), 3009 (w, Ar-H, =C-H), 1748 (s, C=O), 1653 (m, C=N), 1611 (m), 1575 (m), 1516 (w), 1426 (m, Ar); ^1^H-NMR δ = 8.09 (d, *J* = 8.3 Hz, 2H, C_13_-H, C_17_-H), 7.50 (d, *J* = 8.2 Hz, 2H, C_14_-H, C_16_-H), 6.16 (s, 1H, C_4_-H), 4.63 (s, 2H, C_18_-H), 2.56 (dd, *J* = 20.3, 8.3 Hz, 1H, C_2_-H_a_), 2.22 (d, *J* = 20.3 Hz, 1H, C_2_-H_b_), 2.03 (d, *J* = 8.1 Hz, 1H, C_6_-H), 1.85 (s, 3H, C_3_-CH_3_), 1.34 (t, *J* = 8.2 Hz, 1H, C_1_-H), 1.30 (s, 3H, C_7_-CH_3_), 0.94 (s, 3H, C_7_-CH_3_); ^13^C-NMR δ = 163.66 (C_11_), 162.08 (C_5_), 148.22 (C_3_), 142.39 (C_15_), 130.63 (C_12_), 129.97 (C_13_, C_17_), 128.62 (C_14_, C_16_), 118.27 (C_4_), 45.36 (C_18_), 28.10 (C_6_), 27.16 (C_9_), 23.78 (C_2_), 23.12 (C_1_), 21.75 (C_10_), 21.34 (C_7_), 14.59 (C_8_); ESI-MS *m*/*z*: 318.10 [M + H]^+^. Anal. Calcd. For C_18_H_20_ClNO_2_ (317.81): C, 68.03; H, 6.34; N, 4.41. Found: C, 68.00; H, 6.35; N, 4.43.

*(Z)-3-Caren-5-one O-cyclopentylcarbonyl oxime* ((*Z*)-**4n**). White solid. Yield: 64.5%, m.p.: 58.0–60.2 °C. UV-Vis (EtOH) λ_max_ (log ε): 241.40 (4.19) nm; IR (KBr, cm^−1^): 3030 (w, =C-H), 1757 (s, C=O), 1653 (m, C=N); ^1^H-NMR δ = 6.07 (s, 1H, C_4_-H), 2.89 (p, *J* = 8.0 Hz, 1H, C_12_-H), 2.51 (dd, *J* = 20.3, 8.4 Hz, 1H, C_2_-H_a_), 2.17 (d, *J* = 20.3 Hz, 1H, C_2_-H_b_), 1.94 (tt, *J* = 12.5, 6.8 Hz, 4H, C_13_-H_2_, C_16_-H_2_), 1.89 (d, *J* = 8.2 Hz, 1H, C_6_-H), 1.81 (s, 3H, C_3_-CH_3_), 1.78–1.72 (m, 2H, C_14_-2H), 1.62 (dt, *J* = 8.9, 4.2 Hz, 2H, C_15_-H ), 1.27 (t, *J* = 8.3 Hz, 1H, C_1_-H), 1.23 (s, 3H, C_7_-CH_3_), 0.89 (s, 3H, C_7_-CH_3_). ^13^C-NMR δ = 174.14 (C_11_), 161.27 (C_5_), 147.49 (C_3_), 118.48 (C_4_), 42.99 (C_12_), 30.08 (C_13_), 30.02 (C_16_), 28.02 (C_6_), 27.12 (C_9_), 25.86 (C_14_ and C_15_), 23.71 (C_2_), 22.97 (C_1_), 21.50 (C_10_), 21.04 (C_7_), 14.56 (C_8_); ESI-MS *m*/*z*: 262.13 [M + H]^+^. Anal. Calcd. For C_16_H_23_NO_2_ (261.36): C, 73.53; H, 8.87; N, 5.36. Found: C, 73.51; H, 8.89; N, 5.37.

*(Z)-3-Caren-5-one O-cyclohexylcarbonyl oxime* ((*Z*)-**4o**). White solid. Yield: 64.8%, m.p.: 81.4–82.5 °C. UV-Vis (EtOH) λ_max_ (log ε): 243.60 (4.29) nm: IR (KBr, cm^−1^): 3027 (w, =C-H), 1753 (s, C=O), 1650 (m, C=N); ^1^H-NMR δ = 6.07 (s, 1H, C_4_-H), 2.54–2.44 (m, 2H, C_2_-H_a_, C_12_-H), 2.17 (d, *J* = 20.3 Hz, 1H, C_2_-H_b_), 1.98 (s, 2H, C_13_-H), 1.89 (d, *J* = 8.2 Hz, 1H, C_6_-H), 1.81 (s, 3H, C_3_-CH_3_), 1.78 (dd, *J* = 9.1, 3.9 Hz, 2H, C_17_-H), 1.57 (q, *J* = 11.7 Hz, 2H, C_15_-H), 1.37–1.25 (m, 5H, C_1_-H, C_14_-H, C_16_-H), 1.23 (s, 3H, C_7_-CH_3_), 0.89 (s, 3H, C_7_-CH_3_); ^13^C-NMR δ = 173.30 (C_11_), 161.43 (C_5_), 147.52 (C_3_), 118.47 (C_4_), 42.54 (C_12_), 29.10 (C_13_), 29.03 (C_17_), 28.03 (C_6_), 27.11 (C_9_), 25.77 (C_14_), 25.48 (C_15_), 25.43 (C_16_), 23.70 (C_2_), 22.97 (C_1_), 21.50 (C_10_), 21.04 (C_7_), 14.55 (C_8_); ESI-MS *m*/*z*: 276.22 [M + H]^+^. Anal. Calcd. For C_17_H_25_NO_2_ (275.39): C, 74.14; H, 9.15; N, 5.09. Found: C, 74.16; H, 9.12; N, 5.06.

*(Z)-3-caren-5-one O-α-furylcarbonyl oxime* ((*Z*)-**4p**). White solid. Yield: 82.0%, m.p.: 114.7–115.6 °C. UV-Vis (EtOH) λ_max_ (log ε): 268.90 (4.28) nm; IR (KBr, cm^−1^): 3110 (m), 3006 (w, =C-H), 1754 (s, C=O), 1653 (m, C=N); ^1^H-NMR δ = 7.65–7.61 (m, 1H, C_15_-H), 7.22 (d, *J* = 3.5 Hz, 1H, C_13_-H), 6.55 (dd, *J* = 3.5, 1.7 Hz, 1H, C_14_-H), 6.13 (s, 1H, C_4_-H), 2.55 (dd, *J* = 20.3, 8.3 Hz, 1H, C_2_-H_a_), 2.21 (d, *J* = 20.3 Hz, 1H, C_2_-H_b_), 2.02 (d, *J* = 8.1 Hz, 1H, C_6_-H), 1.84 (s, 3H, C_3_-CH_3_), 1.33 (t, *J* = 8.1 Hz, 1H, C_1_-H), 1.28 (s, 4H, C_7_-CH_3_), 0.93 (s, 3H, C_7_-CH_3_). ^13^C-NMR δ = 162.13 (C_11_), 156.70 (C_5_), 148.25 (C_3_), 146.63 (C_12_), 143.61 (C_15_), 118.14 (C_4_), 117.95 (C_13_), 111.84 (C_14_), 28.00 (C_6_), 27.19 (C_9_), 23.77 (C_2_), 23.15 (C_1_), 21.78 (C_10_), 21.22 (C_7_), 14.54 (C_8_); ESI-MS *m*/*z*: 260.07 [M + H]^+^. Anal. Calcd. For C_15_H_17_NO_3_ (259.30): C, 69.48; H, 6.61; N, 5.40. Found: C, 69.50; H, 6.58; N, 5.41.

*(Z)-3-Caren-5-one O-α-thienylcarbonyl oxime* ((*Z*)-**4q**). White solid. Yield: 75.5%, m.p.: 108.4–109.3 °C. UV-Vis (EtOH) λ_max_ (log ε): 278.80 (4.30) nm, 253.90 (4.36) nm; IR (KBr, cm^−1^): 3116 (w), 3018 (w, =C-H), 1739 (s, C=O), 1650 (m, C=N); ^1^H-NMR δ = 7.91 (dd, *J* = 3.8, 1.2 Hz, 1H, C_13_-H), 7.59 (dd, *J* = 5.0, 1.2 Hz, 1H, C_15_-H), 7.15 (dd, *J* = 4.9, 3.8 Hz, 1H, C_14_-H), 6.14 (s, 1H, C_4_-H), 2.55 (dd, *J* = 20.6, 8.7 Hz, 1H, C_2_-H_a_), 2.21 (d, *J* = 21.1 Hz, 1H, C_2_-H_b_), 2.03 (d, *J* = 8.1 Hz, 1H, C_6_-H), 1.85 (s, 3H, C_3_-CH_3_), 1.33 (d, *J* = 8.1 Hz, 1H, C_6_-H), 1.31 (s, 3H, C_7_-CH_3_), 0.93 (s, 3H, C_7_-CH_3_). ^13^C-NMR δ = 161.82 (C_11_), 159.90 (C_5_), 148.19 (C_3_), 133.81 (C_12_), 132.45 (C_13_), 132.18 (C_15_), 127.84 (C_14_), 118.15 (C_4_), 28.00 (C_6_), 27.16 (C_9_), 23.78 (C_2_), 23.03 (C_1_), 21.70 (C_10_), 21.29 (C_7_), 14.57 (C_8_); ESI-MS *m*/*z*: 276.06 [M + H]^+^. Anal. Calcd. For C_15_H_17_NO_2_S (275.37): C, 65.43; H, 6.22; N, 5.09. Found: C, 65.45; H, 6.21; N, 5.10.

*(Z)-3-Caren-5-one O-β-pyridylcarbonyl oxime* ((*Z*)-**4r**). Brown solid. Yield: 80.0%, m.p.: 97.0–98.7 °C. UV-Vis (EtOH) λ_max_ (log ε): 262.60 (4.18) nm, 223.80 (4.14) nm; IR (KBr, cm^−1^): 3065 (w), 3039 (w, =C-H), 1743 (s, C=O), 1655 (m, C=N); ^1^H-NMR δ = 9.28 (s, 1H, C_13_-H), 8.81 (d, *J* = 4.8 Hz, 1H, C_14_-H), 8.38 (d, *J* = 7.9 Hz, 1H, C_16_-H), 7.45 (dd, *J* = 7.9, 4.9 Hz, 1H, C_15_-H), 6.16 (s, 1H, C_4_-H), 2.57 (dd, *J* = 20.3, 8.4 Hz, 1H, C_2_-H_a_), 2.23 (d, *J* = 20.3 Hz, 1H, C_2_-H_b_), 2.03 (d, *J* = 8.1 Hz, 1H, C_6_-H), 1.86 (s, 3H, C_3_-CH_3_), 1.36 (t, *J* = 8.3 Hz, 1H, C_1_-H), 1.31 (s, 3H, C_7_-CH_3_), 0.95 (s, 3H, C_7_-CH_3_). ^13^C-NMR δ = 162.88 (C_11_), 162.48 (C_5_), 153.47 (C_3_), 150.53 (C_14_), 148.73 (C_13_), 137.20 (C_16_), 125.72 (C_12_), 123.55 (C_15_), 118.06 (C_4_), 28.15 (C_6_), 27.18 (C_9_), 23.81 (C_2_), 23.21 (C_6_), 21.94 (C_10_), 21.34 (C_7_), 14.58 (C_8_); ESI-MS *m*/*z*: 271.14 [M + H]^+^. Anal. Calcd. For C_16_H_18_N_2_O_2_ (270.33): C, 71.09; H, 6.71; N, 10.36. Found: C, 71.06; H, 6.70; N, 10.38.

*(Z)-3-Caren-5-one O-(2-chloropyridylcarbonyl) oxime* ((*Z*)-**4s**). Brown solid. Yield: 82.4%, m.p.: 107.1–109.4 °C. UV-Vis (EtOH) λ_max_ (log ε): 257.50 (4.28) nm, 229.10 (4.15) nm; IR (KBr, cm^−1^): 3051 (w), 3018 (w, =C-H), 1736 (s, C=O), 1650 (m, C=N); ^1^H-NMR δ = 8.55 (dd, *J* = 4.8, 2.0 Hz, 1H, C_14_-H), 8.23 (dd, *J* = 7.7, 2.0 Hz, 1H, C_16_-H), 7.38 (dd, *J* = 7.7, 4.8 Hz, 1H, C_15_-H), 6.13 (s, 1H, C_4_-H), 2.56 (dd, *J* = 20.7, 8.7 Hz, 1H, C_2_-H_a_), 2.22 (d, *J* = 21.1 Hz, 1H, C_2_-H_b_), 2.08 (d, *J* = 8.1 Hz, 1H, C_6_-H), 1.86 (s, 3H, C_3_-CH_3_), 1.32 (t, *J* = 8.0 Hz, 1H, C_1_-H), 1.24 (s, 3H, C_7_-CH_3_), 0.94 (s, 3H, C_7_-CH_3_). ^13^C-NMR δ = 163.05 (C_11_), 162.81 (C_5_), 151.91 (C_3_), 149.48 (C_14_), 149.00 (C_13_), 140.74 (C_16_), 126.97 (C_12_), 122.24 (C_15_), 117.92 (C_4_), 28.11 (C_6_), 27.23 (C_9_), 23.80 (C_2_), 23.23 (C_6_), 22.20 (C_10_), 21.46 (C_7_), 14.56 (C_8_); ESI-MS *m*/*z*: 305.03 [M + H]^+^. Anal. Calcd. For C_16_H_17_ClN_2_O_2_ (304.77): C, 63.05; H, 5.62; N, 9.19. Found: C, 63.01; H, 5.63; N, 9.21.

*(Z)-3-Caren-5-one O-n-hexanoyl oxime* ((*Z*)-**4t**). Yellow liquid. Yield: 63.5%. UV-vis (EtOH) λ_max_ (log ε): 241.80 (4.23) nm; IR (thin film, cm^−1^): 1766 (s, C=O), 1659 (m, C=N), 763 (m), 751 (m, -(CH_2_)*_n_*-, *n* > 4); ^1^H-NMR δ = 6.06 (s, 1H, C_4_-H), 2.51 (dd, *J* = 20.3, 8.3 Hz, 1H, C_2_-H_a_), 2.45 (t, *J* = 7.5 Hz, 2H, C_12_-H), 2.18 (d, *J* = 20.3 Hz, 1H, C_2_-H_b_), 1.90 (d, *J* = 8.2 Hz, 1H, C_6_-H), 1.82 (s, 3H, C_4_-CH_3_), 1.72 (q, *J* = 7.4 Hz, 2H, C_13_-H), 1.36 (dt, *J* = 7.1, 4.4 Hz, 4H, C_14_-H, C_15_-H), 1.28 (d, *J* = 8.6 Hz, 1H, C_1_-H), 1.23 (s, 3H, C_7_-CH_3_), 0.91 (d, *J* = 7.1 Hz, 3H, C_16_-H), 0.89 (s, 3H, C_7_-CH_3_). ^13^C-NMR δ = 171.52 (C_11_), 161.20 (C_5_), 147.60 (C_3_), 118.45 (C_4_), 33.33 (C_12_), 31.32 (C_13_), 28.02 (C_6_), 27.13 (C_9_), 24.76 (C_14_), 23.70 (C_2_), 23.03 (C_1_), 22.31 (C_7_), 21.59 (C_10_), 21.00 (C_15_), 14.53 (C_8_), 13.91 (C_16_); ESI-MS *m*/*z*: 264.20 [M + H]^+^. Anal. Calcd. For C_16_H_25_NO_2_ (263.38): C, 72.96; H, 9.57; N, 5.32. Found: C, 72.92; H, 9.60; N, 5.31.

*(Z)-3-Caren-5-one O-n-propionyl oxime* ((*Z*)-**4u**). Yellow liquid. Yield: 66.3%. UV-Vis (EtOH) λ_max_ (log ε): 242.00 (4.35) nm; IR (thin film, cm^−1^): 1765 (s, C=N), 1655 (m, C=N); ^1^H-NMR δ = 6.06 (s, 1H, C_4_-H), 2.55–2.47 (m, 3H, C_2_-H_a_, C_12_-H), 2.18 (d, *J* = 20.3 Hz, 1H, C_2_-H_b_), 1.90 (d, *J* = 8.2 Hz, 1H, C_6_-H), 1.82 (s, 3H, C_3_-CH_3_), 1.29–1.24 (m, 4H, C_1_-H, C_13_-3H), 1.23 (s, 3H, C_7_-CH_3_), 0.89 (s, 3H, C_7_-CH_3_); ^13^C-NMR δ = 172.19 (C_11_), 161.23 (C_5_), 147.61 (C_3_), 118.43 (C_4_), 28.01 (C_6_), 27.14 (C_9_), 26.69 (C_12_), 23.70 (C_2_), 23.04 (C_1_), 21.59 (C_10_), 20.97 (C_7_), 14.52 (C_8_), 9.21 (C_13_); ESI-MS *m*/*z*: 222.17 [M + H] ^+^. Anal. Calcd. For C_13_H_19_NO_2_ (221.30): C, 70.56; H, 8.65; N, 6.33. Found: C, 70.59; H, 8.64; N, 6.34.

*(Z)-3-Caren-5-one O-n-butanoyl oxime* ((*Z*)-**4v**). Yellow liquid. Yield: 65.7%. UV-Vis (EtOH) λ_max_ (log ε): 243.60 (4.19) nm; IR (thin film, cm^−1^): IR (KBr, cm^−1^): 1757 (s, C=O), 1653 (m, C=N); ^1^H-NMR δ = 6.06 (d, *J* = 0.9 Hz, 1H, C_4_-H), 2.51 (dd, *J* = 20.3, 8.3 Hz, 1H, C_2_-H_a_), 2.44 (t, *J* = 7.3 Hz, 2H, C_12_-H), 2.17 (d, *J* = 20.3 Hz, 1H, C_2_-H_b_), 1.90 (d, *J* = 8.2 Hz, 1H, C_6_-H), 1.81 (s, 3H, C_3_-CH_3_), 1.75 (ddd, *J* = 14.8, 7.4, 2.7 Hz, 2H, C_13_-H), 1.27 (t, *J* = 8.2, 1H, C_1_-H), 1.23 (s, 3H, C_7_-CH_3_), 1.01 (t, *J* = 7.4 Hz, 3H, C_14_-H), 0.89 (s, 3H, C_7_-CH_3_). ^13^C-NMR δ = 171.33 (C_11_), 161.21 (C_5_), 147.62 (C_3_), 118.44 (C_4_), 35.22 (C_12_), 28.01 (C_6_), 27.13 (C_9_), 23.70 (C_2_), 23.03 (C_1_), 21.58 (C_10_), 21.00 (C_7_), 18.54 (C_13_), 14.52 (C_8_), 13.76 (C_14_); ESI-MS *m*/*z*: 236.19 [M + H]^+^. Anal. Calcd. For C_14_H_21_NO_2_ (235.32): C, 71.46; H, 8.99; N, 5.95. Found: C, 71.44; H, 8.97; N, 5.93.

*(Z)-3-Caren-5-one O-n-pentanoyl oxime* ((*Z*)-**4w**). Yellow liquid. Yield: 60.0%. UV-Vis (EtOH) λ_max_ (log ε): 243.40 (4.32) nm; IR (thin film, cm^−1^): 1769 (s, C=O), 1656 (m, C=N), 763 (m), 733 (m, -(CH_2_)*_n_*-, *n* ≥ 4); ^1^H-NMR δ = 6.06 (s, 1H, C_4_-H), 2.51 (dd, *J* = 20.3, 7.7 Hz, 1H, C_2_-H_a_), 2.46 (t, 2H, C_12_-H), 2.18 (d, *J* = 19.6 Hz, 1H, C_2_-H_b_), 1.90 (d, *J* = 8.2 Hz, 1H, C_6_-H), 1.81 (s, 3H, C_3_-CH_3_), 1.73–1.68 (m, 2H, C_13_-H), 1.41 (dt, *J* = 14.8, 7.4 Hz, 2H, C_14_-H), 1.27 (t, *J* = 8.5 Hz, 1H, C_1_-H), 1.23 (s, 3H, C_7_-CH_3_), 0.94 (t, *J* = 7.4 Hz, 3H, C_15_-H), 0.89 (s, 3H, C_7_-CH_3_). ^13^C-NMR δ = 171.51 (C_11_), 161.20 (C_5_), 147.61 (C_3_), 118.44 (C_4_), 33.06 (C_12_), 28.02 (C_6_), 27.13 (C_9_, C_13_), 23.70 (C_2_), 23.03 (C_1_), 22.29 (C_7_), 21.59 (C_10_), 21.01 (C_14_), 14.53 (C_8_), 13.72 (C_15_); ESI-MS *m*/*z*: 250.08 [M + H]^+^. Anal. Calcd. For C_15_H_23_NO_2_ (249.35): C, 72.25; H, 9.30; N, 5.62. Found: C, 72.23; H, 9.31; N, 5.60.

*(E)-3-Caren-5-one O-(4-fluorobenzoyl) oxime* ((*E*)-**4f′**). Pale yellow liqiud, yield: 60.0%. UV-Vis (EtOH) λ_max_ (log ε): 256.00 (3.97) nm, 229.70 (4.21) nm; IR (thin film, cm^−1^): 3078, 3009 (w, Ar-H, =C-H), 1748 (s, C=O), 1679 (m, C=N); ^1^H-NMR δ = 8.11 (dd, *J* = 8.8, 5.5 Hz, 2H, C_13_-H, C_17_-H), 7.15 (t, *J* = 8.6 Hz, 2H, C_14_-H, C_16_-H), 6.61 (s, 1H, C_4_-H), 2.58 (dd, *J* = 20.7, 8.0 Hz, 1H, C_2_-H_a_), 2.27 (d, *J* = 20.7 Hz, 1H, C_2_-H_b_), 1.91 (s, 3H, C_3_-CH_3_), 1.84 (d, *J* = 8.6 Hz, 1H, C_6_-H), 1.33 (d, *J* = 8.0 Hz, 1H, C_1_-H), 1.19 (s, 3H, C_7_-CH_3_), 0.94 (s, 3H, C_7_-CH_3_); ^13^C- NMR δ 166.60 (C_11_), 164.92 (C_5_), 163.10 (C_15_), 159.43 (C_12_), 152.56 (C_3_), 132.06 (C_13_ or C_17_), 125.84 (C_13_ or C_17_), 115.72 (C_14_ or C_16_), 115.58 (C_14_ or C_16_), 113.16 (C_4_), 28.12 (C_6_), 28.01 (C_9_), 24.23 (C_2_), 23.77 (C_1_), 22.35 (C_10_), 20.57 (C_7_), 14.13 (C_8_); ESI-MS *m*/*z*: 288.16 [M + H]^+^. Anal. Calcd. For C_17_H_18_FNO_2_ (287.33): C, 71.06; H, 6.31; N, 4.87. Found: C, 71.02; H, 6.32; N, 4.85.

*(E)-3-Caren-5-one O-(2, 3-dichlorobenzoyl) oxime* ((*E*)-**4l′**). Yellow liquid. Yield: 60.8%. UV-Vis (EtOH) λ_max_ (log ε):253.00 (4.26) nm; IR (thin film, cm^−1^): 3071, 3009 (w, Ar-H, =C-H), 1759 (s, C=O), 1649 (m, C=N); ^1^H-NMR δ = 7.67 (dd, *J* = 7.7, 1.5 Hz, 1H, C_17_-H), 7.61 (dd, *J* = 8.0, 1.5 Hz, 1H, C_15_-H), 7.30 (t, *J* = 7.9 Hz, 1H, C_16_-H), 6.58 (s, 1H, C_4_-H), 2.57 (dd, *J* = 20.8, 8.1 Hz, 1H, C_5_-H_a_), 2.26 (d, *J* = 20.8 Hz, 1H, C_5_-H_b_), 1.87 (s, 3H, C_3_-CH_3_), 1.82 (d, *J* = 8.5 Hz, 1H, C_6_-H), 1.35 (t, *J* = 8.3 Hz, 1H, C_1_-H), 1.19 (s, 3H, C_7_-CH_3_), 0.93 (s, 3H, C_7_-CH_3_);^13^C-NMR δ = 163.37 (C_11_), 159.97 (C_2_), 153.10 (C_3_), 134.40 (C_12_), 133.00 (C_15_), 132.86 (C_14_), 131.27 (C_13_), 129.12 (C_17_), 127.32 (C_16_), 113.42 (C_4_), 28.11 (C_1_), 28.01 (C_9_), 24.17 (C_5_), 23.67 (C_6_), 22.47 (C_10_), 20.68 (C_7_), 14.11 (C_8_); ESI-MS *m*/*z*: 337.83 [M − H]^-^. . For C_17_H_17_Cl_2_NO_2_ (338.23): C, 60.37; H, 5.07; N, 4.14. Found: C, 60.39; H, 5.06; N, 4.13.

*(E)-3-Caren-5-one O-β-pyridylcarbonyl oxime* ((*E*)-**4r′**)*.* White solid. Yield 78.3%, m.p.: 108.5–109.3 °C. UV-Vis (EtOH) λ_max_ (log ε): 262.50 (4.07) nm, 223.40 (4.12) nm; IR (KBr, cm^−1^): 3092, 3057 (w, Ar-H, =C-H), 1750 (s, C=O), 1635 (m, C=N); ^1^H-NMR δ = 9.29 (s, 1H, C_13_-H), 8.80 (d, *J* = 4.8 Hz, 1H, C_14_-H), 8.38 (d, *J* = 7.9 Hz, 1H, C_16_-H), 7.45 (dd, *J* = 8.3, 5.2 Hz, 1H, C_15_-H), 6.63 (s, 1H, C_4_-H), 2.60 (dd, *J* = 20.8, 8.1 Hz, 1H, C_2_-H_a_), 2.29 (d, *J* = 20.8 Hz, 1H, C_2_-H_b_), 1.92 (s, 3H, C_3_-CH_3_), 1.84 (d, *J* = 8.5 Hz, 1H, C_3_-CH_3_), 1.36 (t, *J* = 8.2 Hz, 1H, C_1_-H), 1.20 (s, 3H, C_7_-CH_3_), 0.95 (s, 3H, C_7_-CH_3_); ^13^C-NMR δ = 162.71 (C_11_), 159.90 (C_5_), 153.42 (C_3_), 153.19 (C_14_), 150.55 (C_13_), 137.24 (C_16_), 125.77 (C_12_), 123.51 (C_15_), 113.03 (C_4_), 28.15 (C_6_), 28.01 (C_9_), 24.24 (C_2_), 23.74 (C_6_), 22.43 (C_10_), 20.68 (C_7_), 14.13 (C_8_); ESI-MS *m*/*z*: 270.96 [M + H]^+^. Anal. Calcd. For C_16_H_18_N_2_O_2_ (270.33): C, 71.09; H, 6.71; N, 10.36. Found: C, 71.11; H, 6.69; N, 10.37.

### 3.6. Antifungal Activity Test

Antifungal activity of the target compounds was perform in vitro method [40]. The tested compound was dissolved in acetone. Sorporl-144 (200 μg/mL) emulsifier was added to dilute the solution to 500 μg/mL. Then, 1 mL solution of the tested compound was poured into a culture plate, and then 9 mL PSA culture medium was added to obtain the flat containing 50 μg/mL tested compound. A bacterium tray of 5-mm diameter cut along the external edge of the mycelium was transferred to the flat containing the tested compound and put in equilateral triangular style in duplicate. Later, the culture plate was cultured at 24 ± 1 °C and the expanded diameter of the bacterium tray was measured after 48 h and compared with that treated with aseptic distilled water to calculate the relative inhibition percentage:

Relative inhibitory rate (%) = (*CK* − *PT*)/*CK* × 100%

where *CK* is the extended diameter of the circle of mycelium during the blank assay and *PT* is the extended diameter of the circle of mycelium during testing.

## 4. Conclusions

Twenty-four novel 3-caren-5-one oxime esters were designed, synthesized, characterized, and evaluated for their antifungal activity. As a result, at 50 µg/mL, the target compounds exhibited best antifungal activity against *P. piricola*, in which compounds (*Z*)-**4r**, (*Z*)-**4q**, (*E*)-**4f′**, (*Z*)-**4i**, (*Z*)-**4j**, and (*Z*)-**4p** had inhibition rates of 97.1%, 87.4%, 87.4%, 85.0%, 81.9% and 77.7%, respectively, showing better antifungal activity than that of the commercial fungicide chlorothanil. Also, compound (*Z*)-**4r** displayed remarkable antifungal activity against all the tested fungi, with inhibition rates of 76.7%, 82.7%, 97.1%, 66.3%, 74.7%, 93.9%, 76.7% and 93.3%, respectively, showing better or comparable antifungal activity than that of the commercial fungicide chlorothanil. Thus, compound (*Z*)-**4r** is a lead compound worthy of further investigation. Besides, *E-Z* isomers of the target oxime esters were found to show obvious difference in antifungal activity. It is very meaningful and require further studies in photoisomerization and drug resistance.

## Figures and Tables

**Table 1:** Antifungal activity of the target compounds (*Z*)-**4a**–**4w** and (*E*)-**4f′** at 50 μg/mL.

Compounds	Relative Inhibition Rate (%) against the Tested Fungi							
	*F. oxysporum* f. sp. *cucumerinum*	*C. arachidicola*	*P. piricola*	*A. solani*	*G. zeae*	*R. solani*	*B. myadis*	*C. orbicalare*
(*Z*)-**4a** (R = Ph)	28.8	35.0	69.4	13.7	21.8	0	32.2	43.3
(*Z*)-**4b** (R = *o*-ClPh)	16.3	30.0	0	0	21.8	18.3	23.9	30.5
(*Z*)-**4c** (R = *m*-ClPh)	22.5	0	44.4	0	21.8	17.1	26.7	33.1
(*Z*)-**4d** (R = *p*-ClPh)	28.8	0	28.8	0	19.8	0	29.4	33.1
(*Z*)-**4e** (R = *o*-FPh)	31.9	45.0	41.3	35.9	37.5	35.0	26.7	45.9
(*Z*)-**4f** (R = *p*-FPh)	25.6	15.0	30.7	0.0	15.9	0	26.7	23.8
(*Z*)-**4g** (R = *o*-OMePh)	25.6	0	60.0	21.1	13.9	0	26.7	35.6
(*Z*)-**4h** (R = *o*-MePh)	31.9	50.0	28.8	21.1	29.6	38.6	29.4	43.3
(*Z*)-**4i** (R = *m*-MePh)	19.4	15.0	85.0	0	12.0	0	23.9	0
(*Z*)-**4j** (R = *p*-MePh)	38.1	20.0	81.9	24.8	17.8	0	35.0	53.6
(*Z*)-**4k** (R = 2,4-ClPh)	28.8	55.0	16.3	13.7	17.8	18.3	23.9	53.6
(*Z*)-**4l** (R = 2,3-ClPh)	25.6	15.0	28.8	17.4	19.8	0	21.1	43.3
(*Z*)-**4m** (R = *p*-CH_2_ClPh)	56.9	20.0	41.3	0	19.8	0	21.1	40.8
(*Z*)-**4n** (R = cyclo-pentyl)	22.5	35.0	0	13.7	19.8	0	23.9	51.0
(*Z*)-**4o** (R = cyclo-hexyl)	25.6	30.0	28.8	21.1	15.9	16.0	23.9	40.8
(*Z*)-**4p** (R = *α*-furyl)	60.0	64.5	77.7	53.8	36.5	54.6	43.3	54.4
(*Z*)-**4q** (R = *α*-thienyl)	48.9	37.3	87.4	41.3	33.5	42.1	33.8	48.9
(*Z*)-**4r** (R = *β*-pyridyl)	76.7	82.7	97.1	66.3	74.7	93.9	76.7	93.3
(*Z*)-**4s** (R = *α*-Cl-*β*-pyridyl)	15.6	37.3	55.2	41.3	54.1	63.6	24.3	21.1
(*Z*)-**4t** (R = *n*-pentyl)	21.1	19.1	58.4	35.0	48.2	67.1	24.3	21.1
(*Z*)-**4u** (R = *n*-ethyl)	15.6	19.1	58.4	28.8	48.2	36.8	29.0	21.1
(*Z*)-**4v** (R = *n*-propyl)	15.6	28.2	32.6	41.3	57.1	54.6	24.3	21.1
(*Z*)-**4w** (R = *n*-butyl)	15.6	19.1	51.9	22.5	18.8	18.9	19.5	21.1
(*E*)-**4f′** (R = *p*-FPh)	60.0	64.5	87.4	53.8	65.9	76.1	76.7	54.4
(*Z*)-**3a**	15.6	23.6	51.9	35.0	18.8	24.3	24.3	26.7
(*E*)-**3b**	15.6	23.6	51.9	22.5	18.8	27.9	19.5	21.1
**2**	21.1	19.1	45.5	16.3	24.7	18.9	24.3	15.6
Chlorothanil	100	73.3	75.0	73.9	73.1	96.1	90.4	91.3

## Supplementary Materials

Supplementary materials are available online.
