# Supplementary material for: Synthesis and Antifungal Activity of Novel 3-Caren-5-One Oxime Esters
Source: Molecules. 2017 Sep 12;22(9):1538. doi: 10.3390/molecules22091538 (PMC6151701; doi:10.3390/molecules22091538)
Supplement: Supplementary file 1 [file molecules-22-01538-s001.pdf]

Supplementary Materials: Synthesis and Antifungal Activity of 3-caren-5-one oxime esters

Min Huang, Wen-Gui Duan *, Gui-Shan Lin *, Kun Li and Qiong Hu

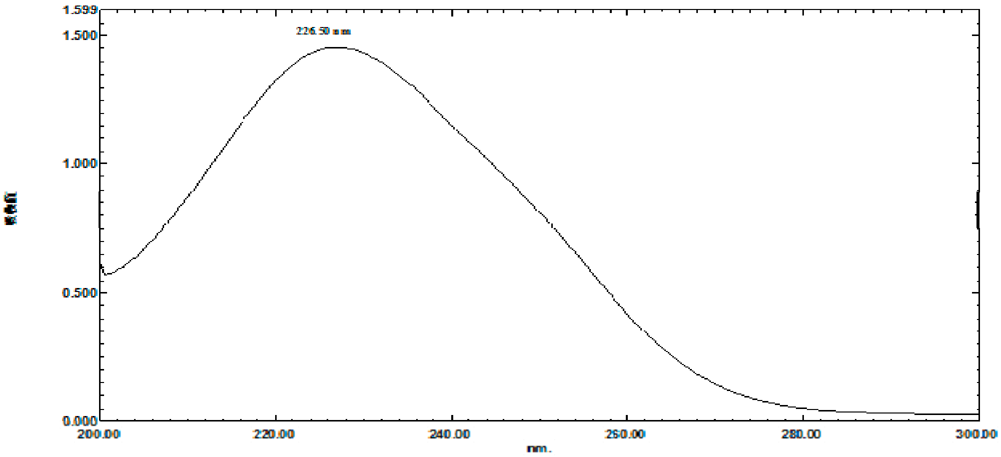

Figure S1. UV-vis spectrum of 3-caren-5-one (**2**) in EtOH.

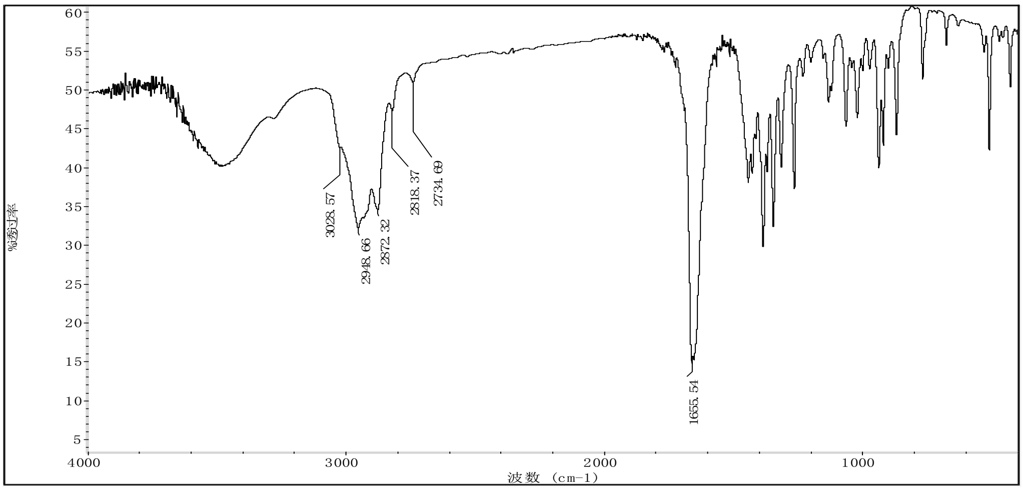

Figure S2. FTIR spectrum of 3-caren-5-one (**2**).

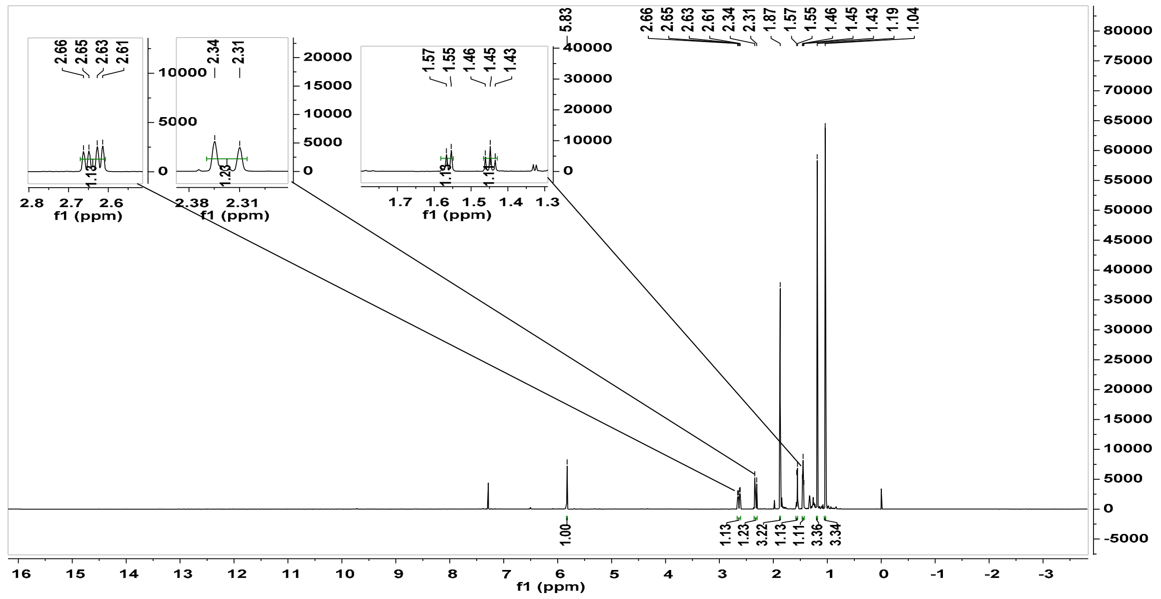

Figure S3. ^{1}H-NMR spectrum of 3-caren-5-one (**2**) in $CDCl_3$.

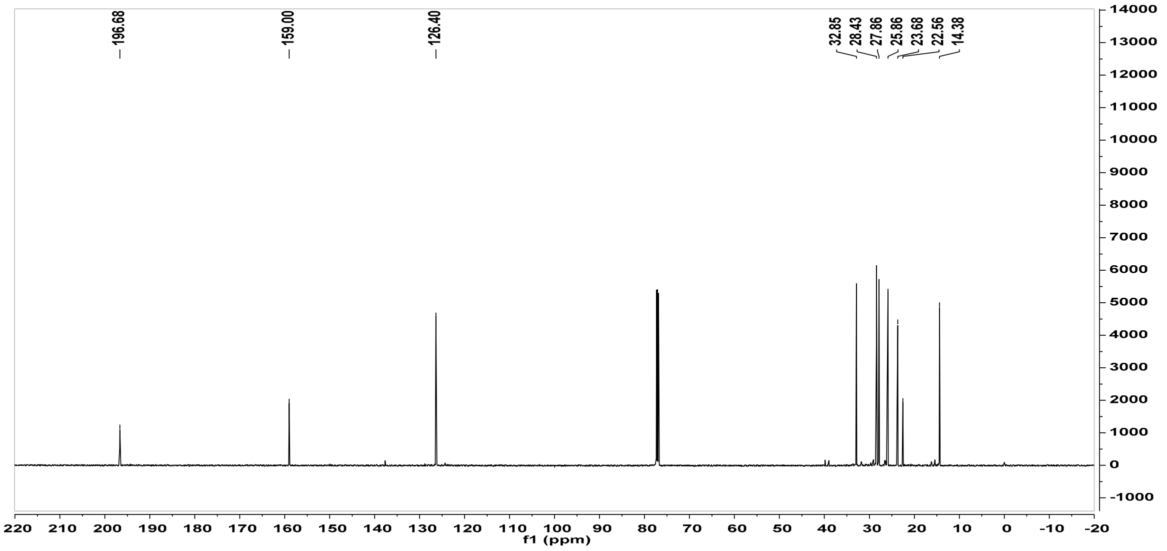

Figure S4. ^{13}C-NMR spectrum of 3-caren-5-one (**2**) in $CDCl_3$.

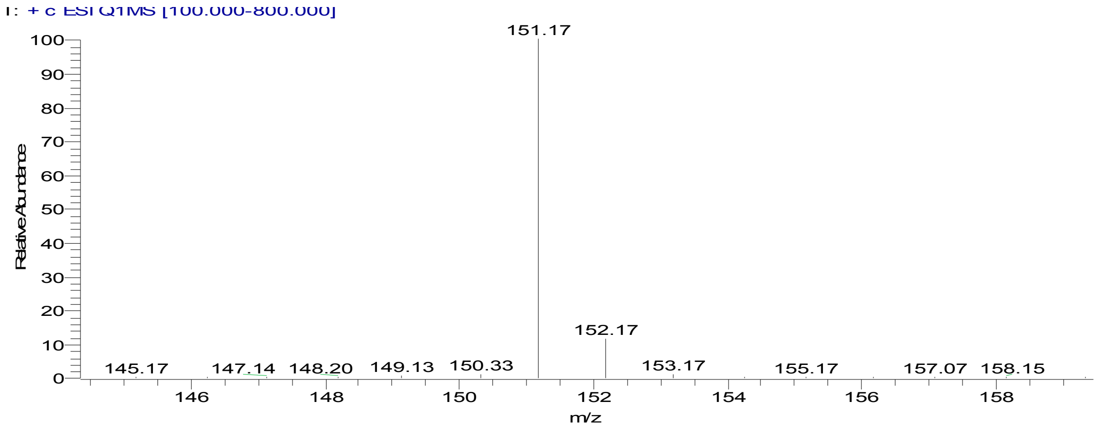

Figure S5. ESI-MS spectrum of 3-caren-5-one (**2**).

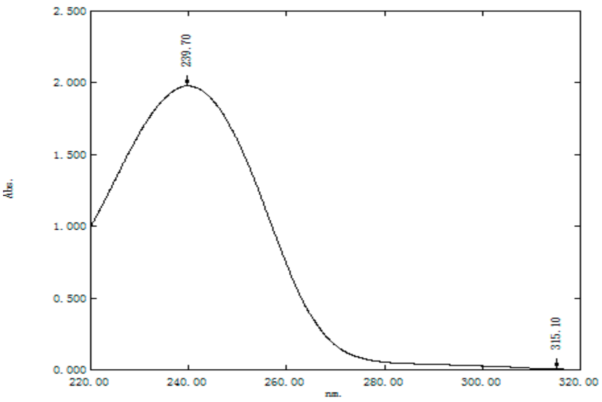

Figure S6. UV-vis spectrum of (*Z*)-3-caren-5-one oxime ((*Z*)-**3a**) in EtOH.

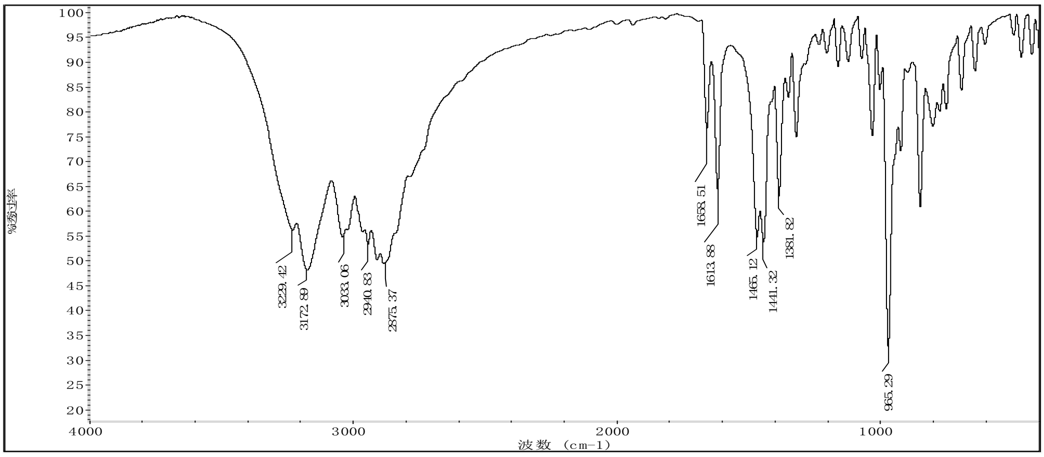

Figure S7. FTIR spectrum of (*Z*)-3-caren-5-one oxime ((*Z*)-**3a**).

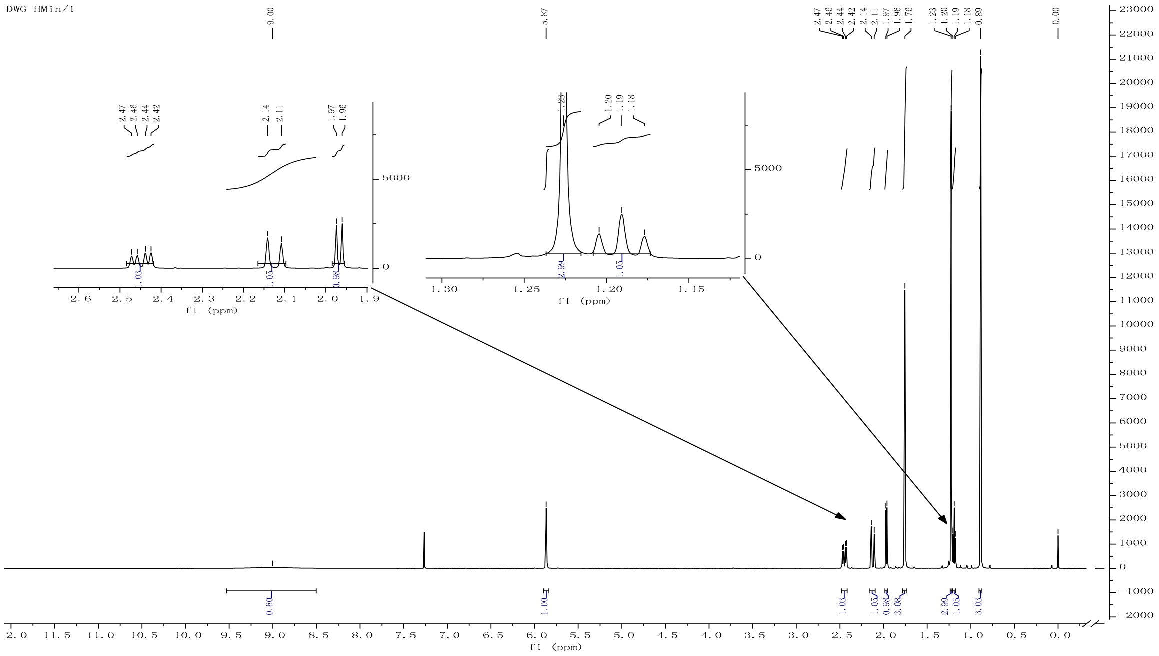

Figure S8. ^{1}H-NMR spectrum of (*Z*)-3-caren-5-one oxime ((*Z*)-**3a**) in $CDCl_3$.

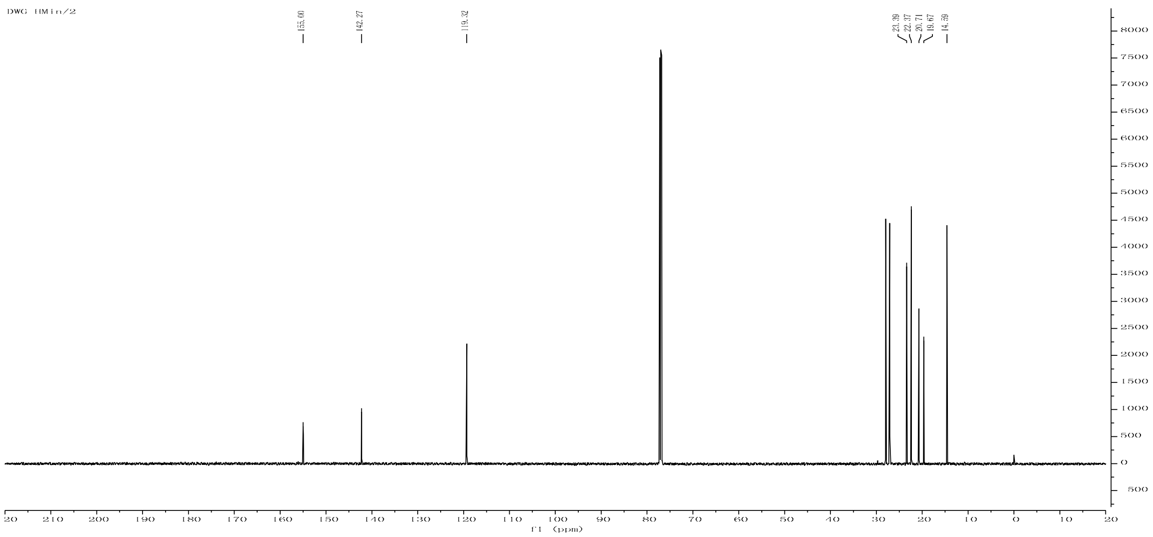

Figure S9. ^{13}C-NMR spectrum of (*Z*)-3-caren-5-one oxime ((*Z*)-**3a**) in $CDCl_3$.

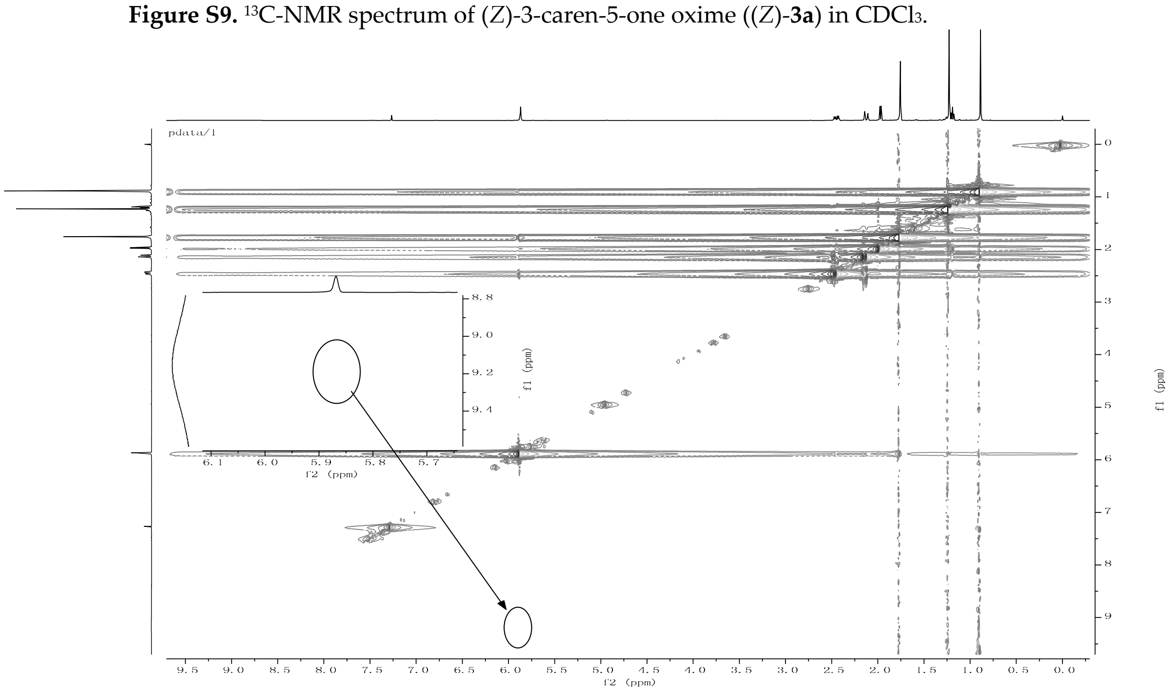

Figure S10. NOESY spectrum of (*Z*)-3-caren-5-one oxime ((*Z*)-**3a**) in $CDCl_3$.

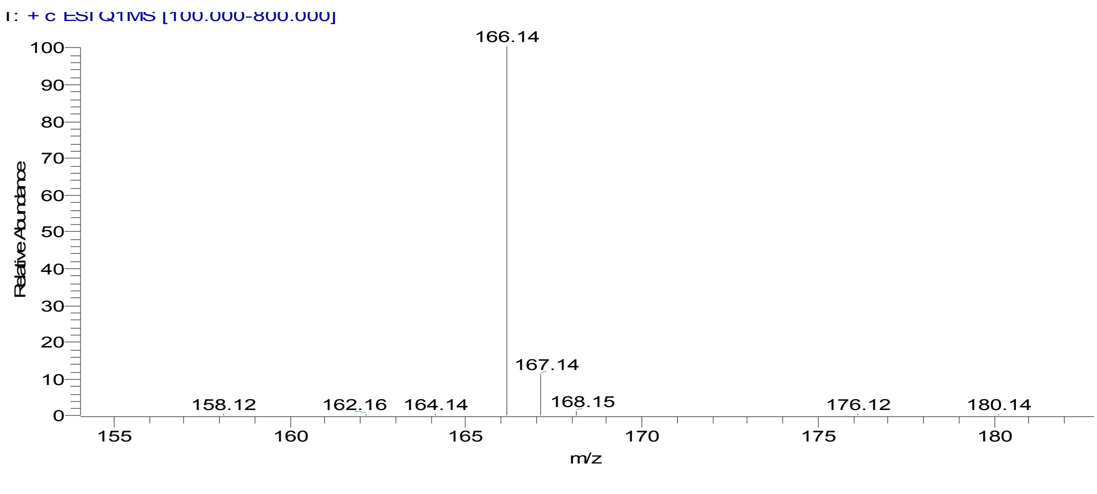

Figure S11. ESI-MS spectrum of (*Z*)-3-caren-5-one oxime ((*Z*)-**3a**).

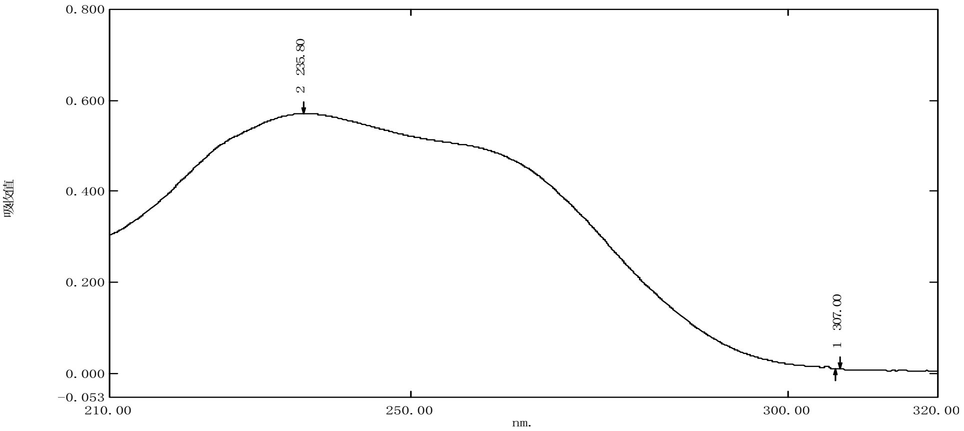

Figure S12. UV-vis spectrum of (*E*)-3-carene-5-one oxime ((*E*)-**3b**) in EtOH.

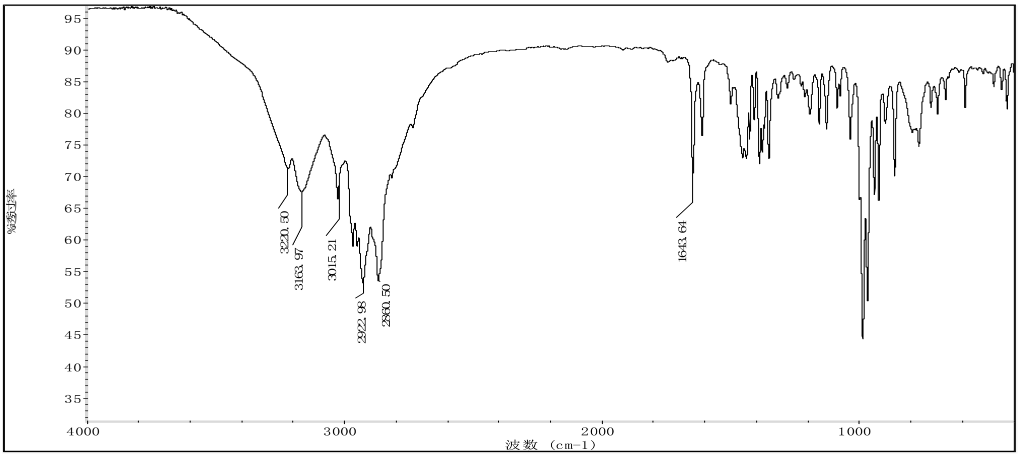

Figure S13. FTIR spectrum of (*E*)-3-caren-5-one oxime ((*E*)-**3b**).

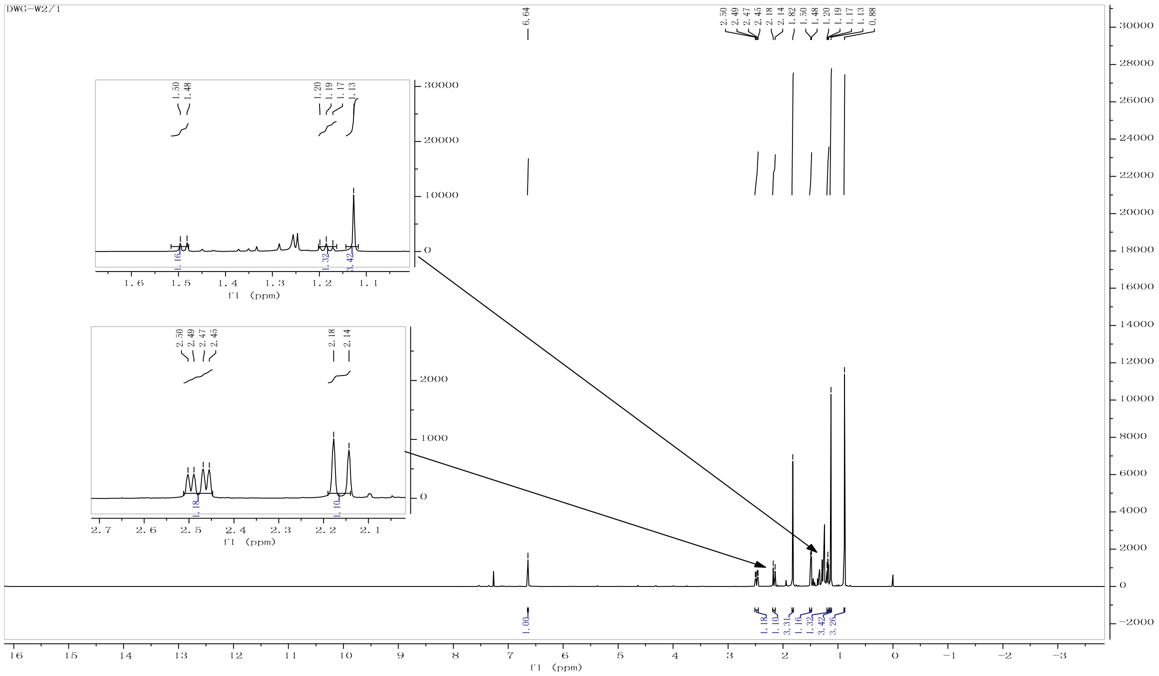

Figure S14. ^{1}H-NMR spectrum of (*E*)-3-caren-5-one oxime ((*E*)-**3b**) in $CDCl_3$.

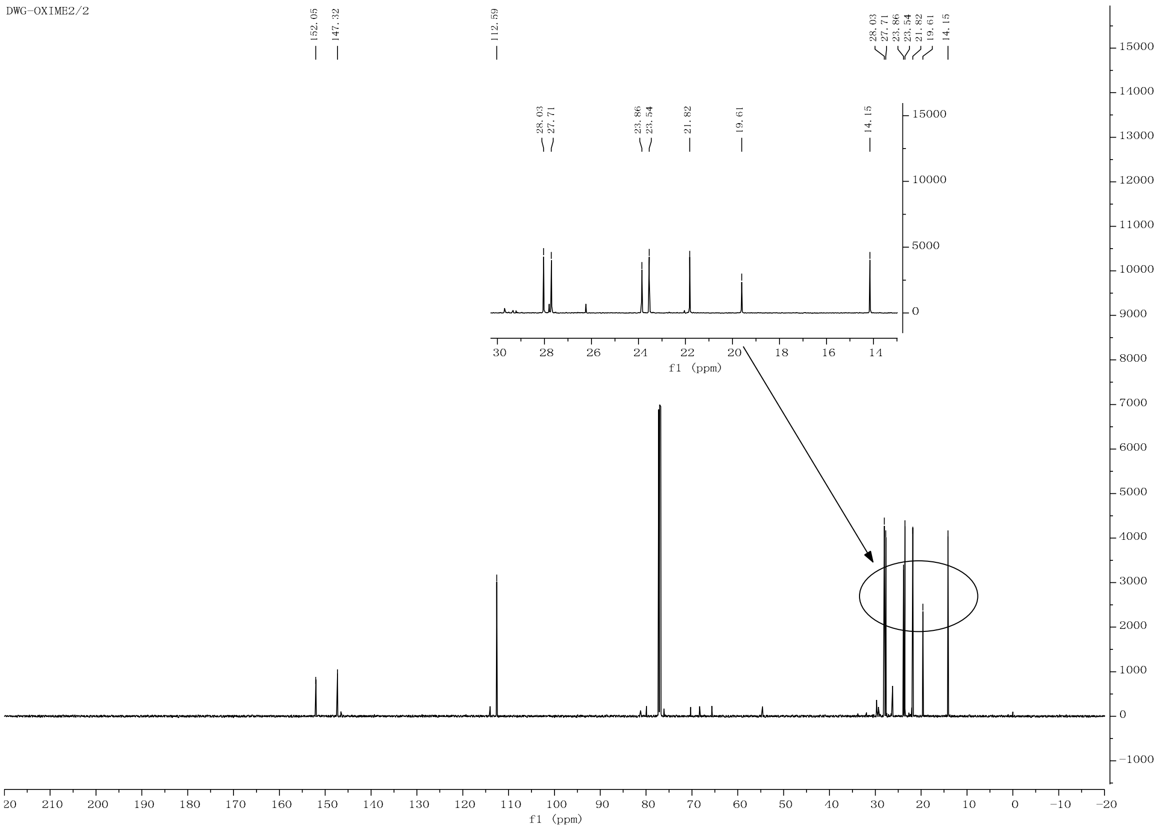

Figure S15. ^{13}C-NMR spectrum of (*E*)-3-caren-5-one oxime ((*E*)-**3b**) in $CDCl_3$

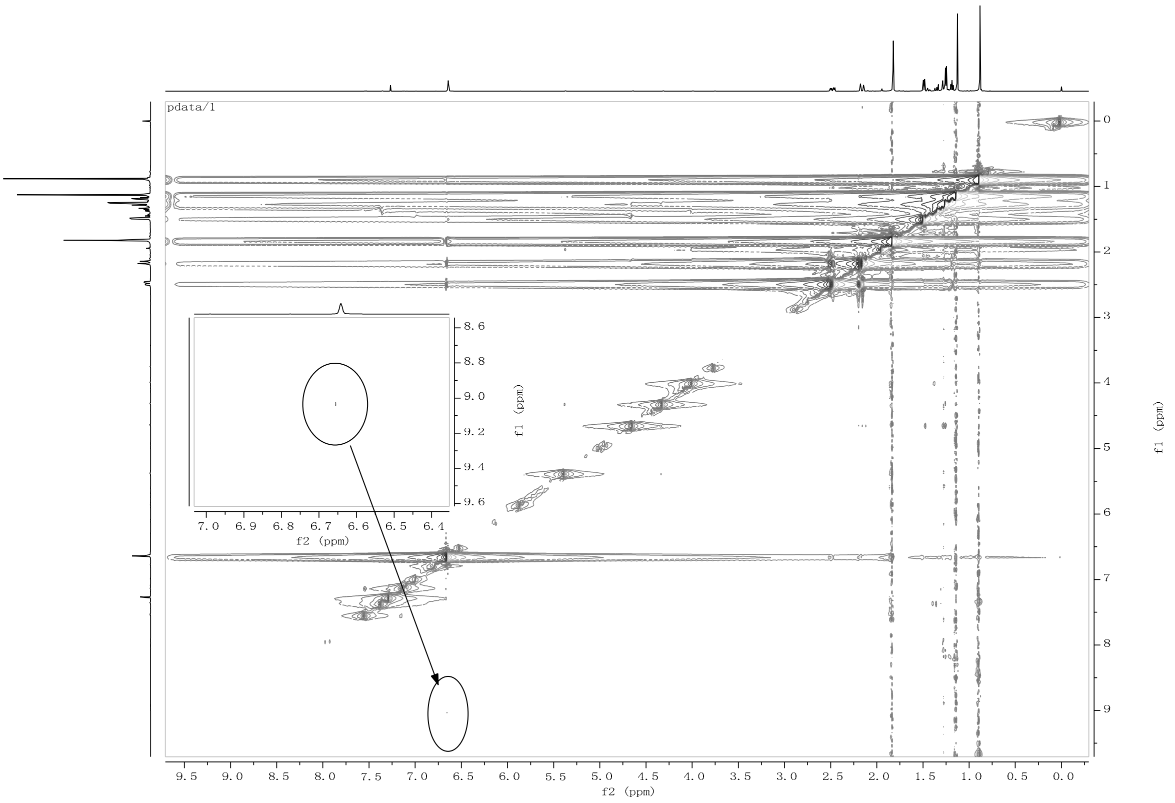

Figure S16. NOESY spectrum of (*E*)-3-caren-5-one oxime ((*E*)-**3b**) in $CDCl_3$.

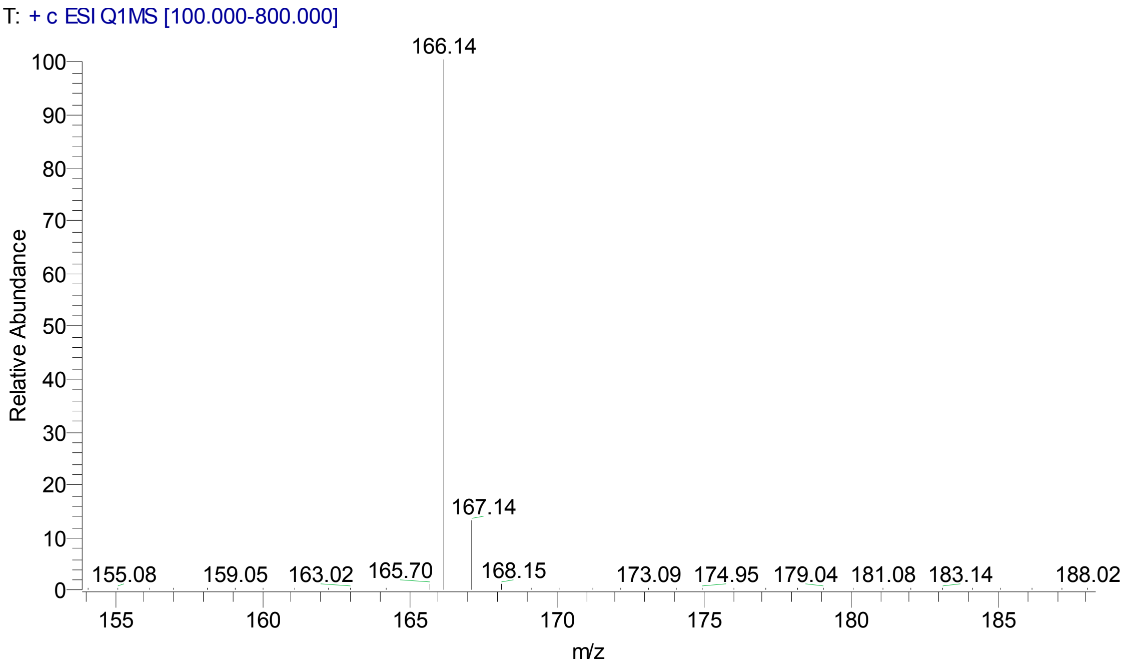

Figure S17. ESI-MS spectrum of (*E*)-3-caren-5-one oxime ((*E*)-**3b).**

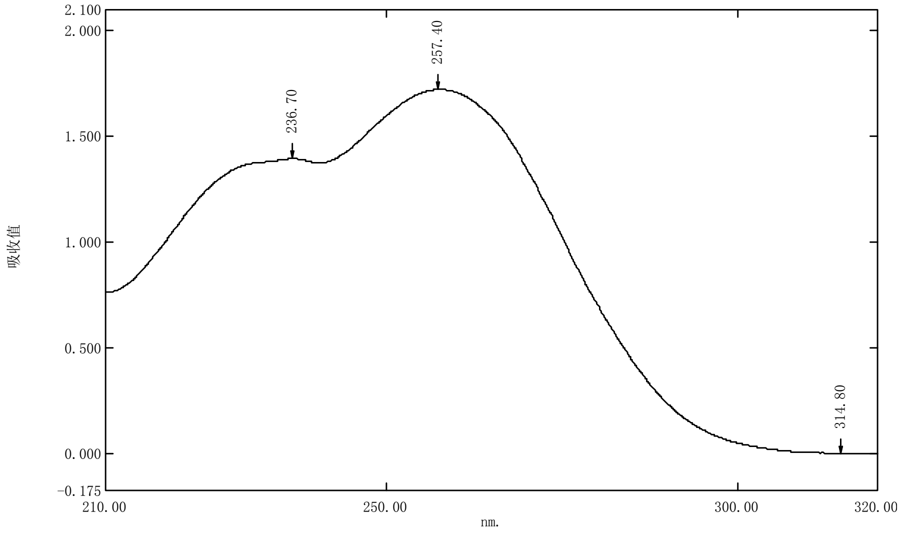

Figure S18. UV-vis spectrum of compound (*Z*)-**4a** in EtOH.

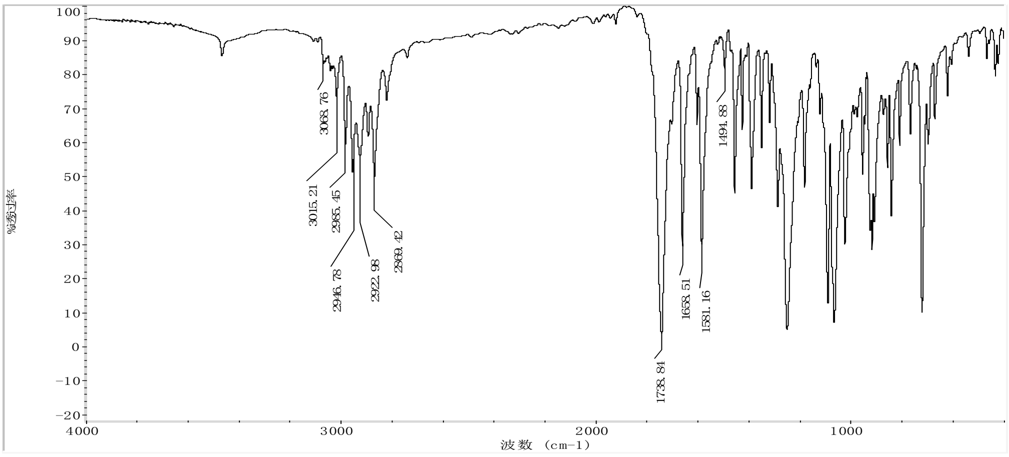

Figure S19. FTIR spectrum of compound (*Z*)-**4a**.

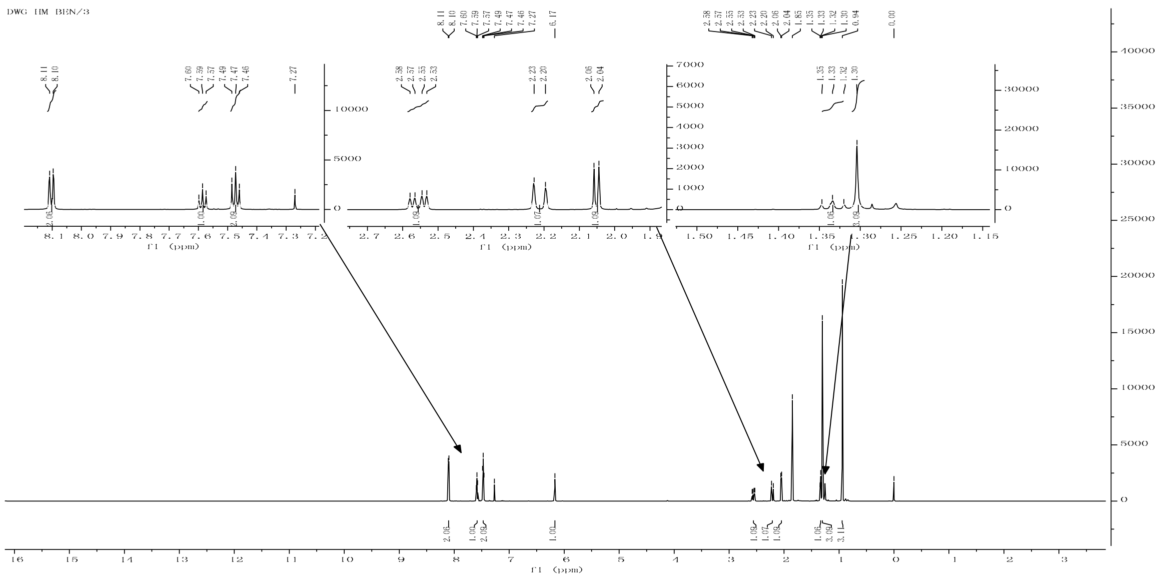

Figure S20. ^{1}H-NMR spectrum of compound (*Z*)-**4a** in $CDCl_3$.

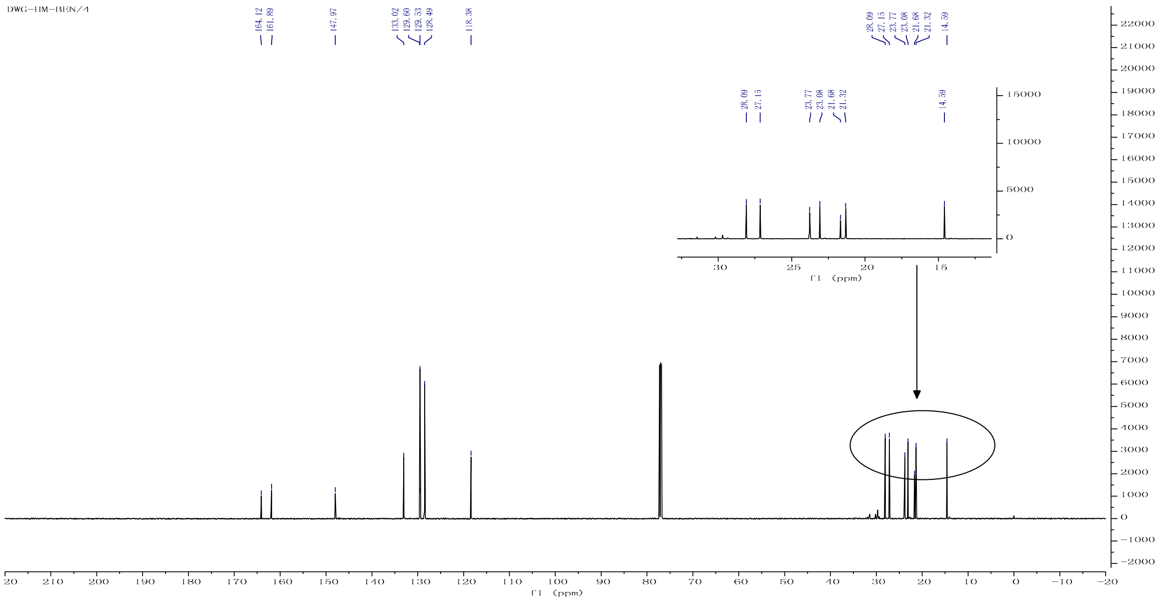

Figure S21. ^{13}C-NMR spectrum of compound (*Z*)-**4a** in $CDCl_3$.

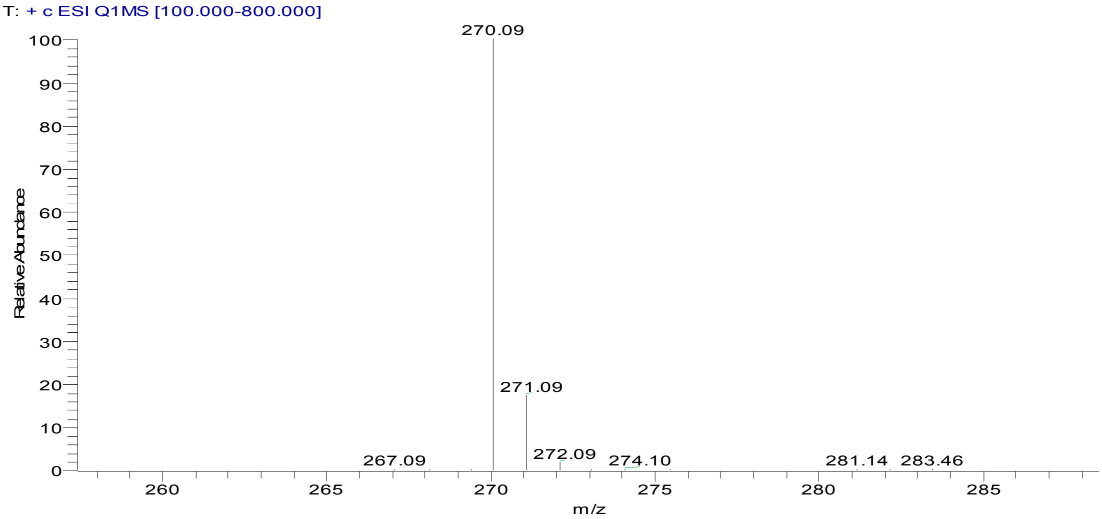

Figure S22. ESI-MS spectrum of compound (*Z*)-**4a.**

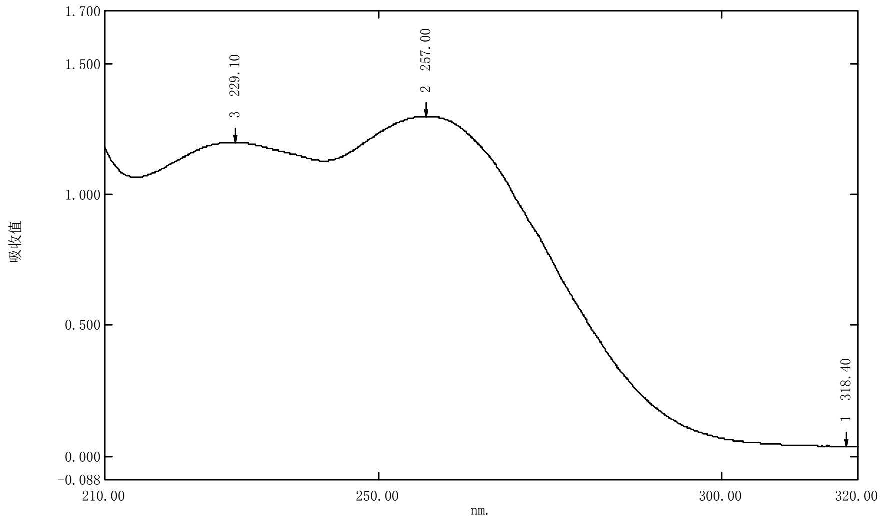

Figure S23. UV-vis spectrum of compound (*Z*)-**4b** in EtOH.

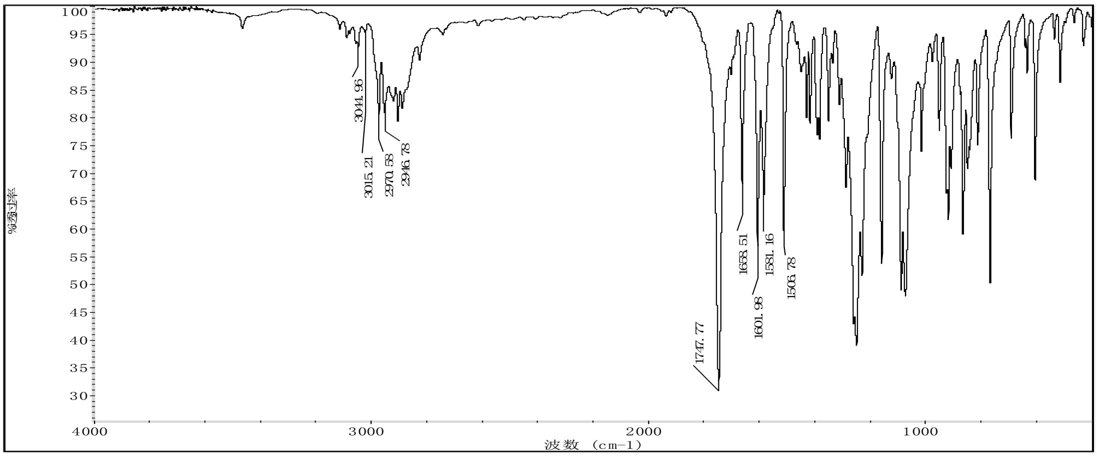

Figure S24. FTIR spectrum of compound (*Z*)-**4b**.

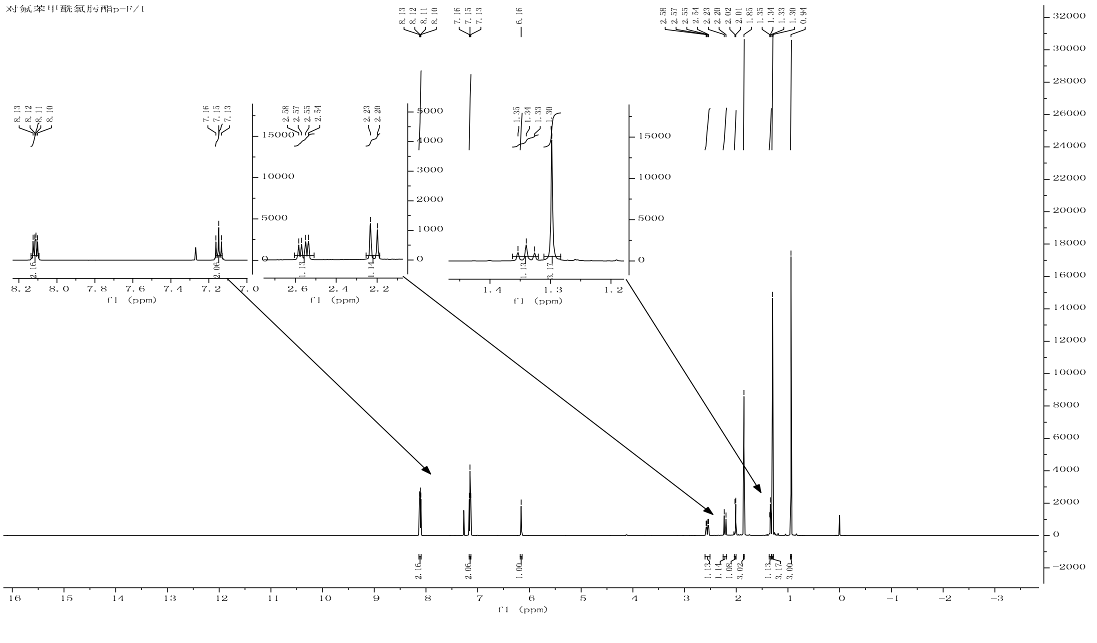

Figure S25. ^{1}H-NMR spectrum of compound (*Z*)-**4b** in $CDCl_3$.

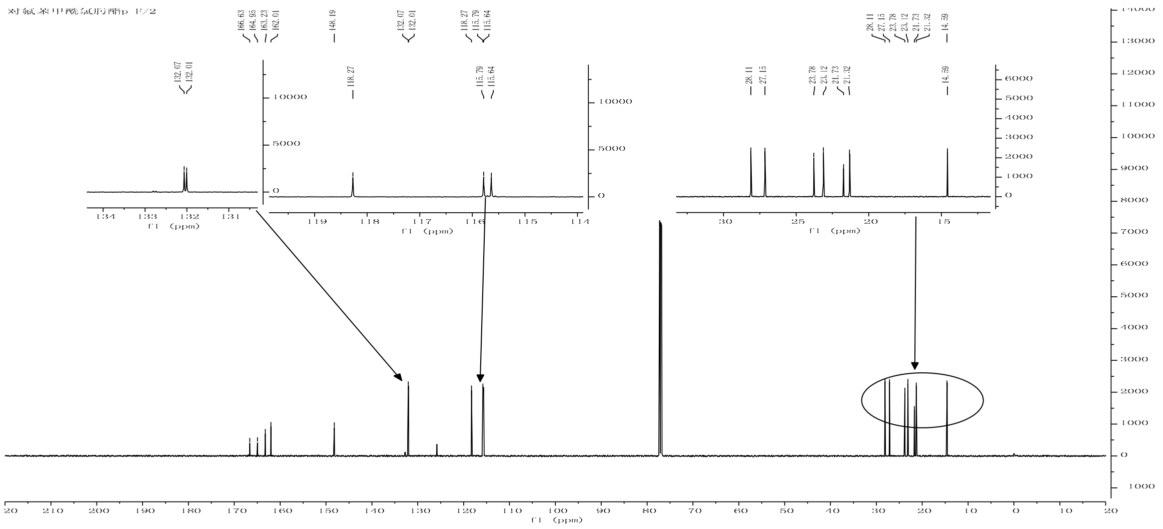

Figure S26. ^{13}C-NMR spectrum of compound (*Z*)-**4b** in $CDCl_3$.

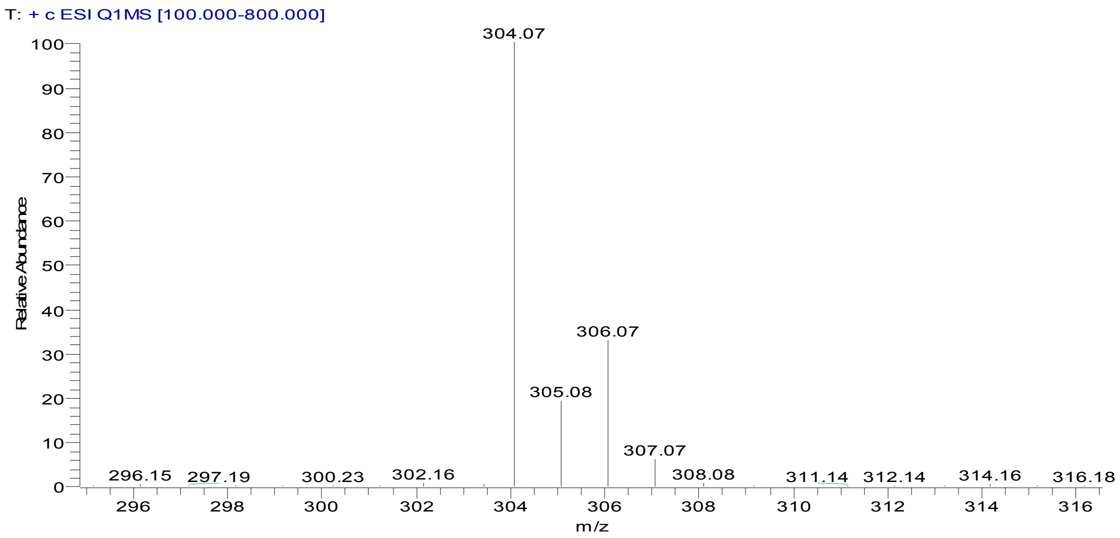

Figure S27. ESI-MS spectrum of compound (*Z*)-**4b.**

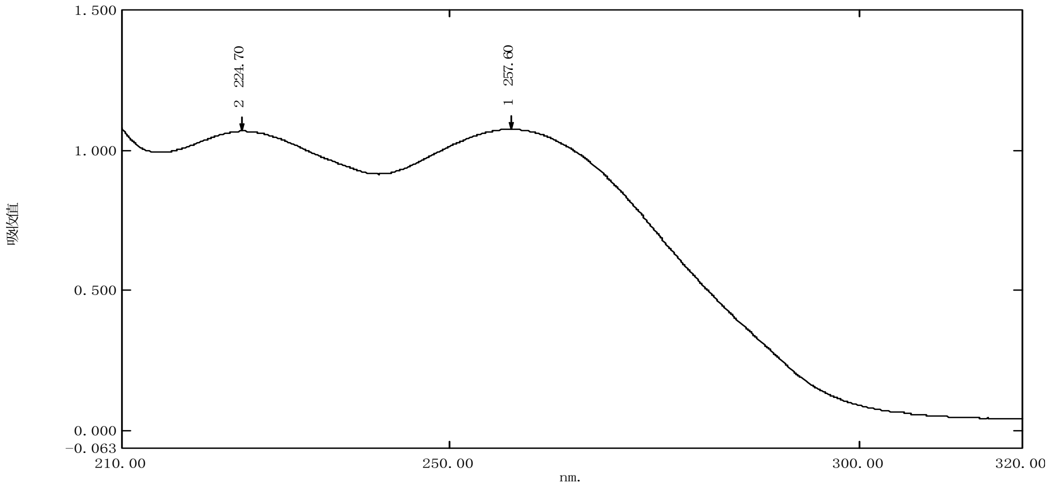

Figure S28. UV-vis spectrum of compound (*Z*)-**4c** in EtOH.

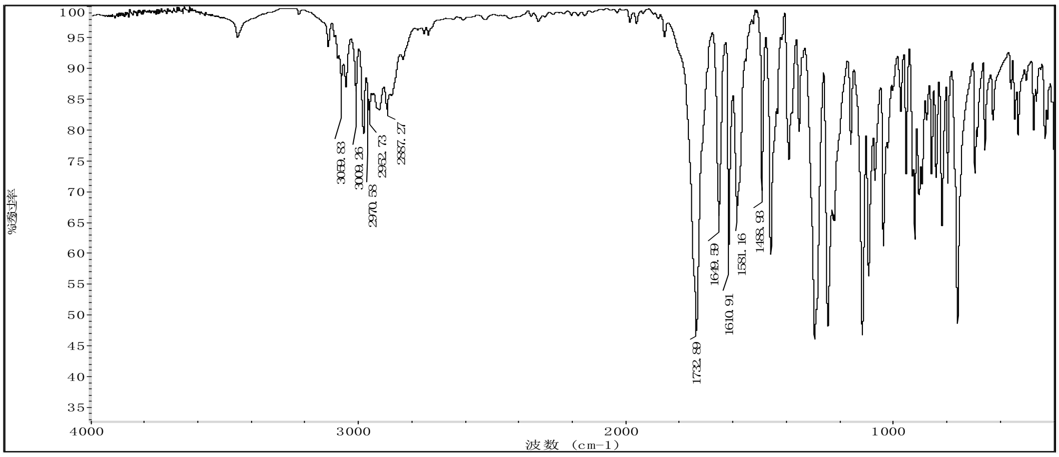

Figure S29. FTIR spectrum of compound (*Z*)-**4c**.

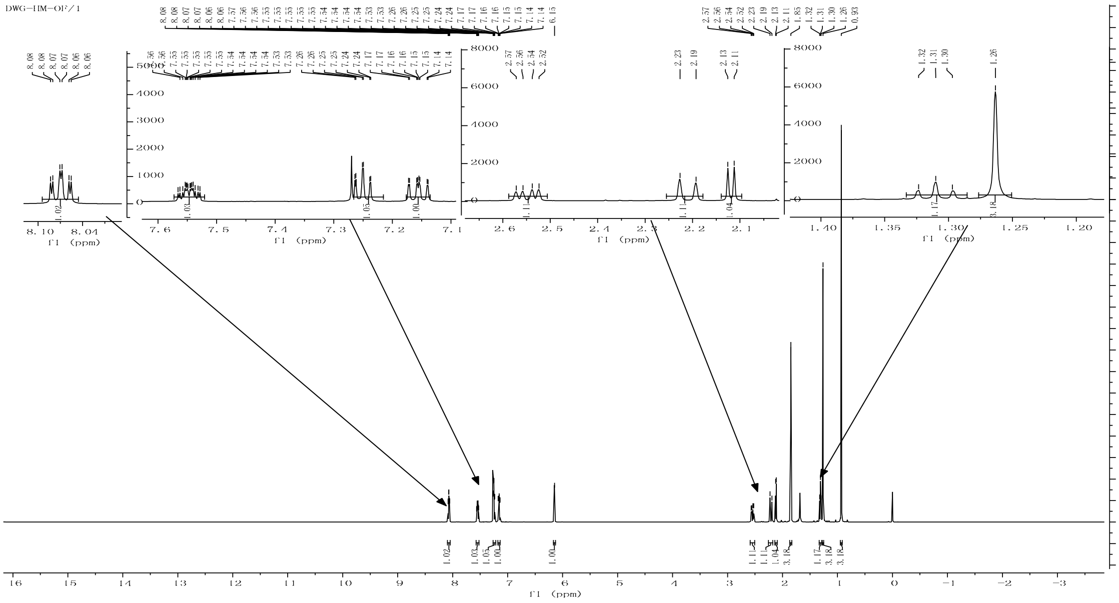

Figure S30. ^{1}H-NMR spectrum of compound (*Z*)-**4c** in $CDCl_3$.

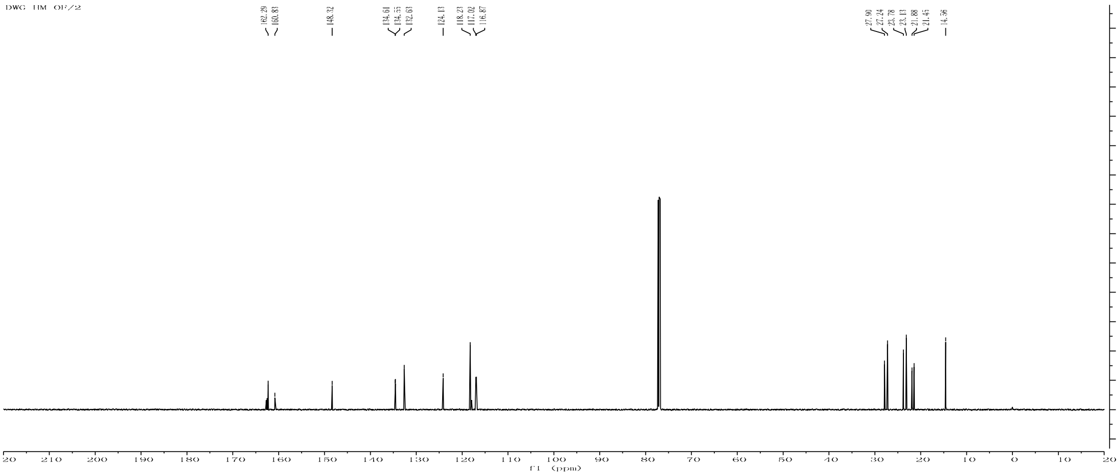

Figure S31. ^{13}C-NMR spectrum of compound (*Z*)-**4c** in $CDCl_3$.

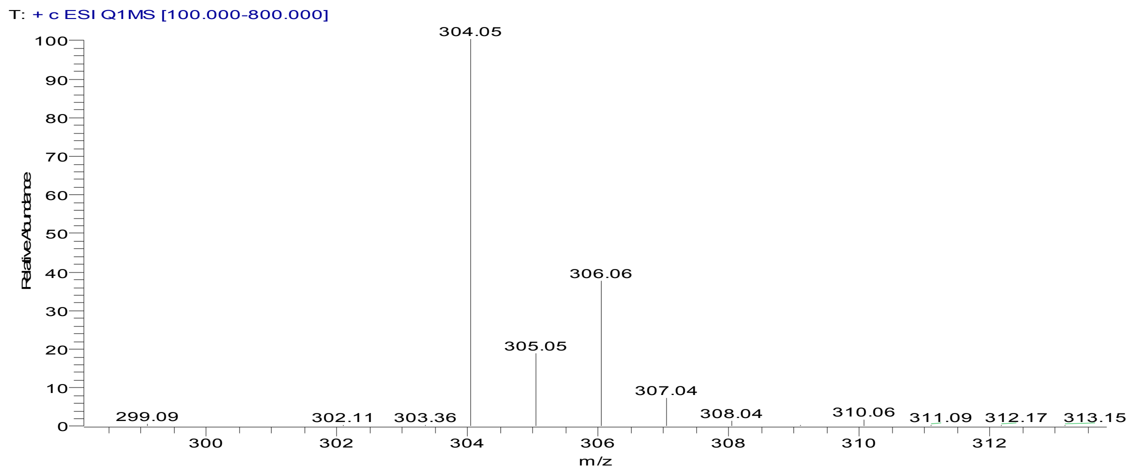

Figure S32. ESI-MS spectrum of compound (Z)-**4c.**

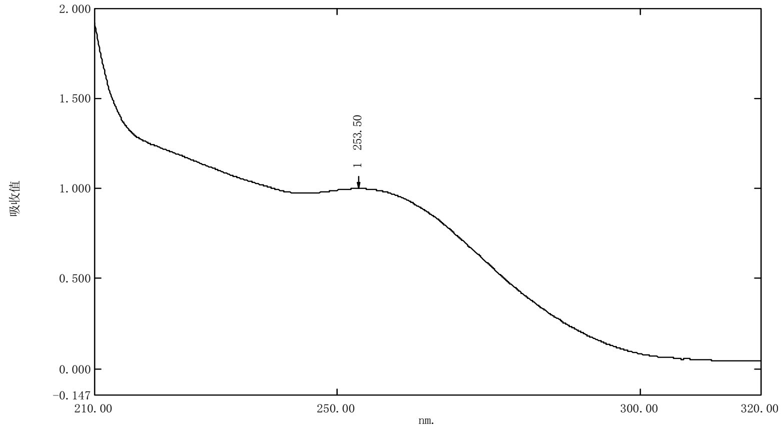

Figure S33. UV-vis spectrum of compound (Z)-**4d** in EtOH.

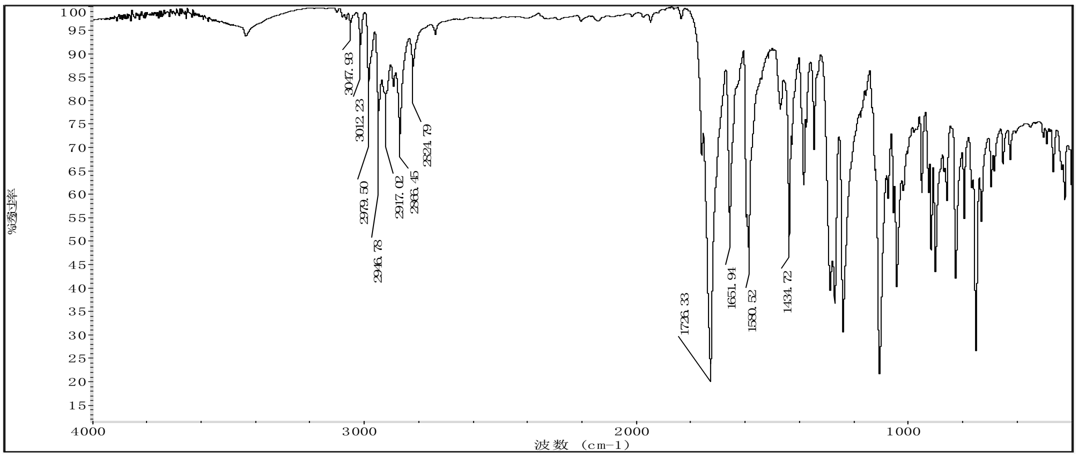

Figure S34. FTIR spectrum of compound (Z)-**4d**.

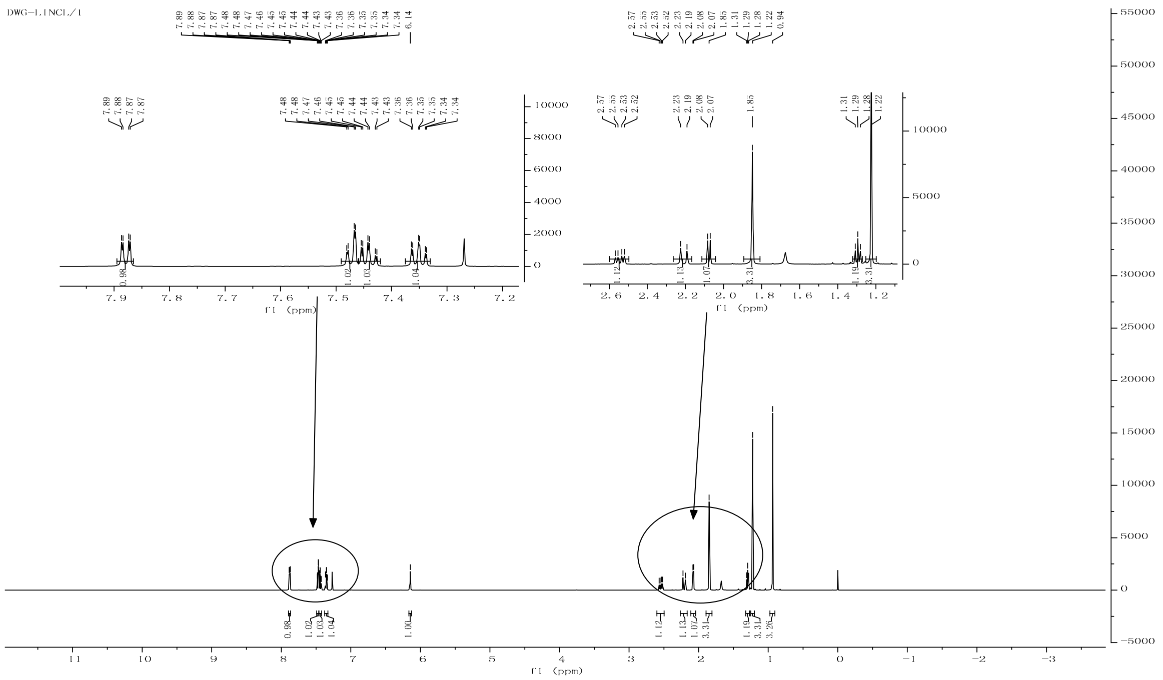

Figure S35. ^{1}H-NMR spectrum of compound (*Z*)-**4d** in $CDCl_3$.

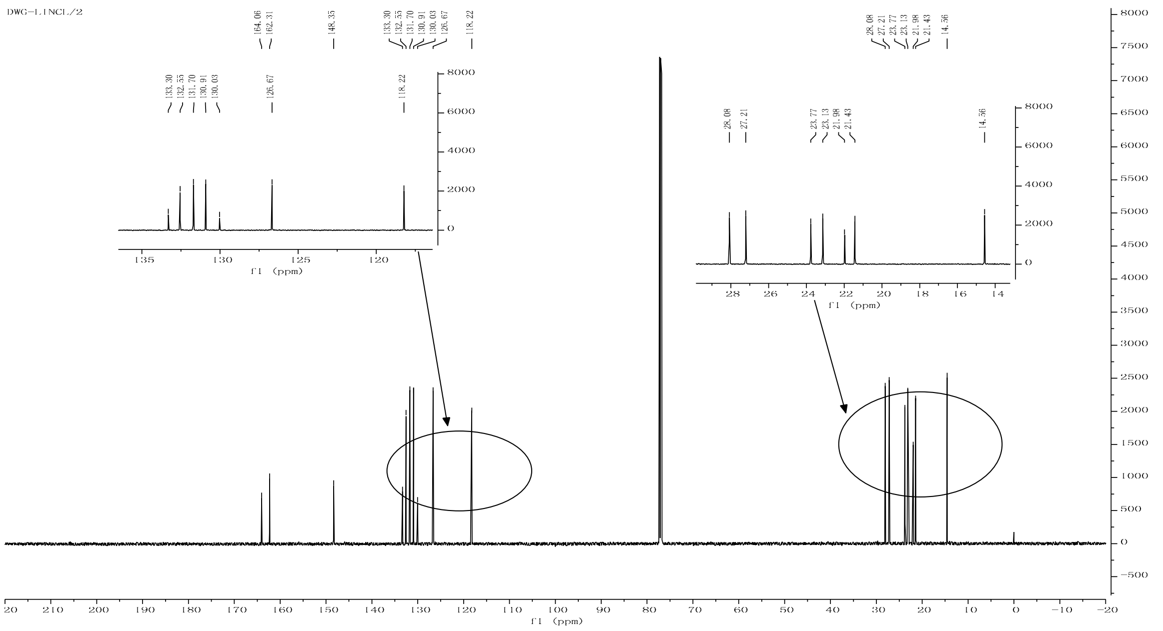

Figure S36. ^{13}C-NMR spectrum of compound (*Z*)-**4d** in $CDCl_3$.

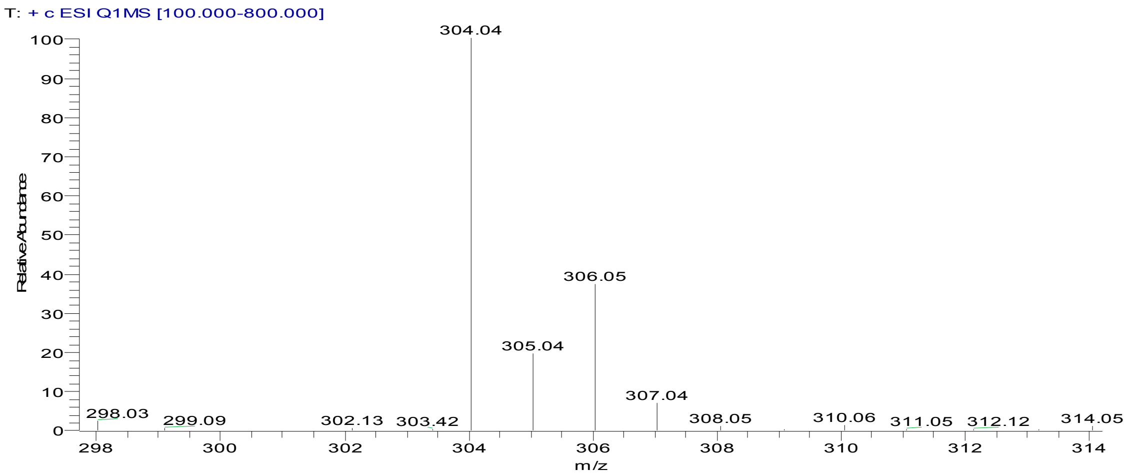

Figure S37. ESI-MS spectrum of compound (*Z*)-**4d.**

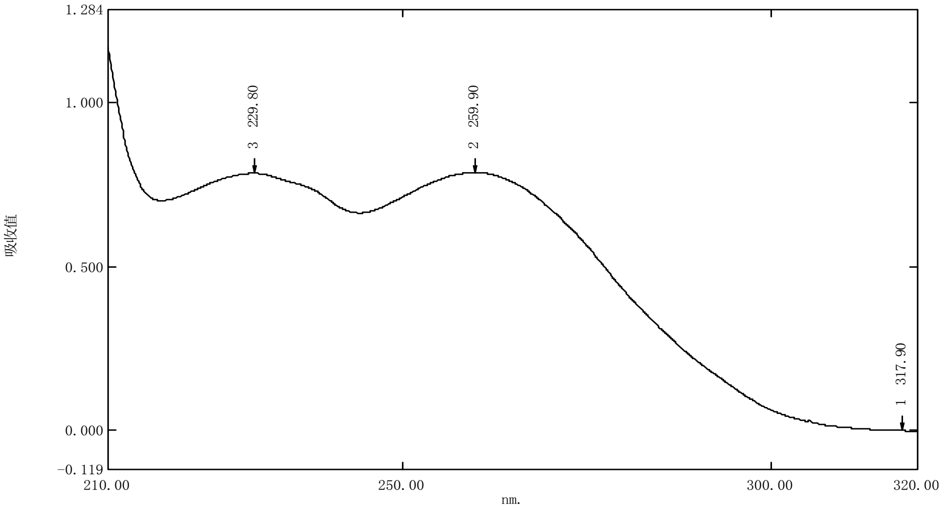

Figure S38. UV-vis spectrum of compound (*Z*)-**4e** in EtOH.

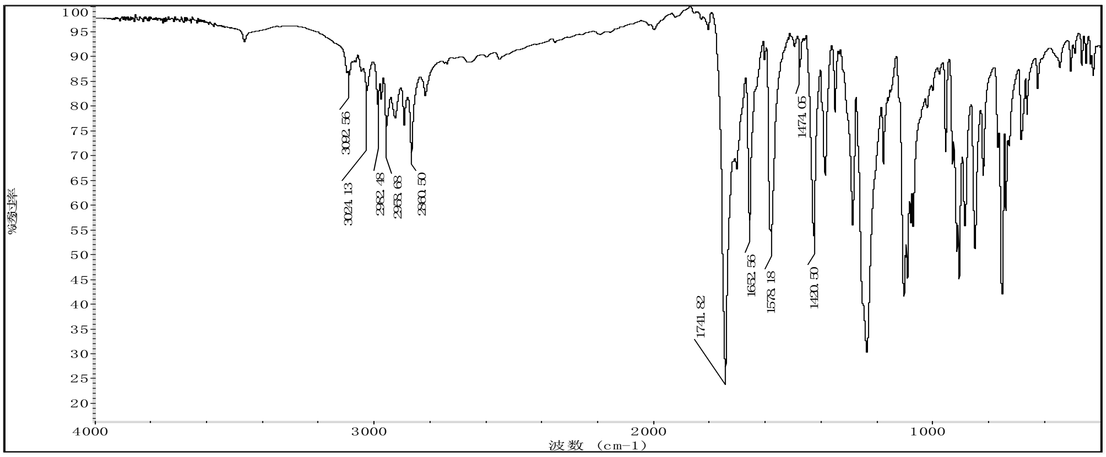

Figure S39. FTIR spectrum of compound (*Z*)-**4e**.

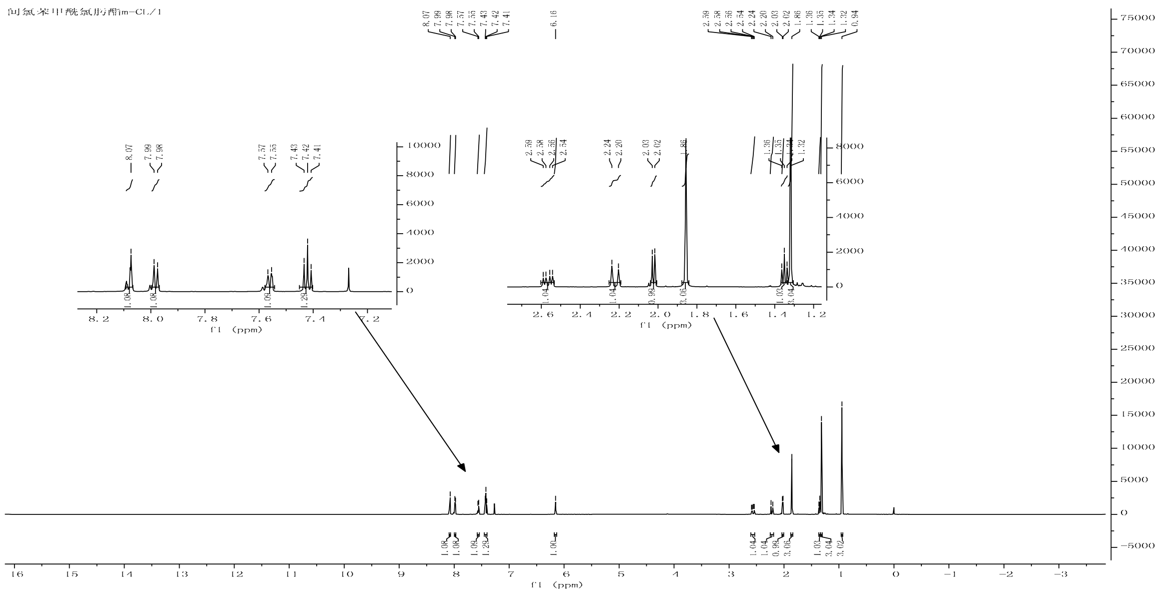

Figure S40. ^{1}H-NMR spectrum of compound (*Z*)-**4e** in $CDCl_3$.

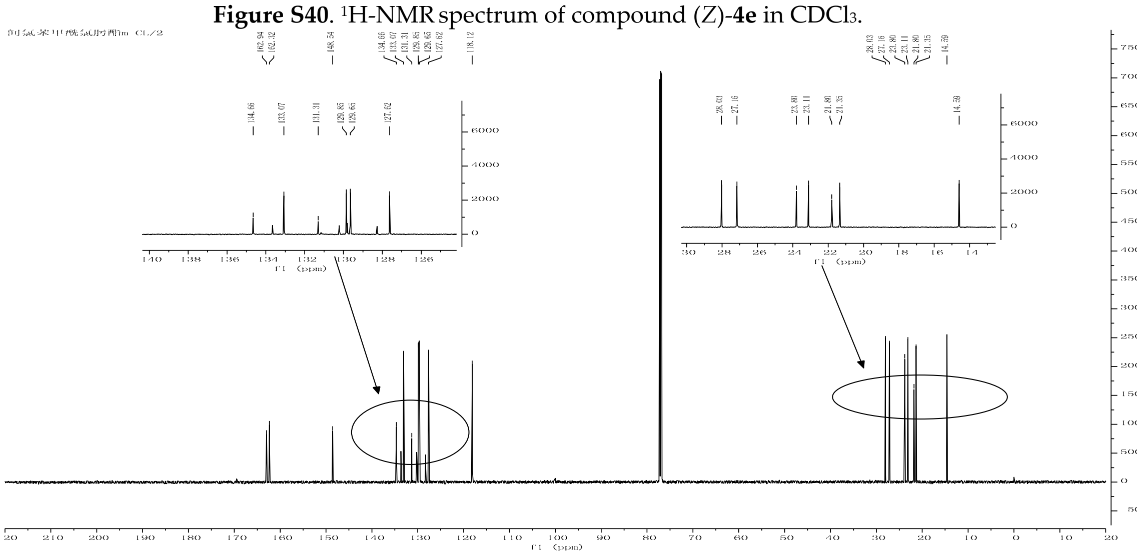

Figure S41. ^{13}C-NMR spectrum of compound (*Z*)-**4e** in $CDCl_3$.

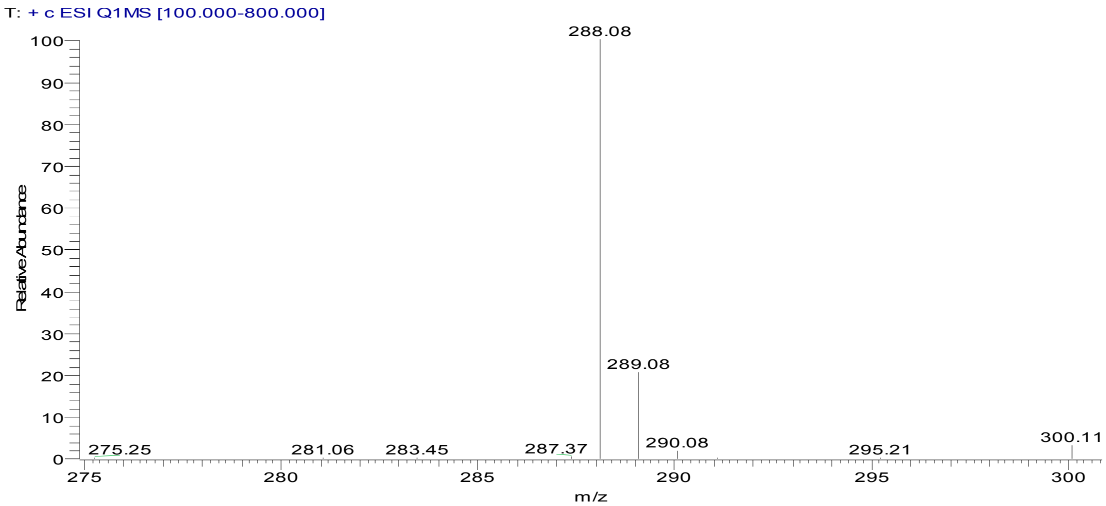

Figure S42. ESI-MS spectrum of compound (*Z*)-**4e.**

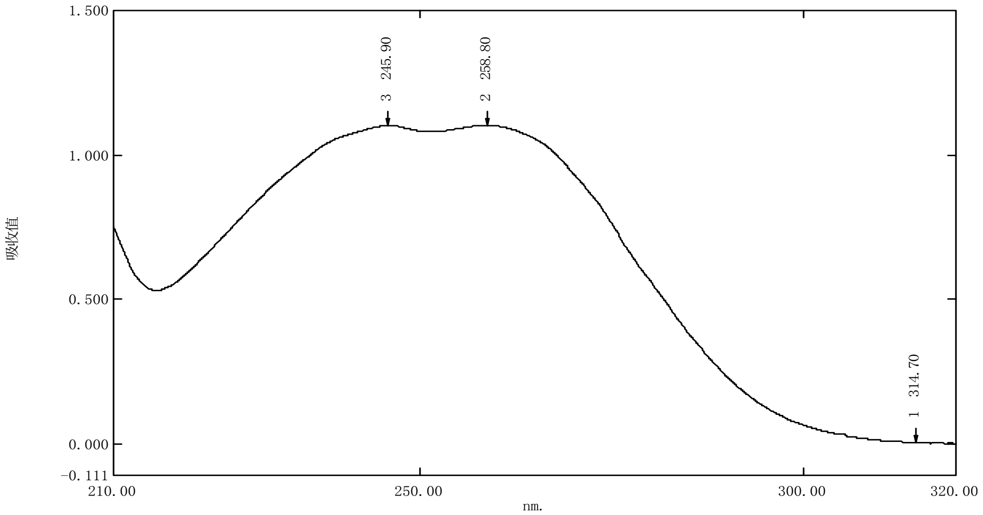

Figure S43. UV-vis spectrum of compound (*Z*)-**4f** in EtOH.

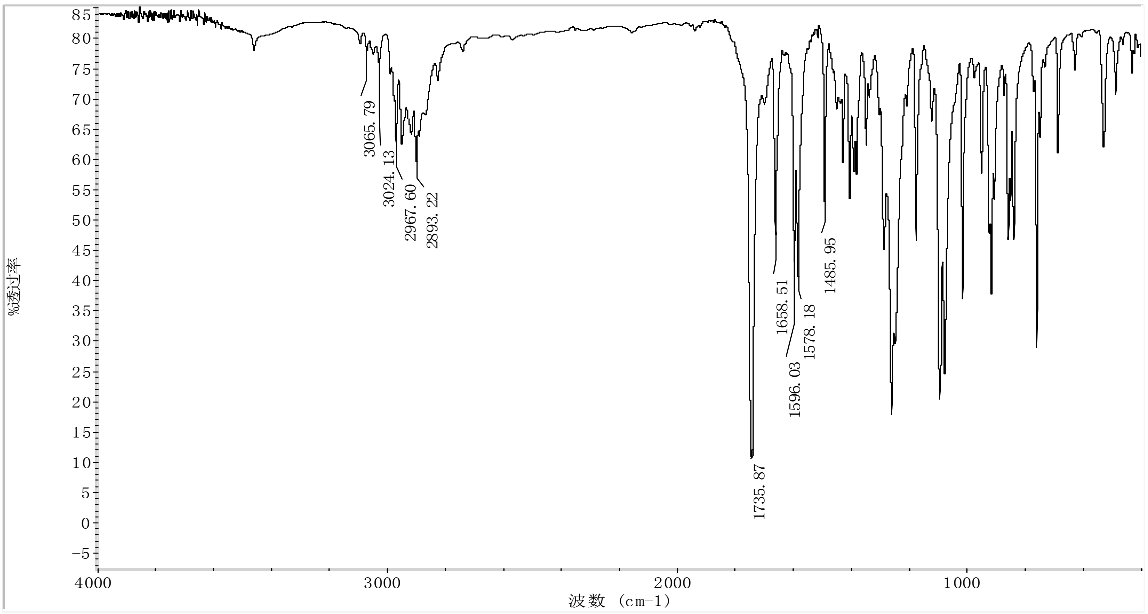

Figure S44. FTIR spectrum of compound (*Z*)-**4f**.

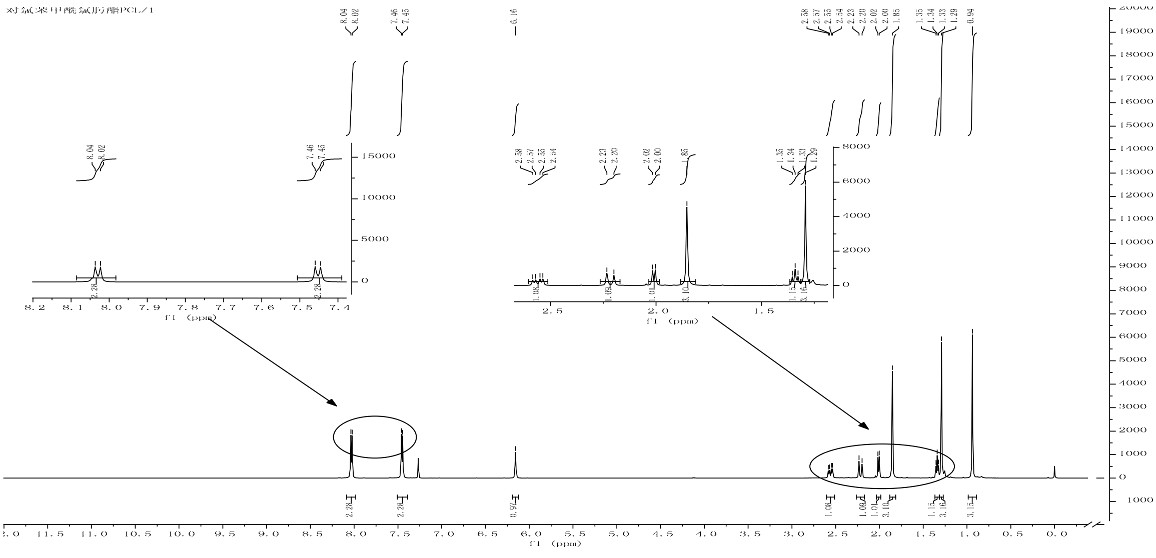

Figure S45. ^{1}H-NMR spectrum of compound (*Z*)-**4f** in $CDCl_3$.

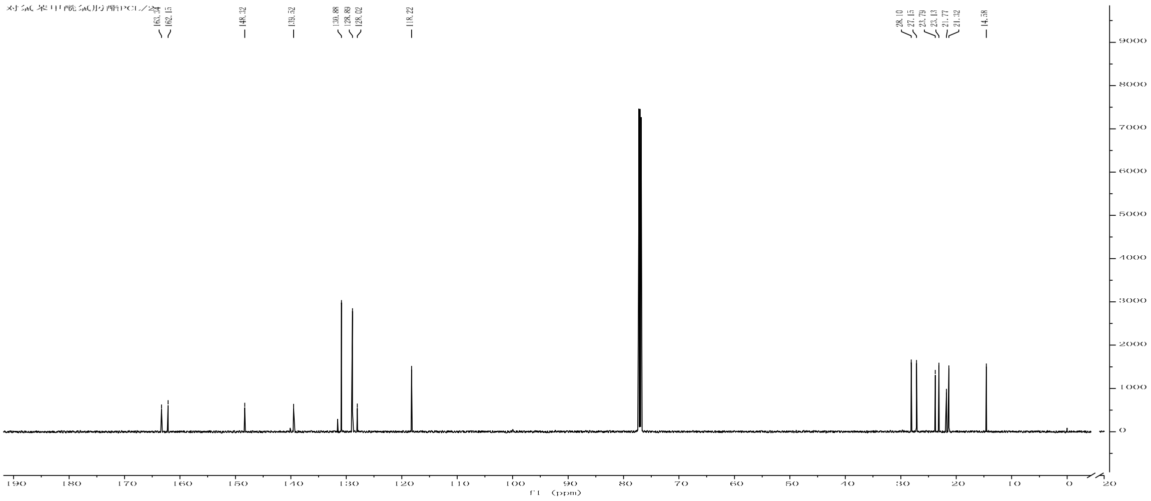

Figure S46. ^{13}C-NMR spectrum of compound (*Z*)-**4f** in $CDCl_3$.

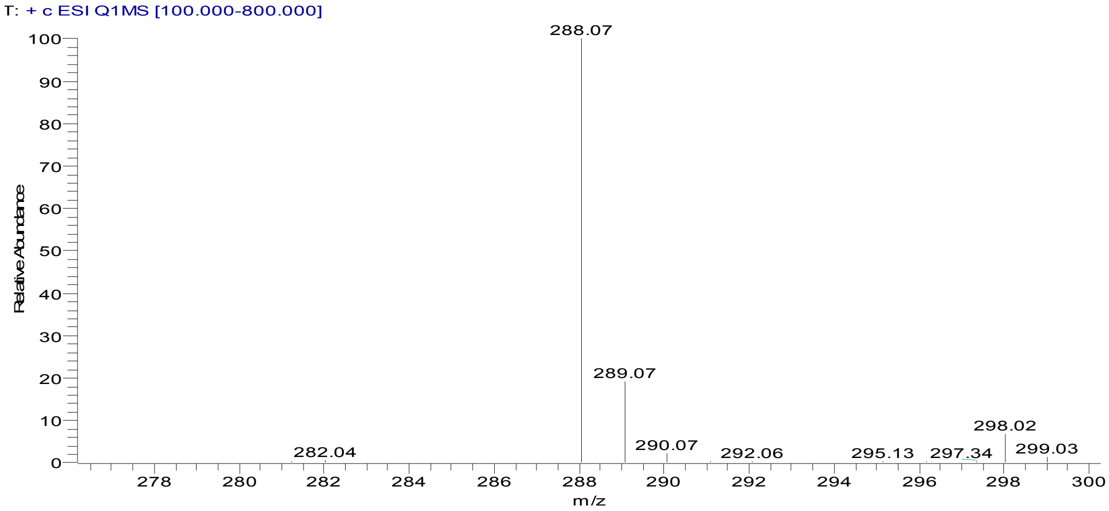

Figure S47. ESI-MS spectrum of compound (*Z*)-**4f.**

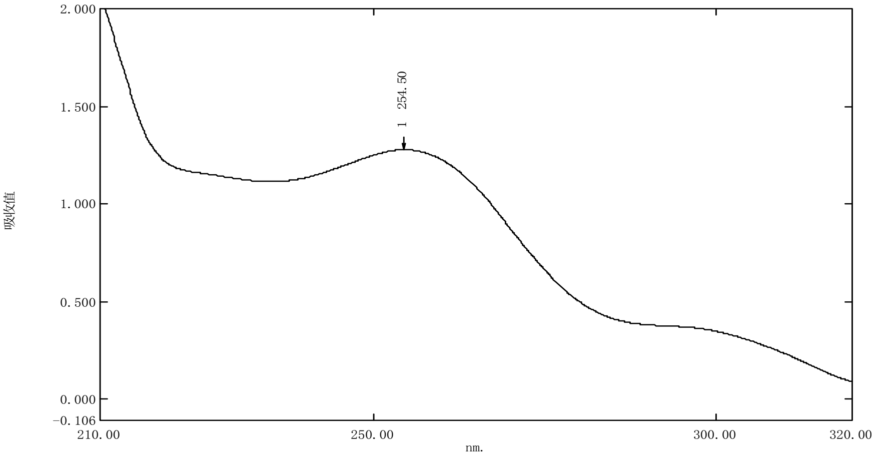

Figure S48 UV-vis spectrum of compound (*Z*)-**4g** in EtOH.

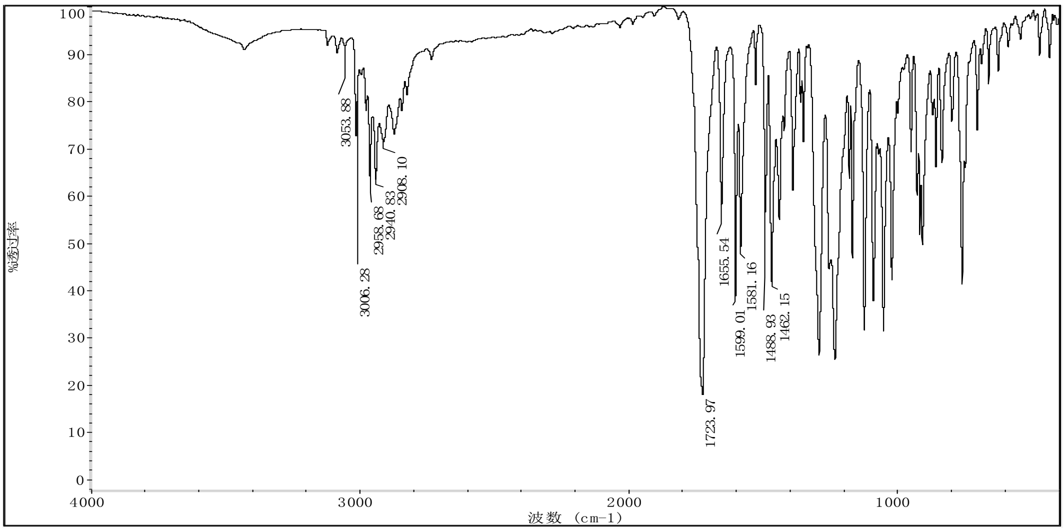

Figure S49. FTIR spectrum of compound (*Z*)-**4g**.

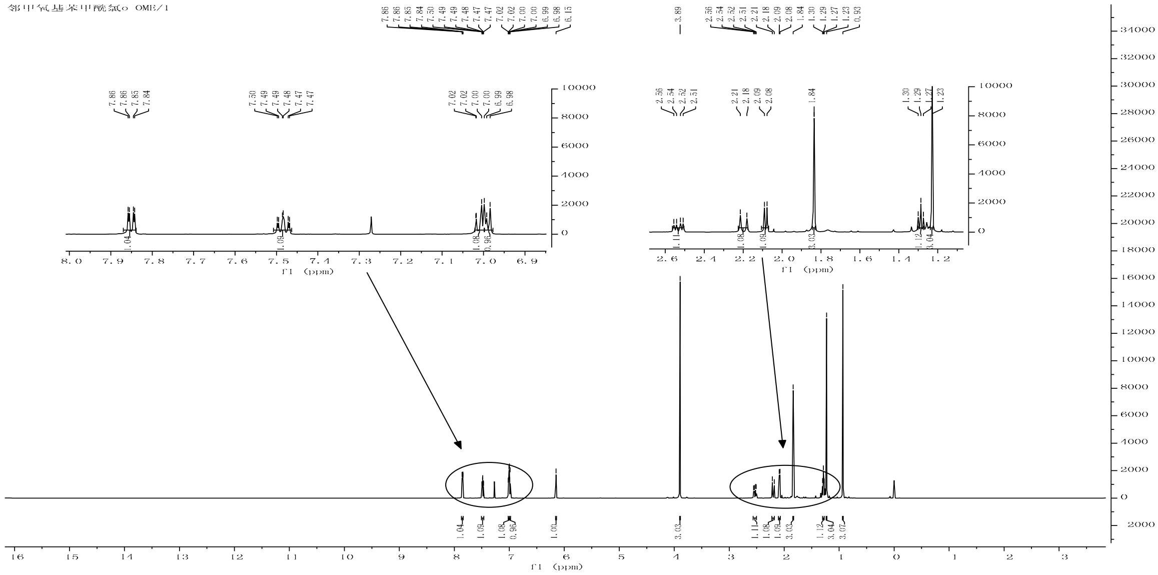

Figure S50. ^{1}H-NMR spectrum of compound (*Z*)-**4g** in $CDCl_3$

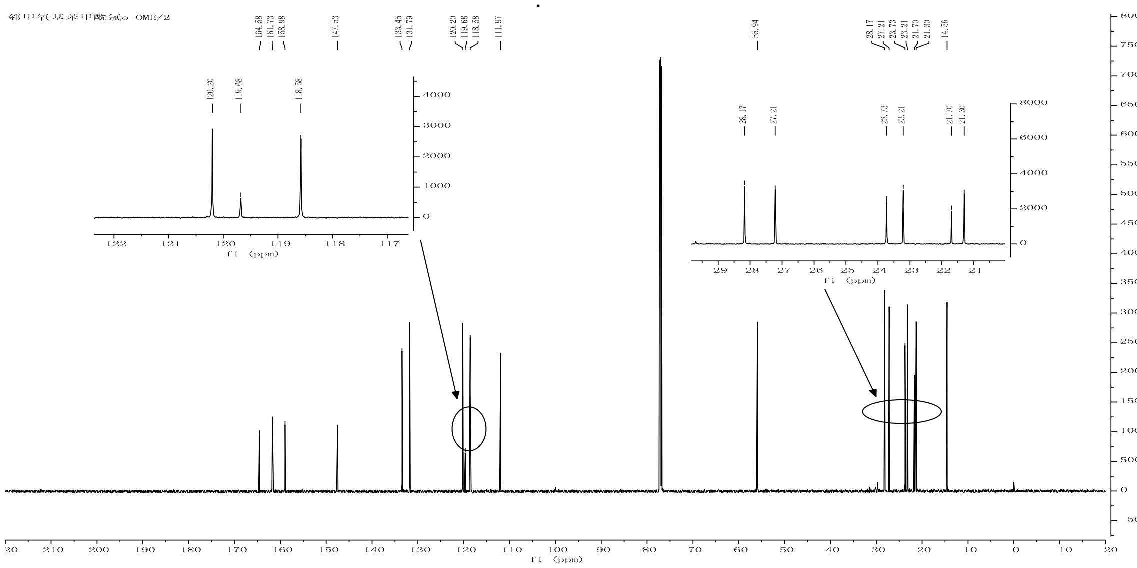

Figure S51. ^{13}C-NMR spectrum of compound (*Z*)-**4g** in $CDCl_3$.

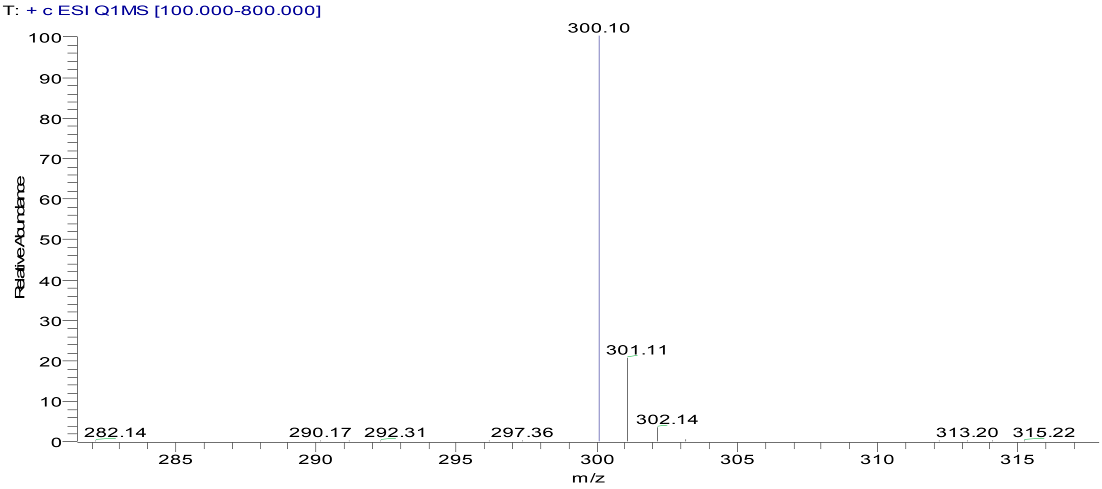

Figure S52. ESI-MS spectrum of compound (*Z*)-**4g.**

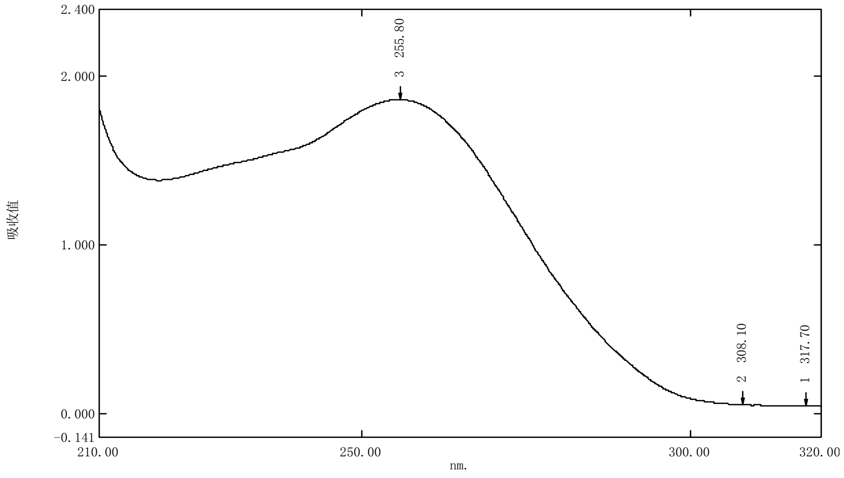

Figure S53. UV-vis spectrum of compound (*Z*)-**4h** in EtOH.

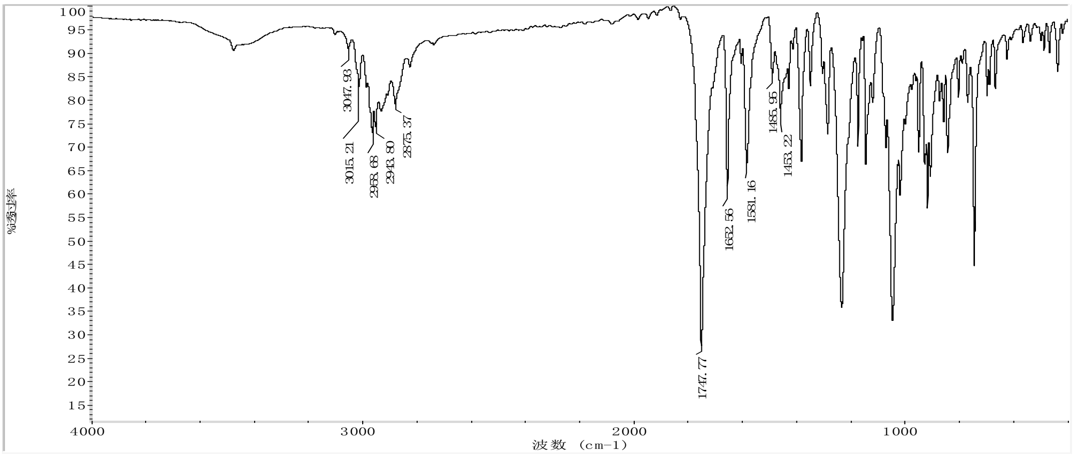

Figure S54. FTIR spectrum of compound (*Z*)-**4h**.

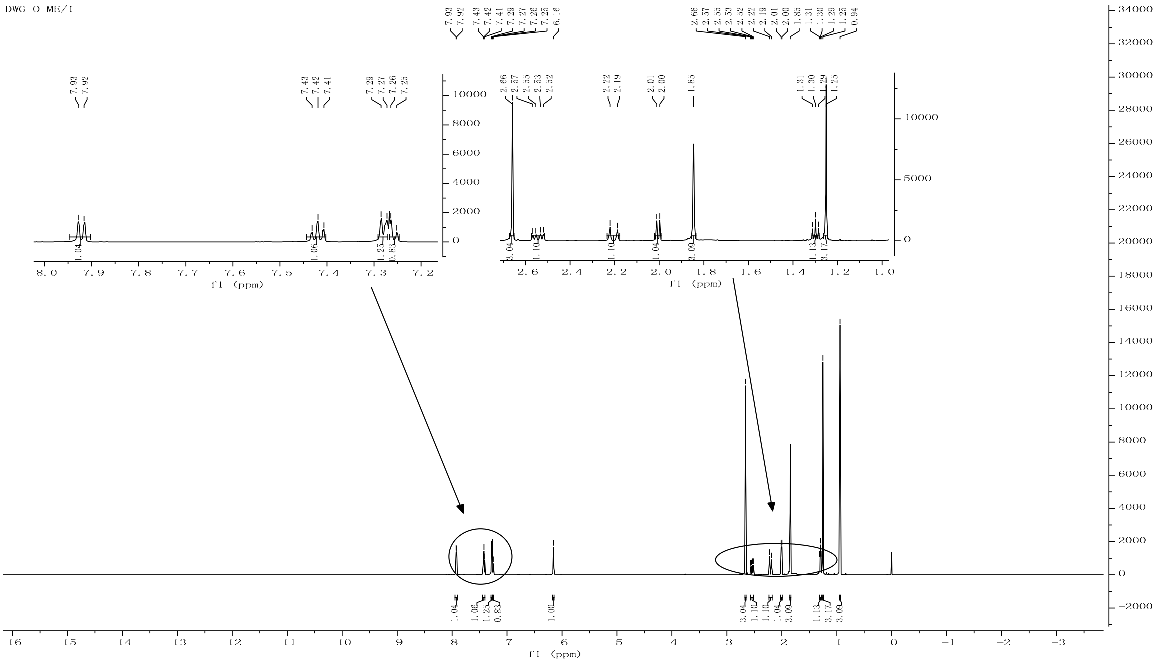

Figure S55. ^{1}H-NMR spectrum of compound (*Z*)-**4h** in $CDCl_3$.

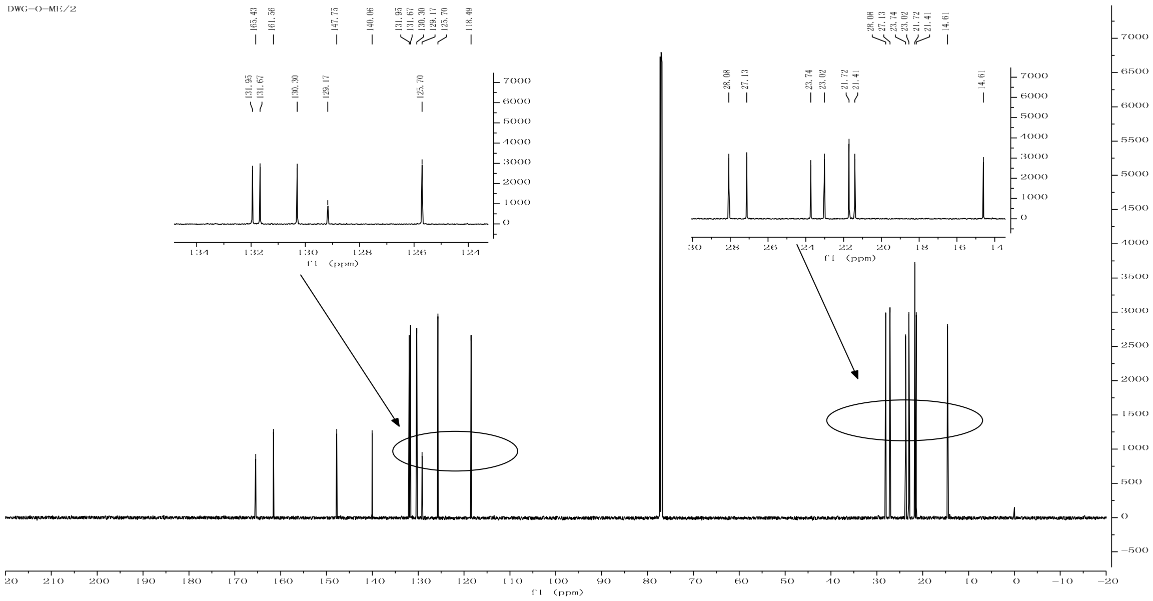

Figure S56. ^{13}C-NMR spectrum of compound (*Z*)-**4h** in $CDCl_3$.

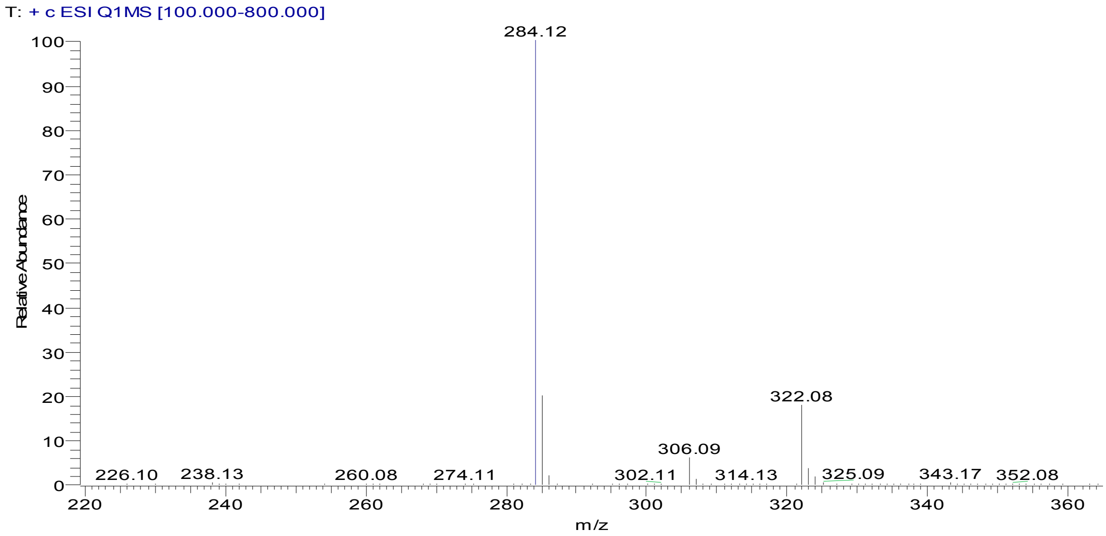

Figure S57. ESI-MS spectrum of compound (*Z*)-**4h.**

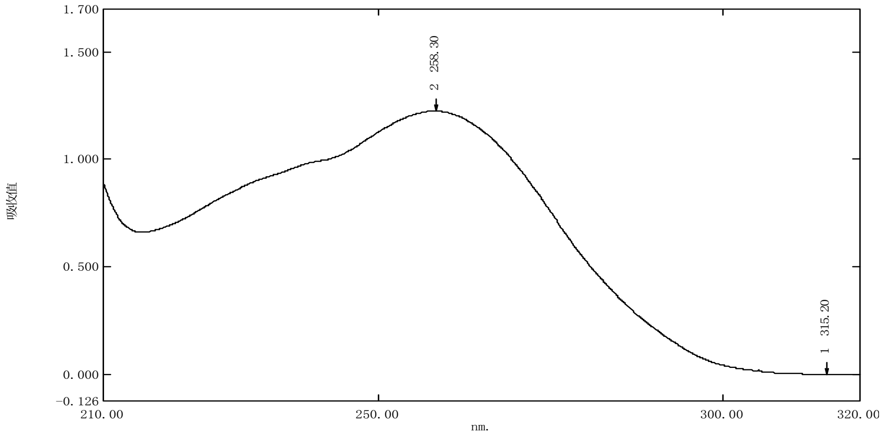

Figure S58. UV-vis spectrum of compound (*Z*)-**4i** in EtOH.

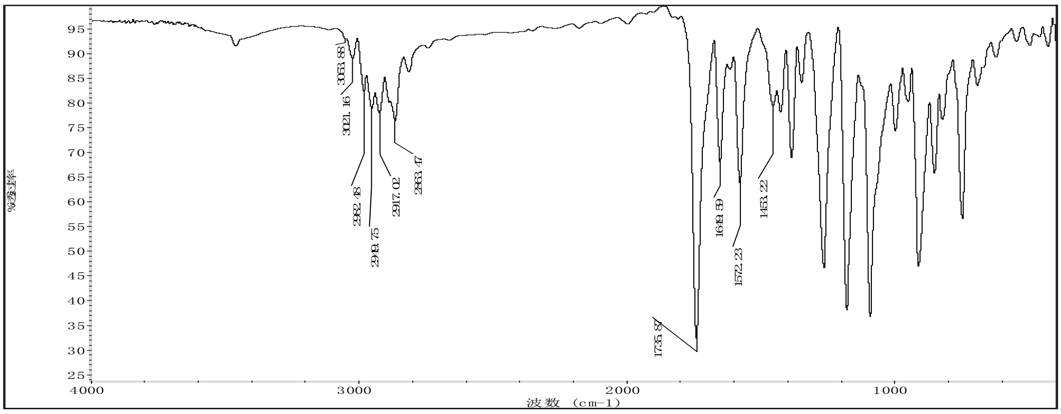

Figure S59. FTIR spectrum of compound (*Z*)-**4i**.

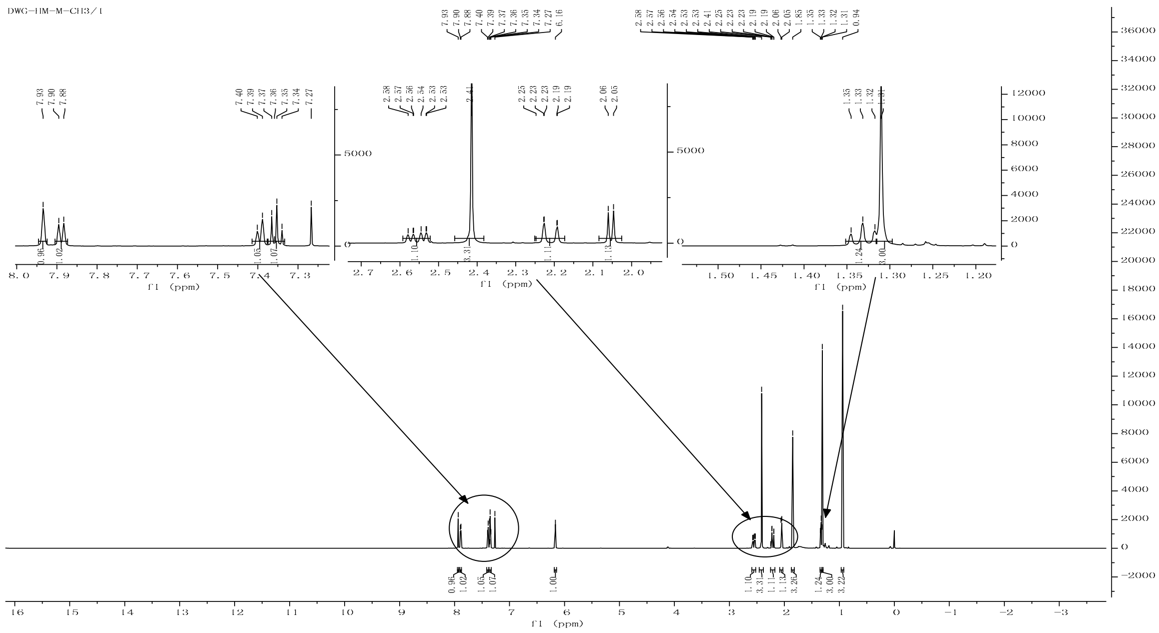

Figure S60. ^{1}H-NMR spectrum of compound (*Z*)-**4i** in $CDCl_3$.

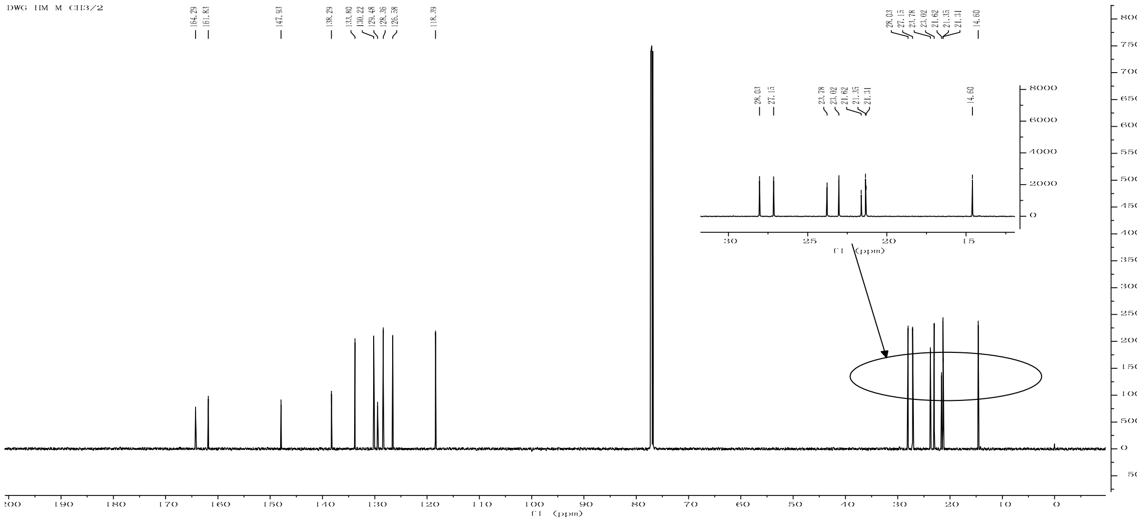

Figure S61. ^{13}C-NMR spectrum of compound (*Z*)-**4i** in $CDCl_3$.

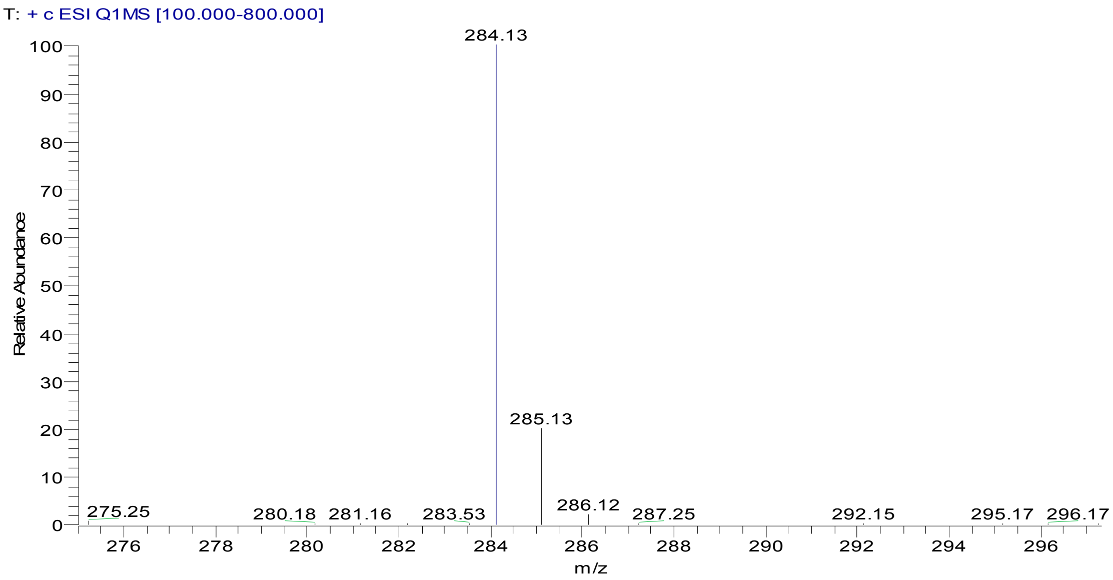

Figure S62. ESI-MS spectrum of compound (*Z*)-**4i.**

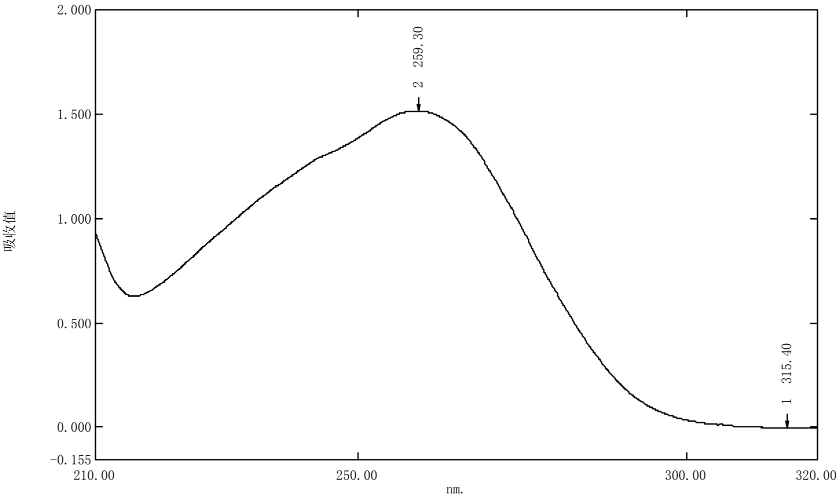

Figure S63. UV-vis spectrum of compound (*Z*)-**4j** in EtOH.

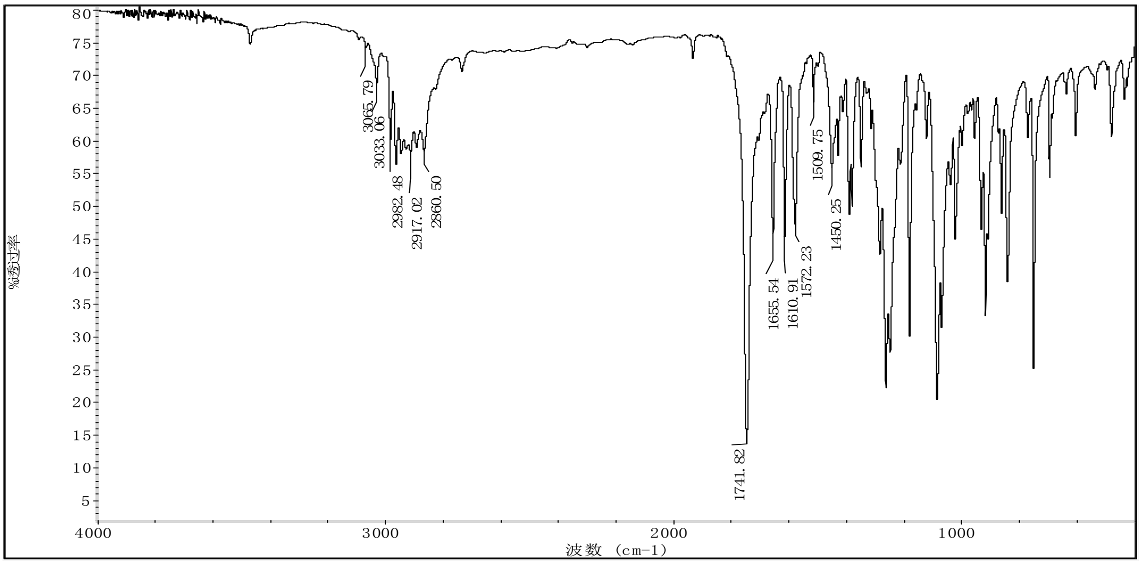

Figure S64. FTIR spectrum of compound (*Z*)-**4j**.

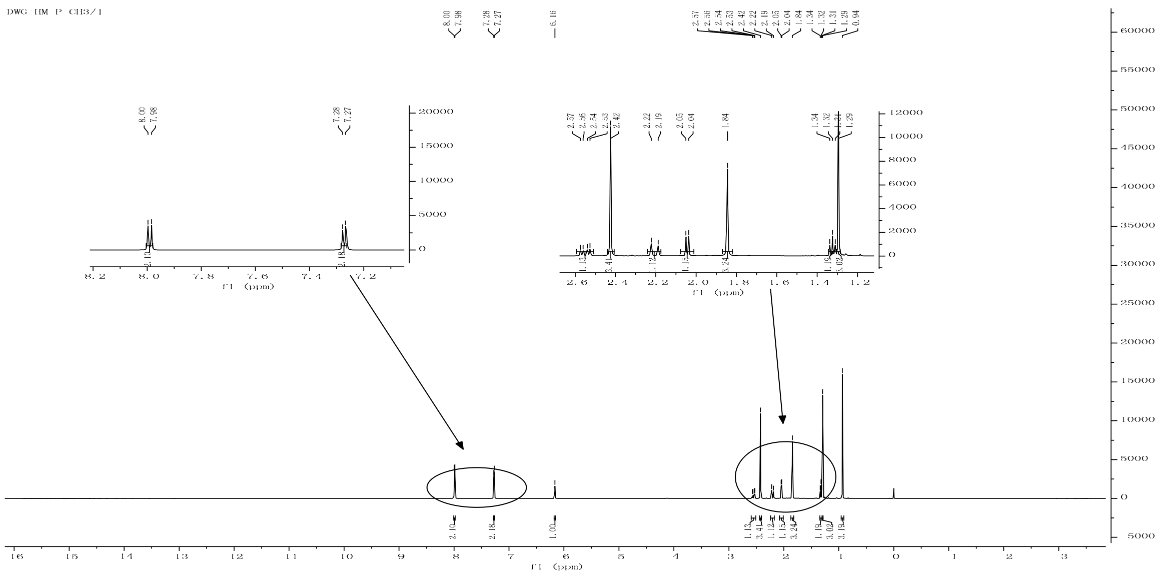

Figure S65. ^{1}H-NMR spectrum of compound (*Z*)-**4j** in $CDCl_3$.

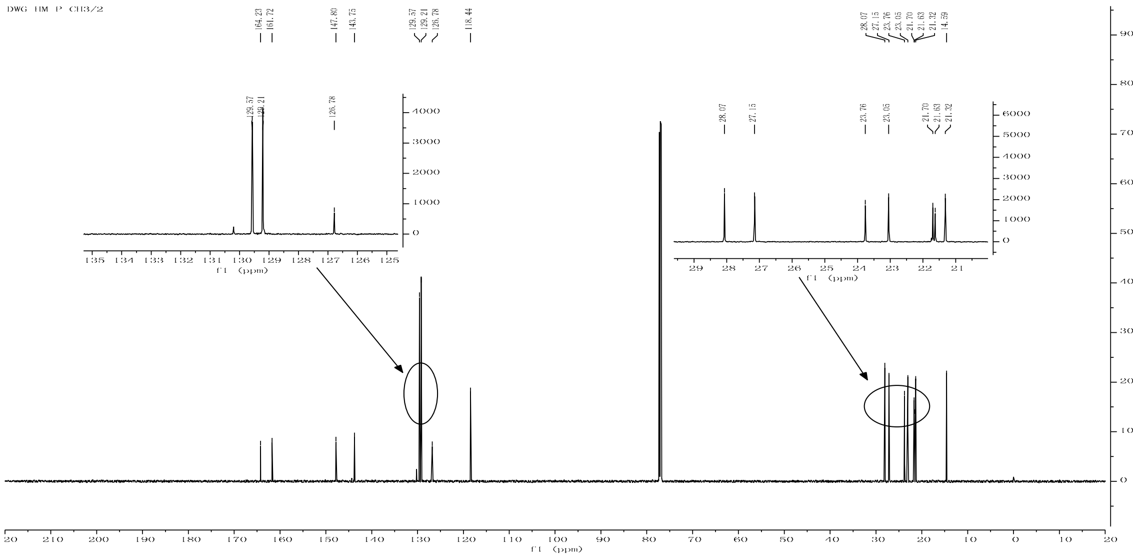

Figure S66. ^{13}C-NMR spectrum of compound (*Z*)-**4j** in $CDCl_3$.

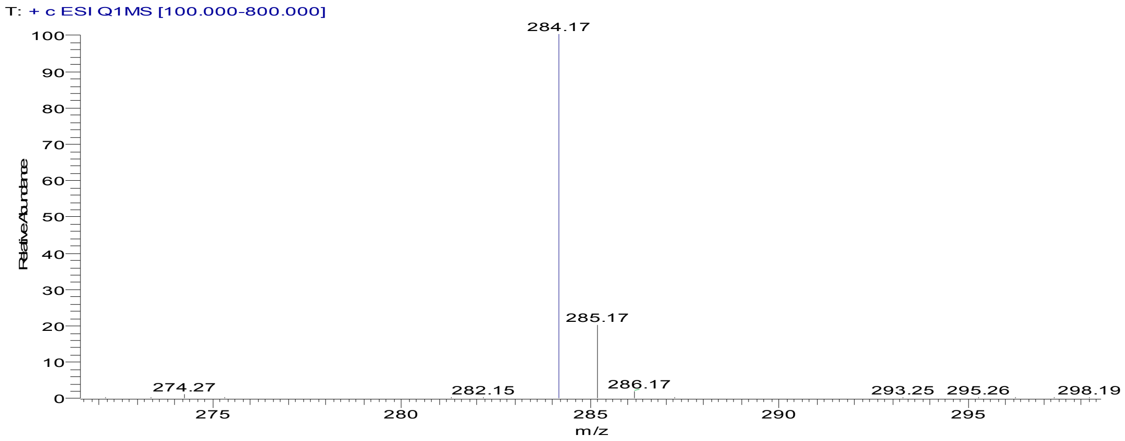

Figure S67. ESI-MS spectrum of compound (*Z*)-**4j**

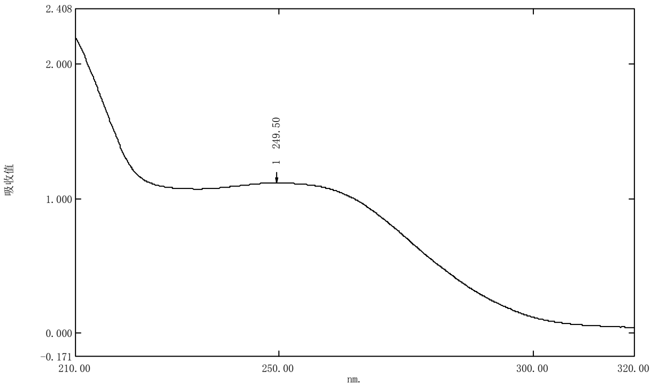

Figure S68 UV-vis spectrum of compound (*Z*)-**4k** in EtOH.

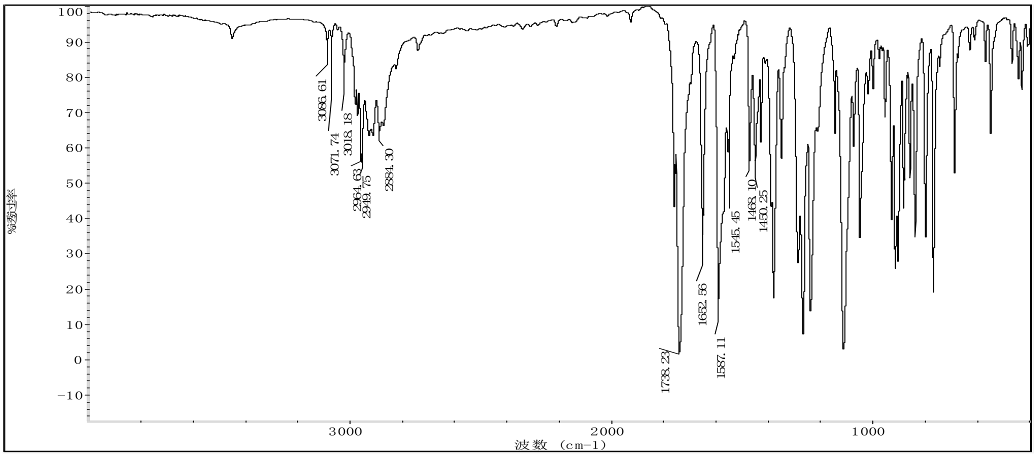

Figure S69. FTIR spectrum of compound (*Z*)-**4k**.

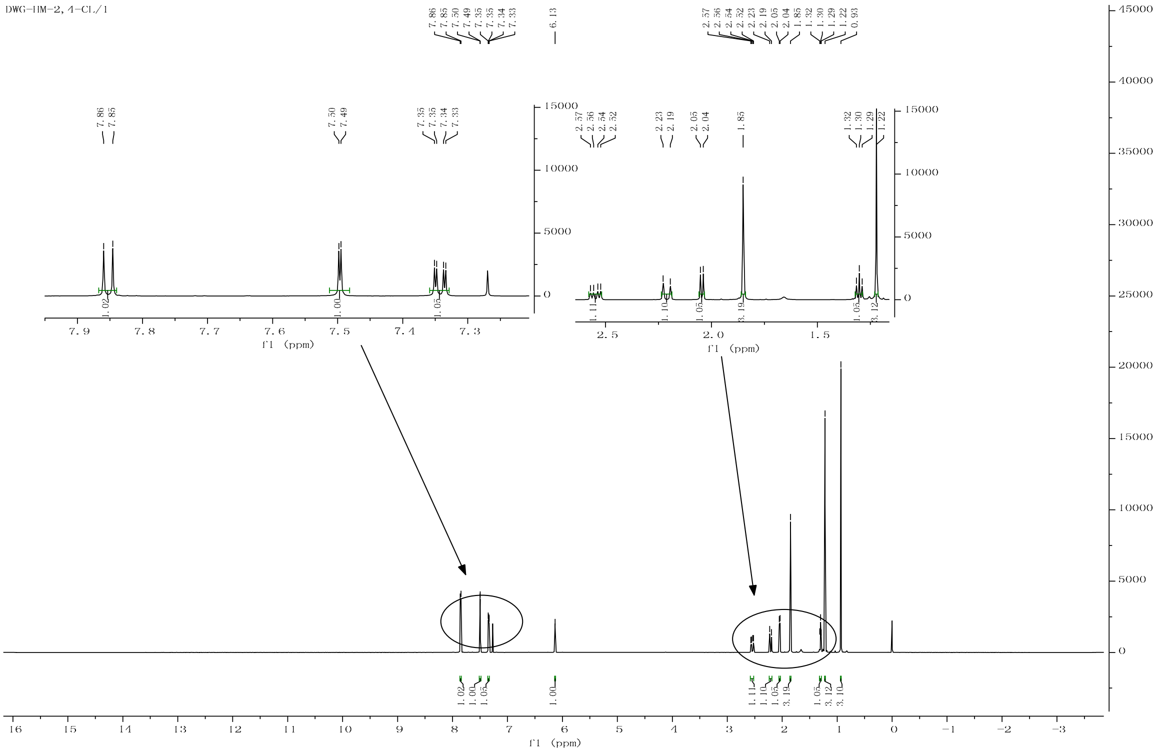

Figure S70. ^{1}H-NMR spectrum of compound (*Z*)-**4k** in $CDCl_3$.

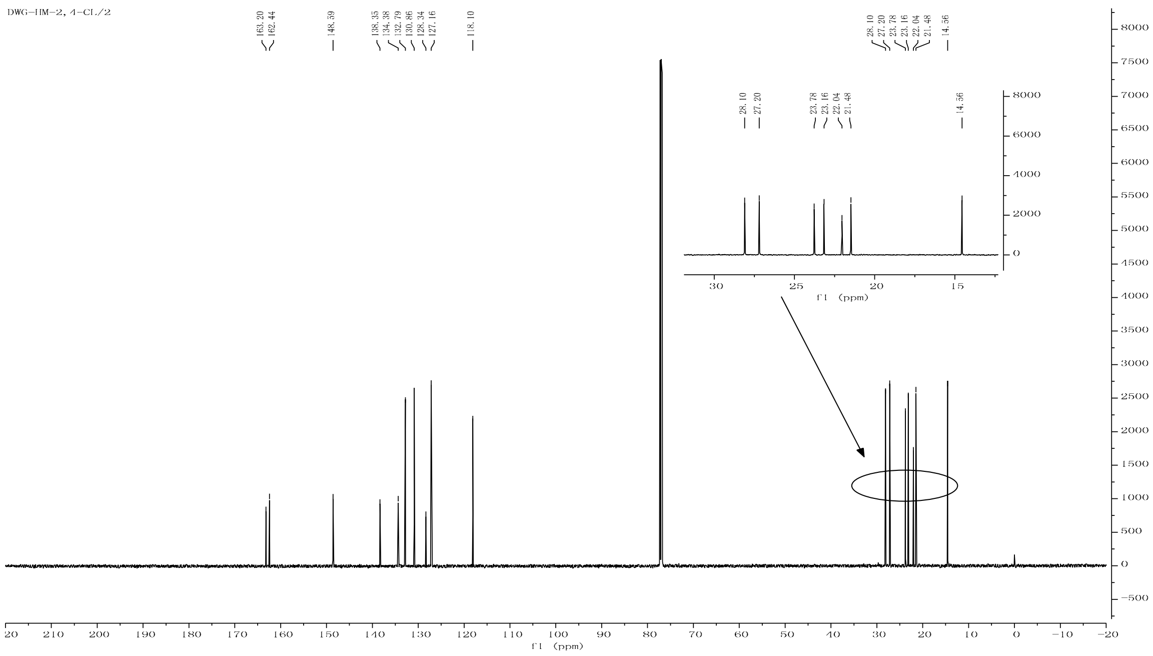

Figure S71. ^{13}C-NMR spectrum of compound (*Z*)-**4k** in $CDCl_3$.

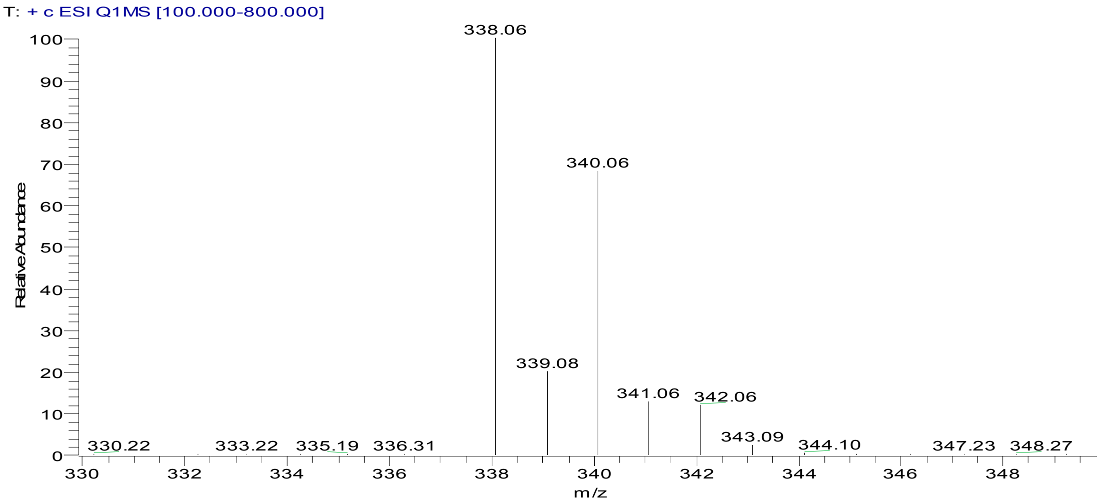

Figure S72. ESI-MS spectrum of compound (*Z*)-**4k.**

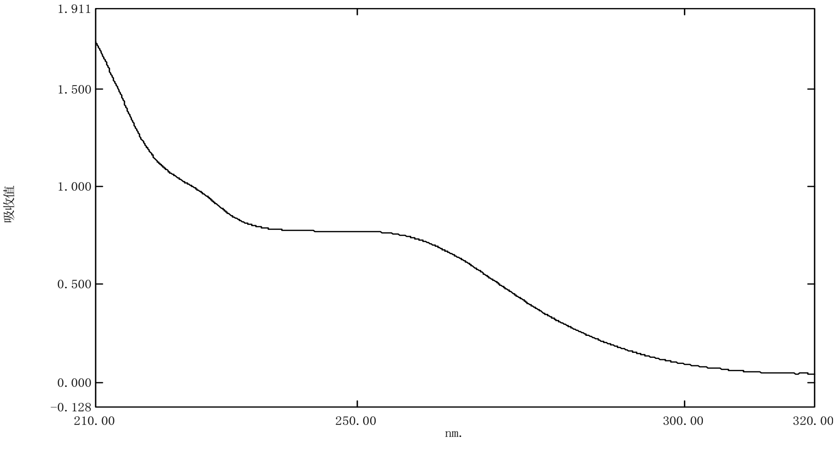

Figure S73. UV-vis spectrum of compound (*Z*)-**4l** in EtOH.

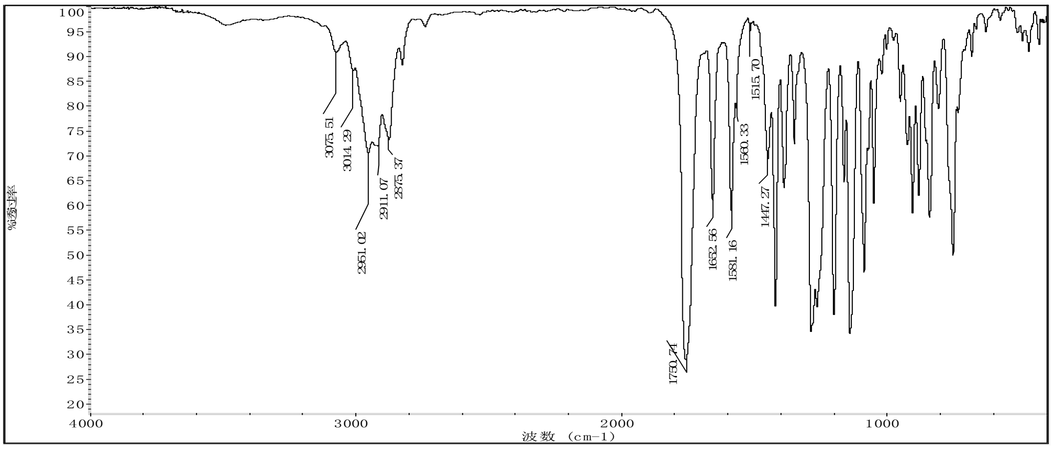

Figure S74. FTIR spectrum of compound (*Z*)-**4l**.

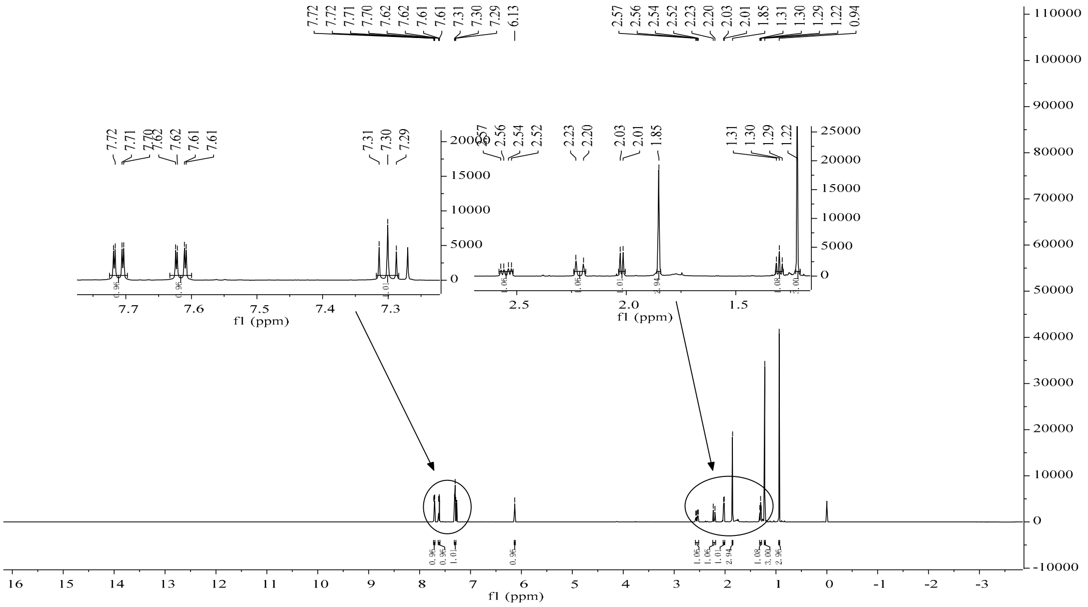

Figure S75. ^{1}H-NMR spectrum of compound (*Z*)-**4l** in $CDCl_3$.

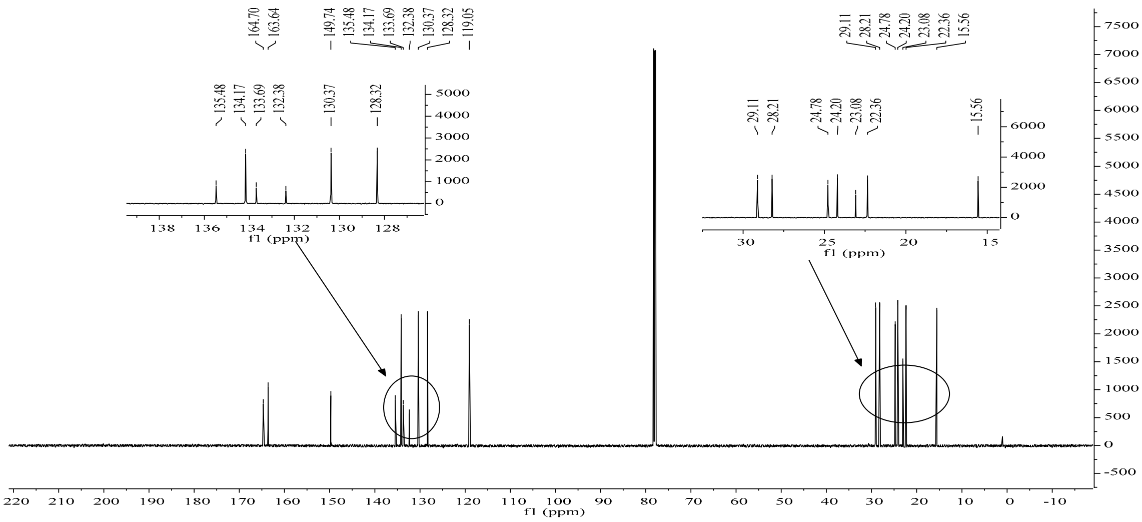

Figure S76. ^{13}C-NMR spectrum of compound(*Z*)-**4l** in $CDCl_3$.

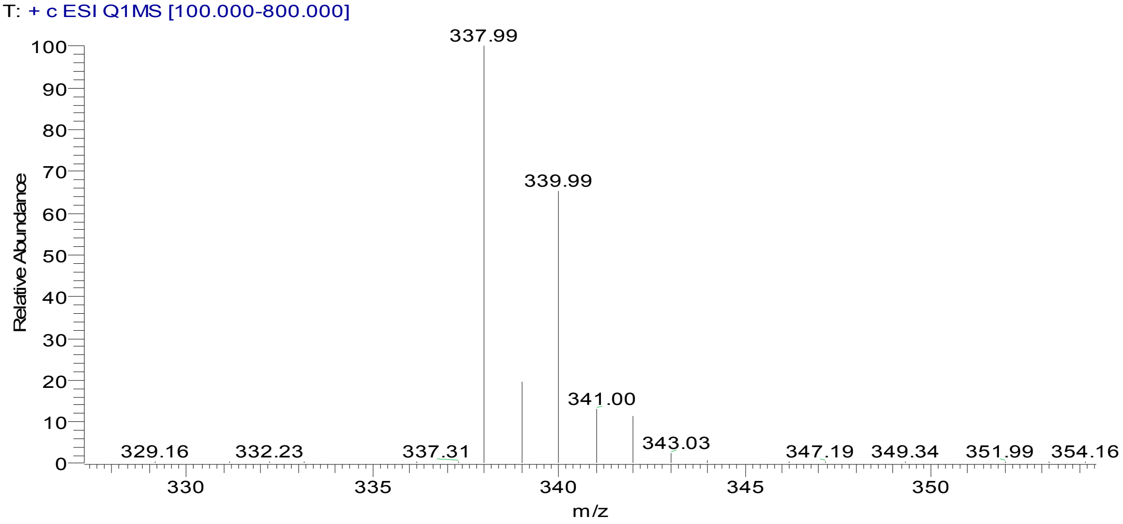

Figure S77. ESI-MS spectrum of compound (Z)-**4l.**

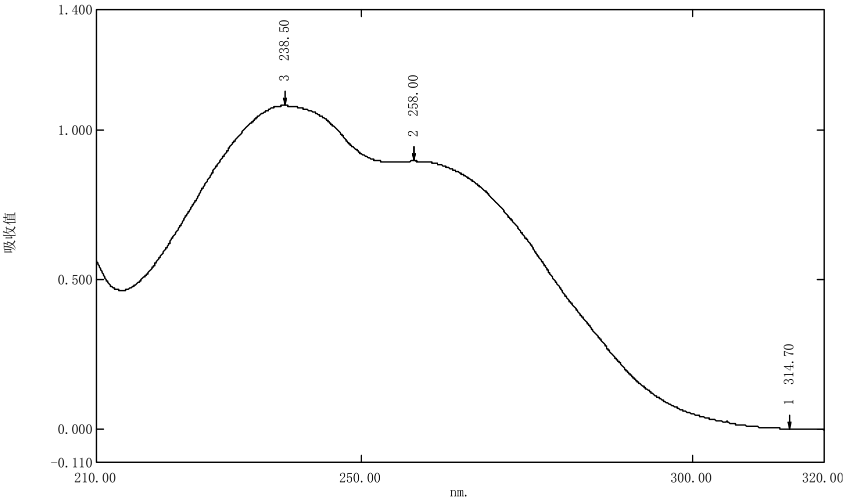

Figure S78. UV-vis spectrum of compound (Z)-**4m** in EtOH.

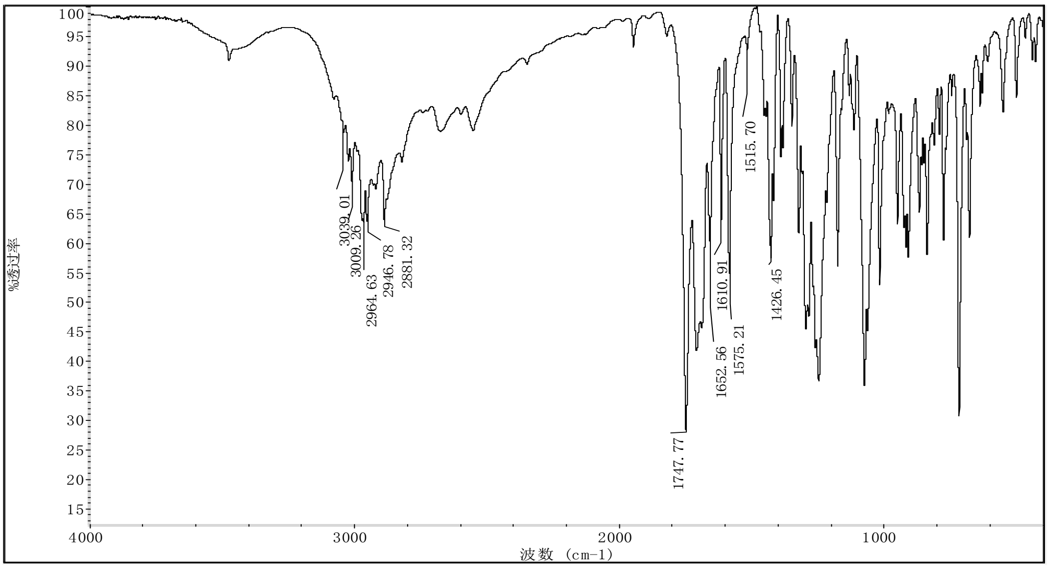

Figure S79. FTIR spectrum of compound (Z)-**4m**.

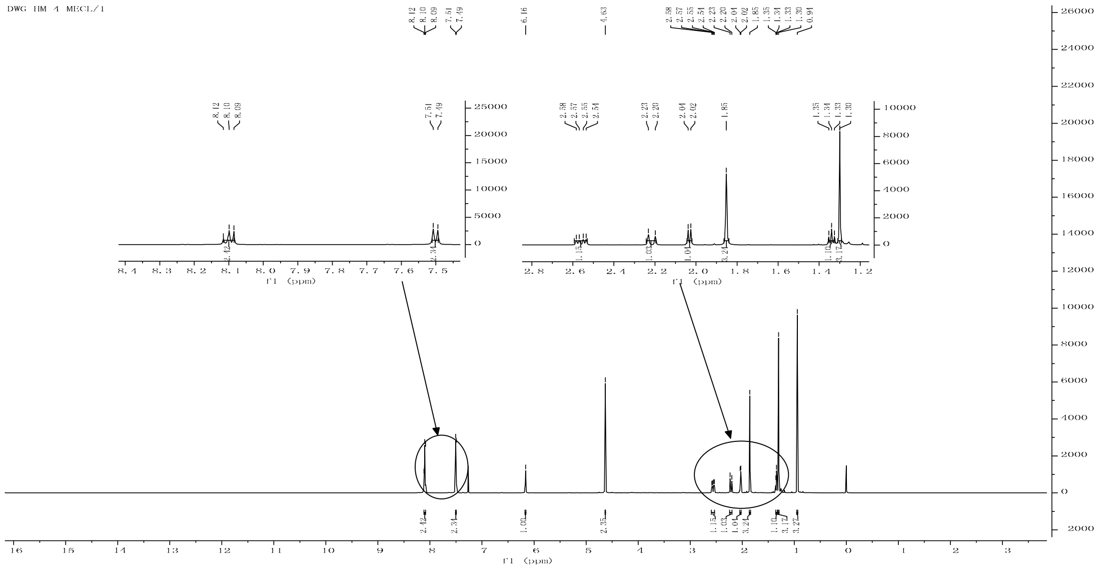

Figure S80. ^{1}H-NMR spectrum of compound (*Z*)-**4m** in CDCl3.

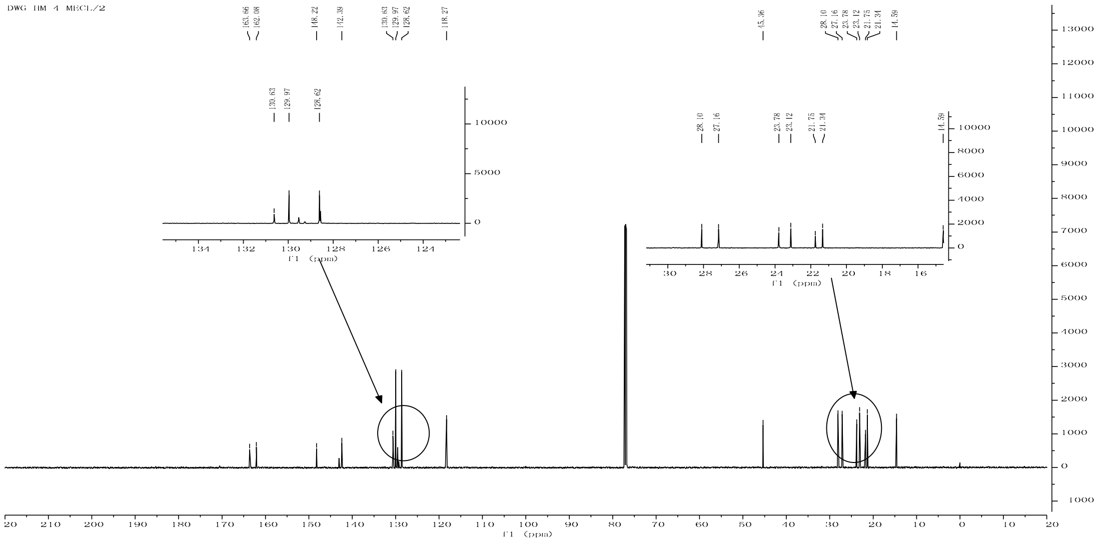

Figure S81. ^{13}C-NMR spectrum of compound (*Z*)-**4m** in CDCl3.

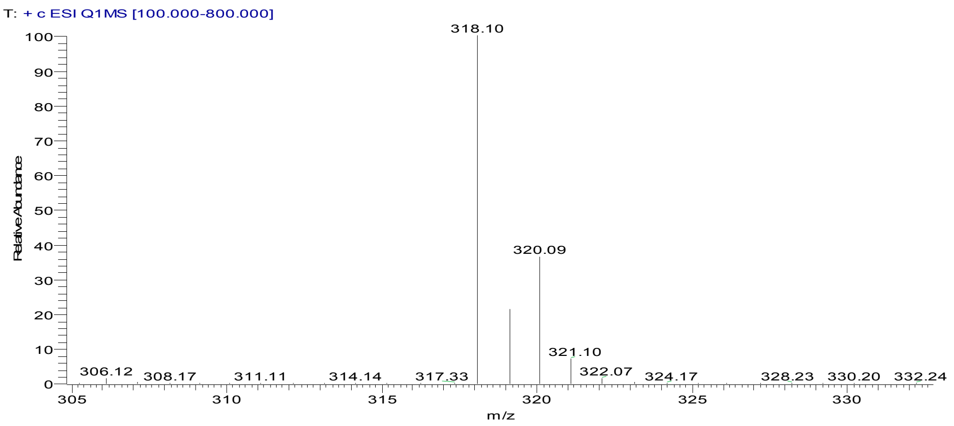

Figure S82. ESI-MS spectrum of compound (*Z*)-**4m.**

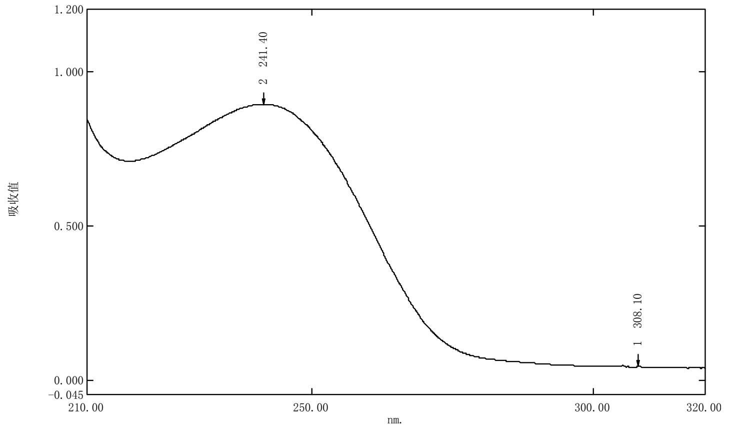

Figure S83. UV-vis spectrum of compound (*Z*)-**4n** in EtOH.

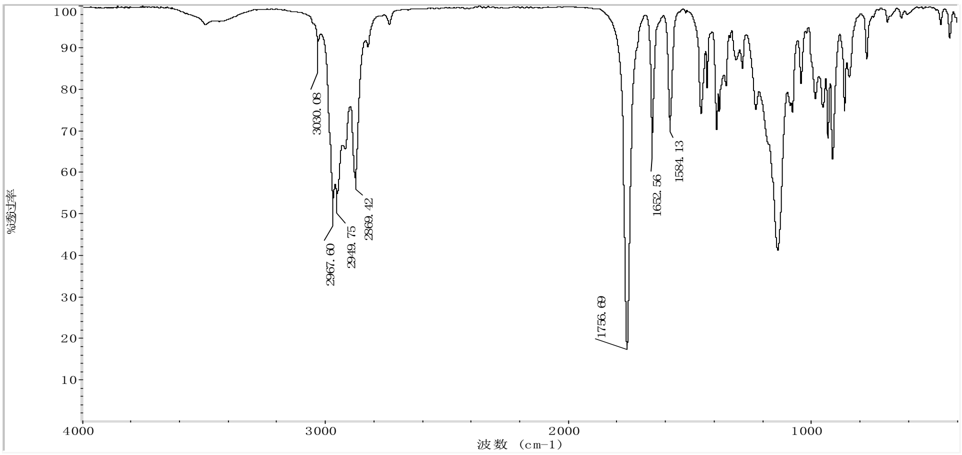

Figure S84. FTIR spectrum of compound (*Z*)-**4n**.

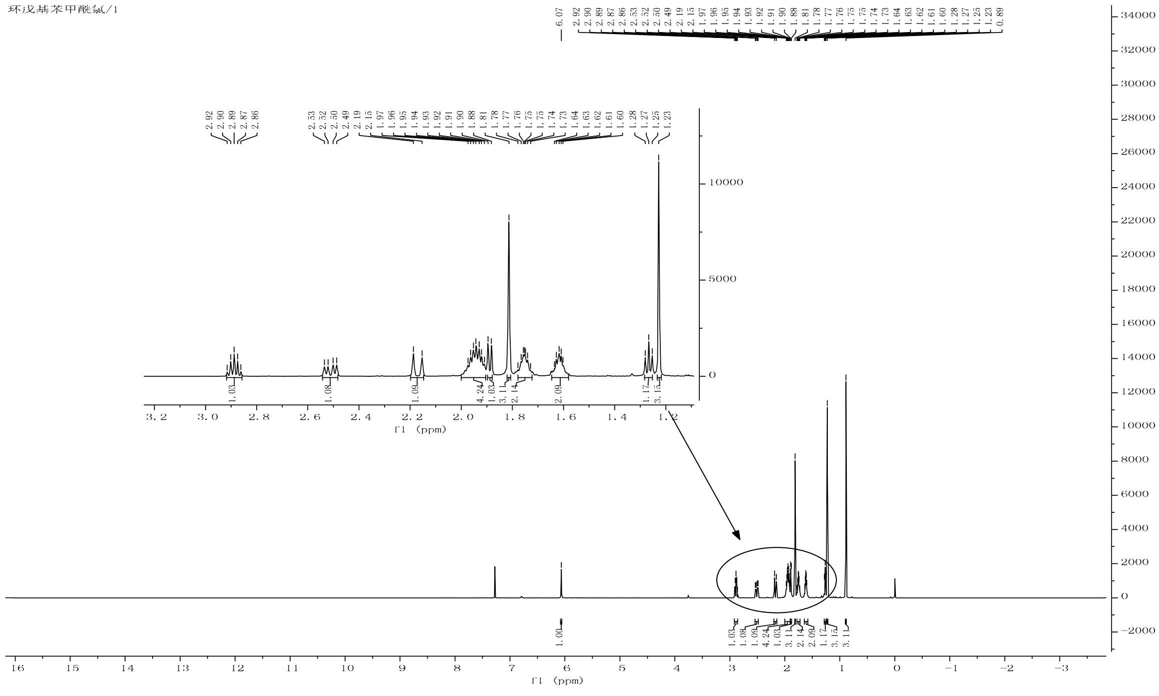

Figure S85. ^{1}H-NMR spectrum of compound (*Z*)-**4n** in $CDCl_3$.

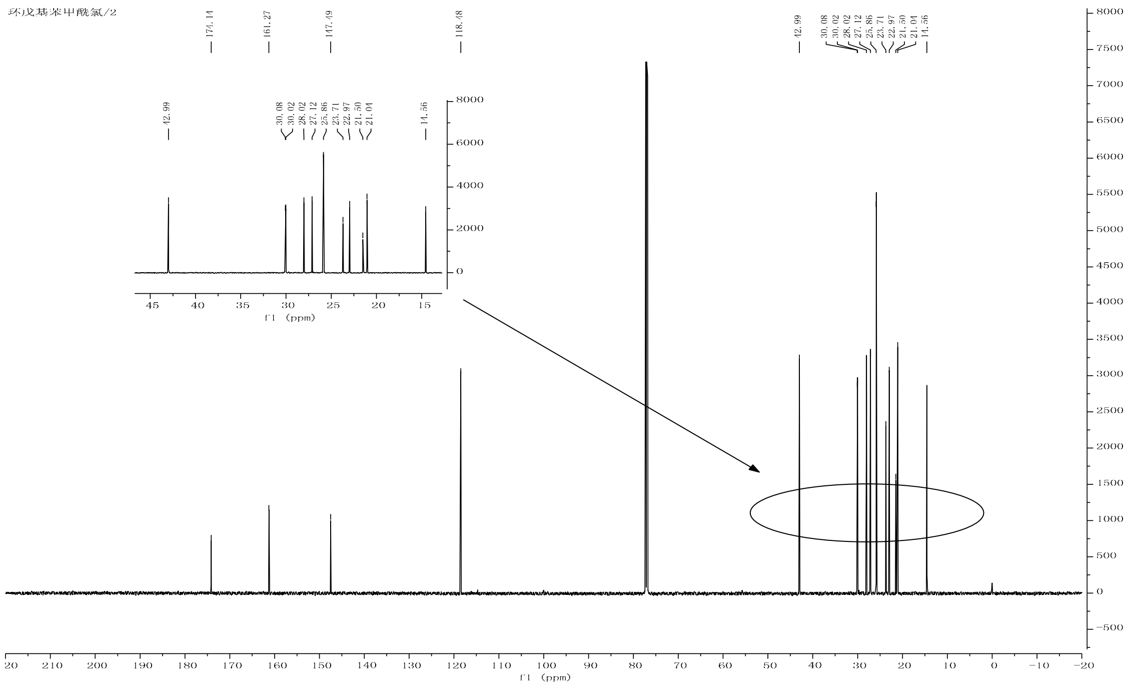

Figure S86. ^{13}C-NMR spectrum of compound (*Z*)-**4n** in $CDCl_3$.

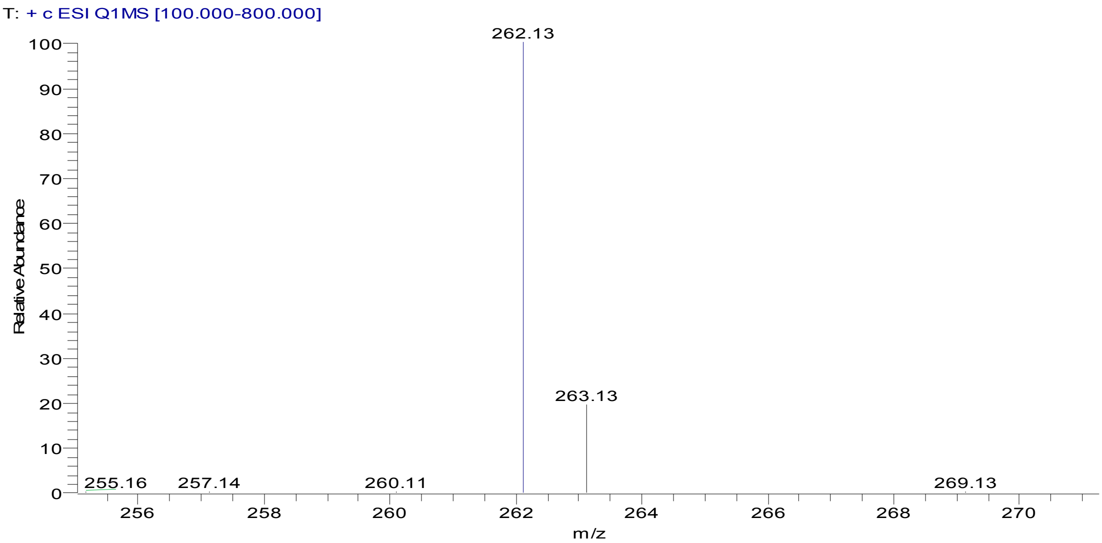

Figure S87. ESI-MS spectrum of compound (*Z*)-**4n.**

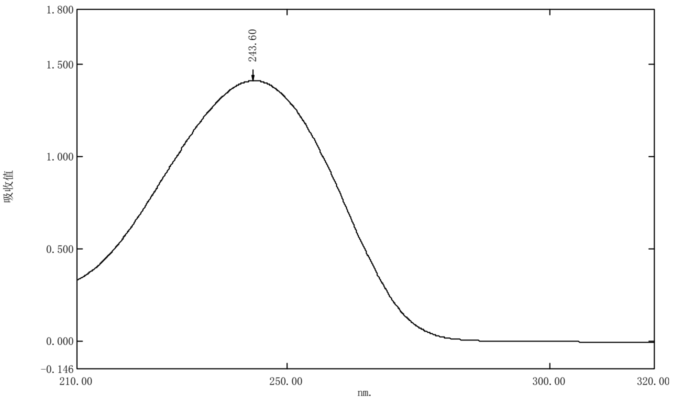

Figure S88. UV-vis spectrum of compound (*Z*)-**4o** in EtOH.

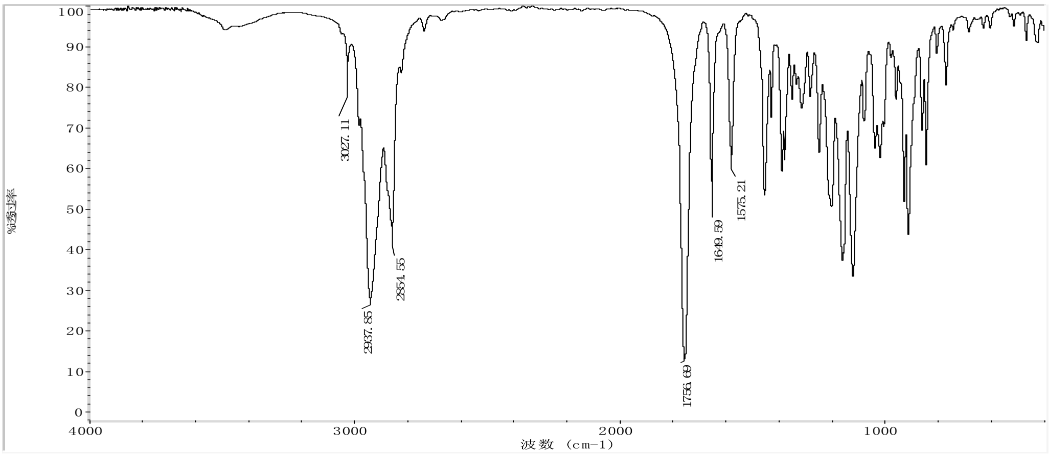

Figure S89. FTIR spectrum of compound (*Z*)-**4o**.

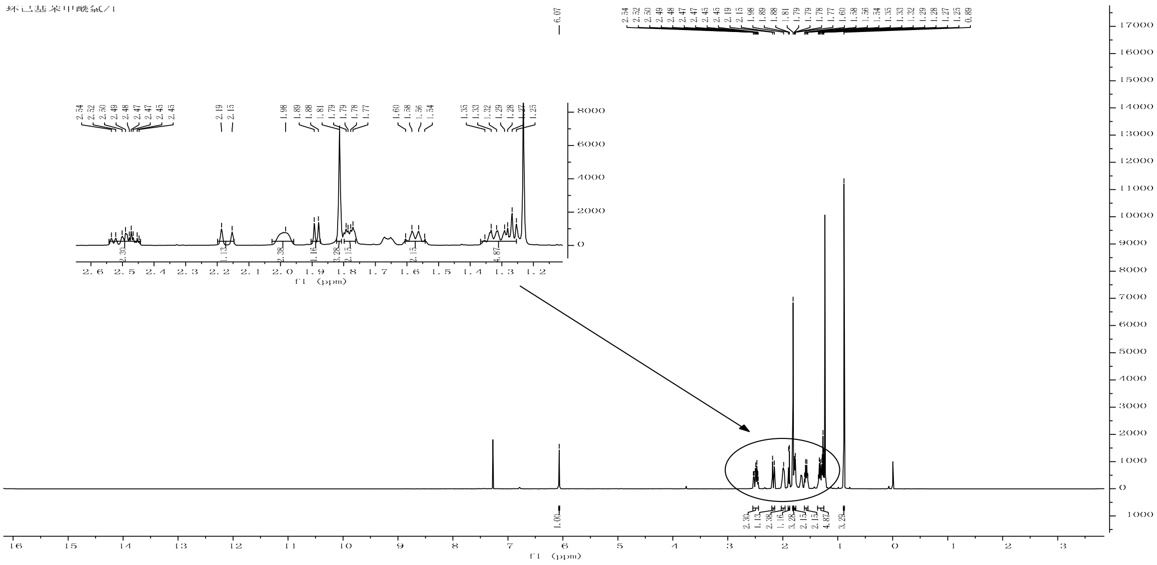

Figure S90. ^{1}H-NMR spectrum of compound (*Z*)-**4o** in $CDCl_3$.

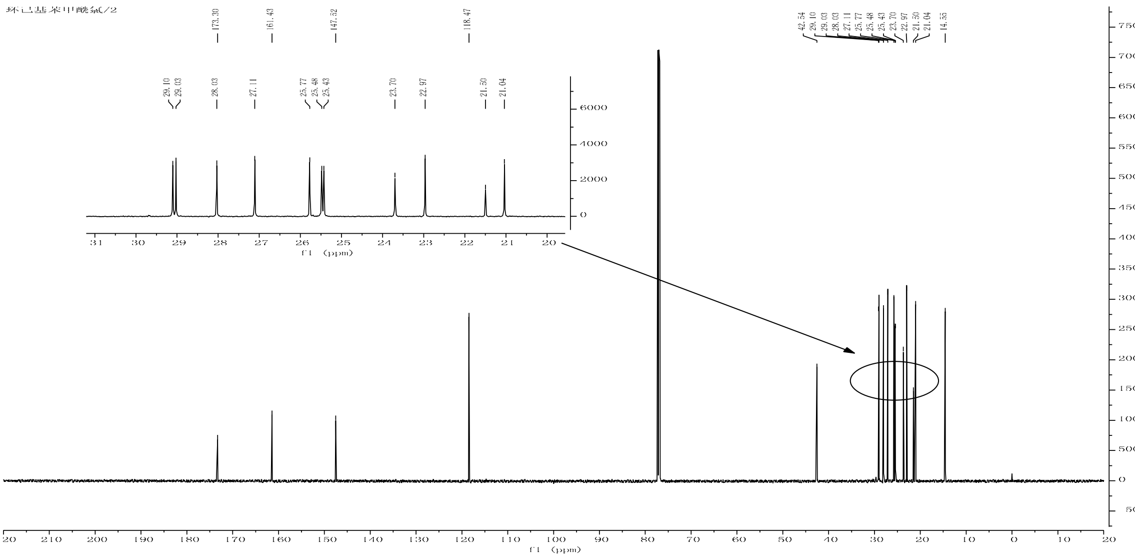

Figure S91. ^{13}C-NMR spectrum of compound (*Z*)-**4o** in $CDCl_3$.

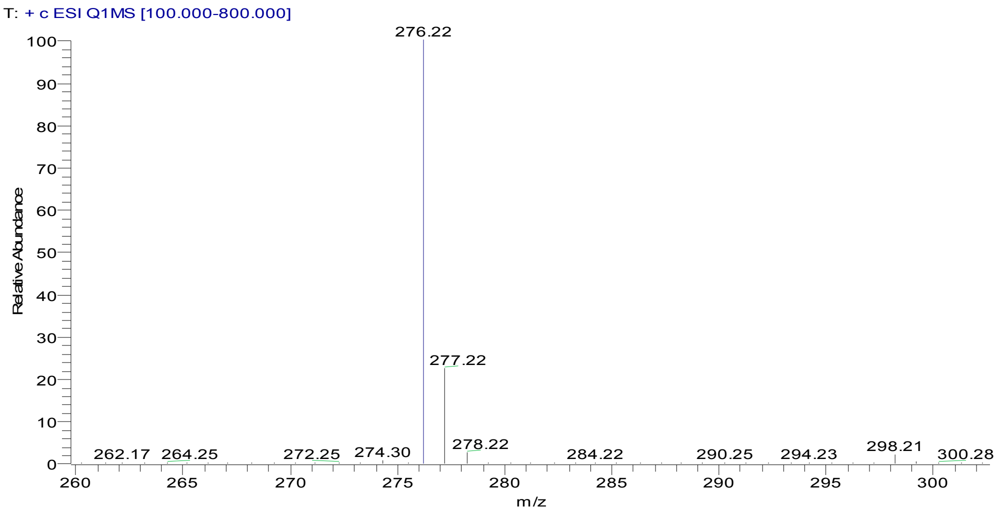

Figure S92. ESI-MS spectrum of compound (*Z*)-**4o.**

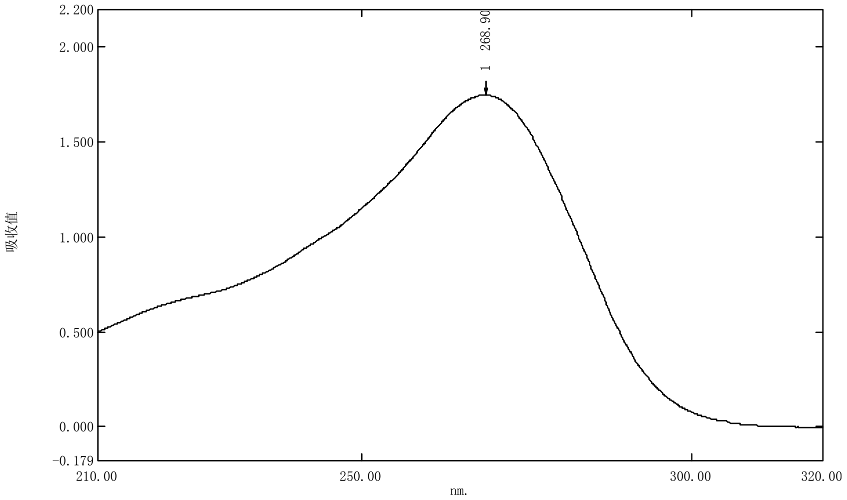

Figure S93. UV-vis spectrum of compound (*Z*)-**4p** in EtOH.

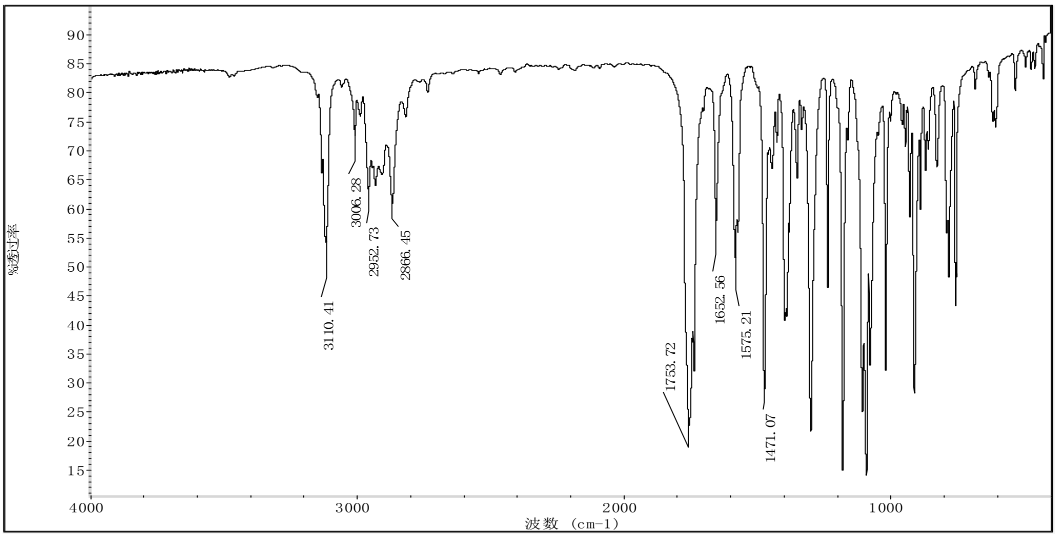

Figure S94. FTIR spectrum of compound (*Z*)-**4p**.

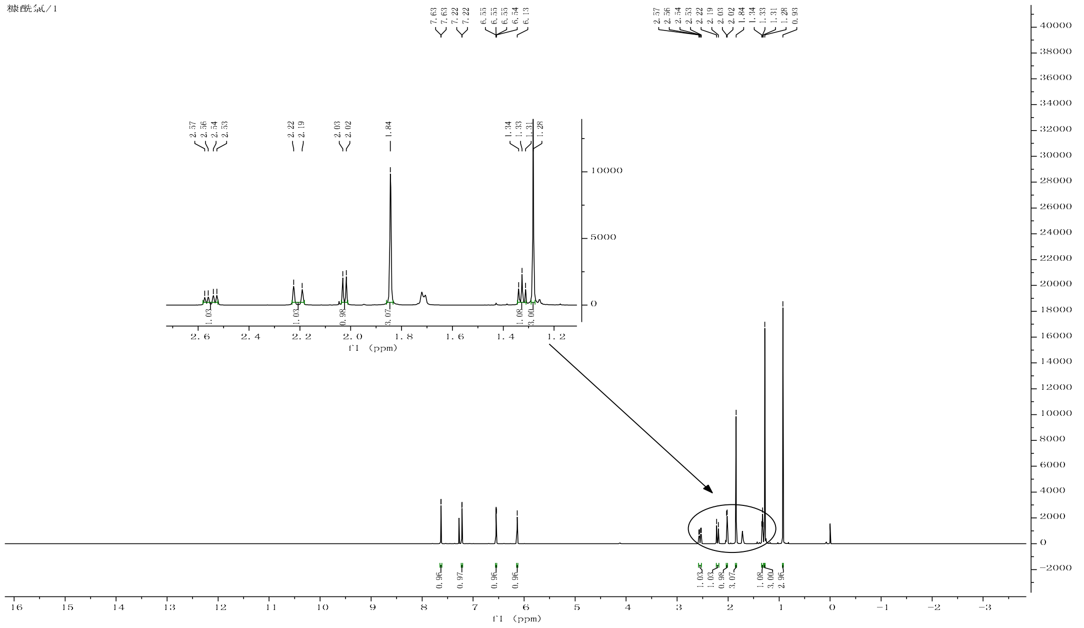

Figure S95. ^{1}H-NMR spectrum of compound (*Z*)-**4p** in $CDCl_3$.

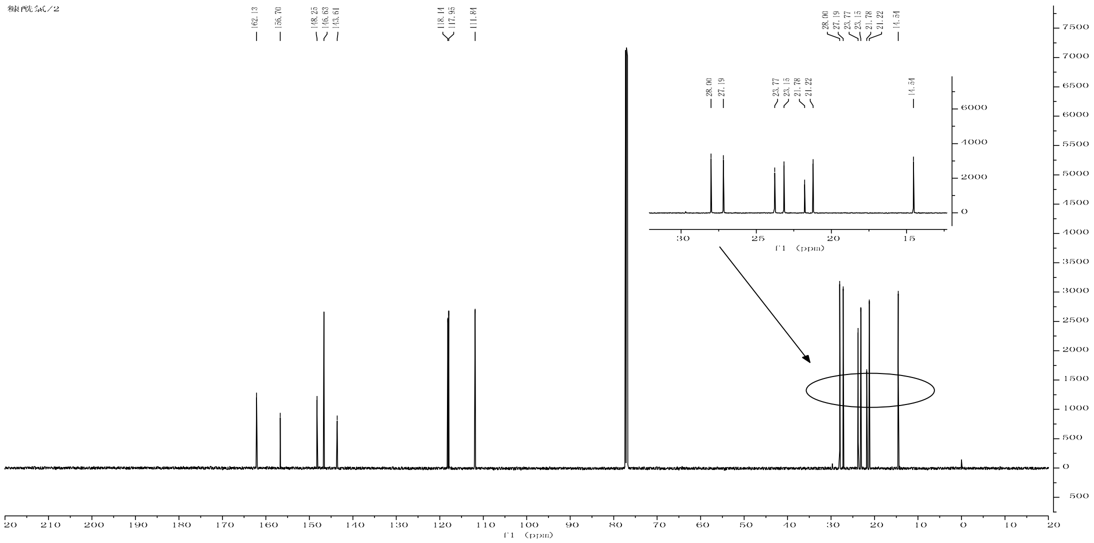

Figure S96. ^{13}C-NMR spectrum of compound (*Z*)-**4p** in $CDCl_3$.

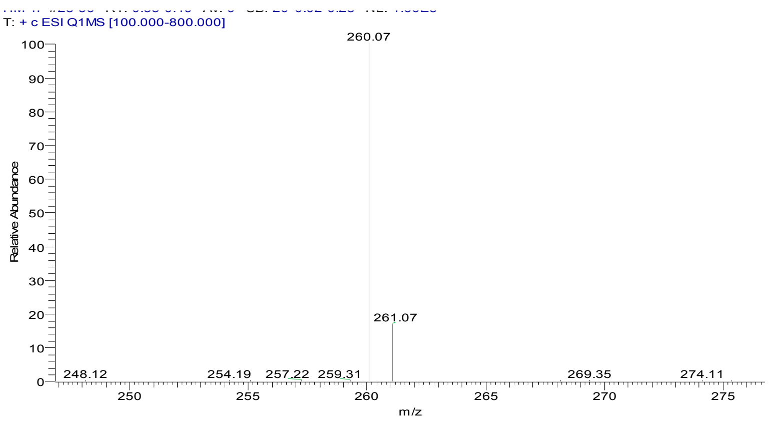

Figure S97. ESI-MS spectrum of compound (*Z*)-**4p.**

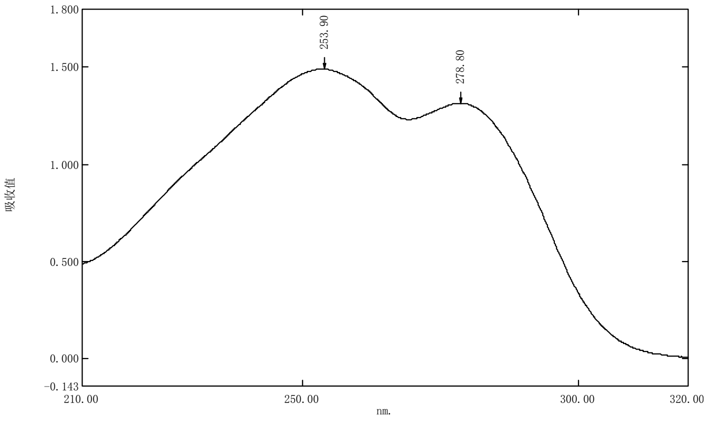

Figure S98. UV-vis spectrum of compound (*Z*)-**4q** in EtOH.

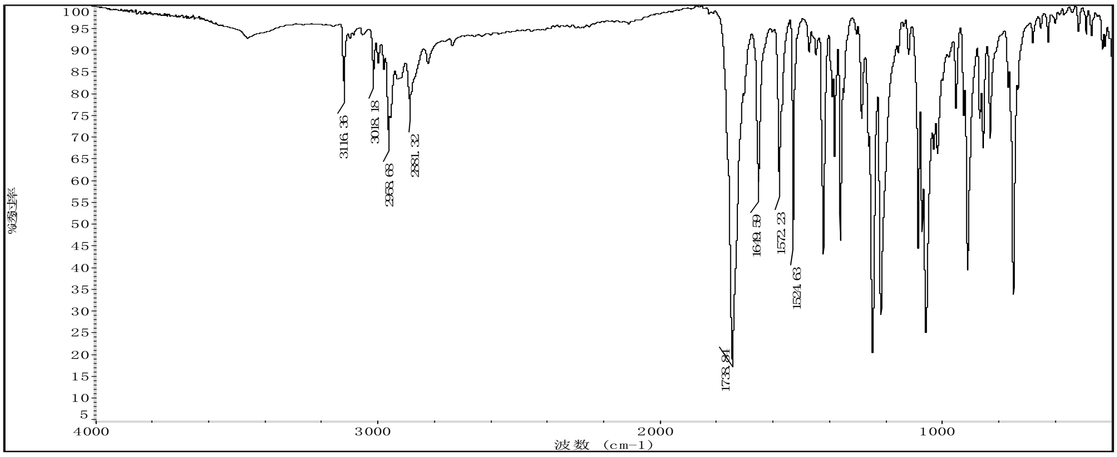

Figure S99. FTIR spectrum of compound (*Z*)-**4q**.

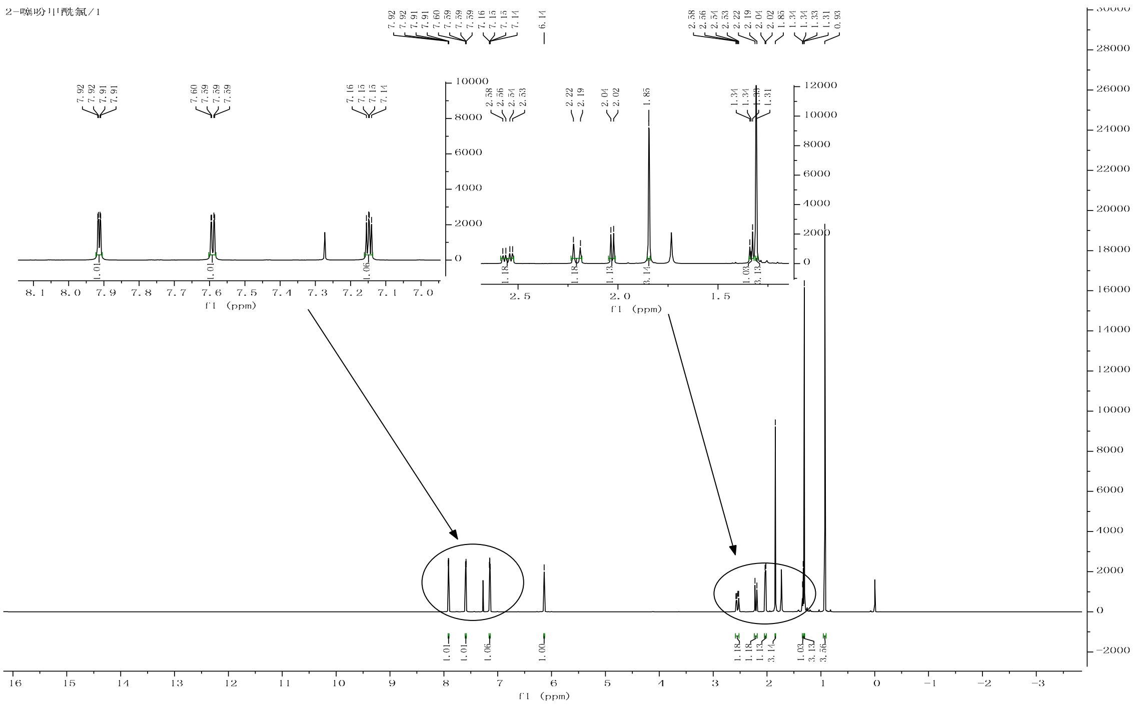

Figure S100. ^{1}H-NMR spectrum of compound (*Z*)-**4q** in $CDCl_3$.

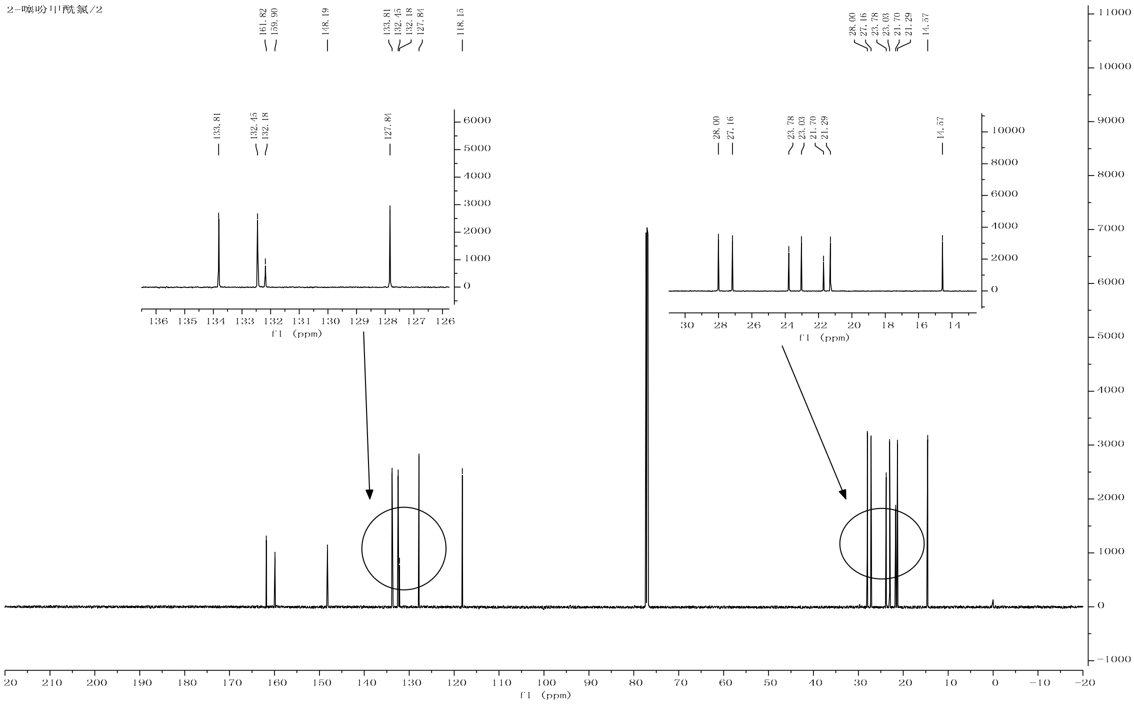

Figure S101. ^{13}C-NMR spectrum of compound (*Z*)-**4q** in $CDCl_3$.

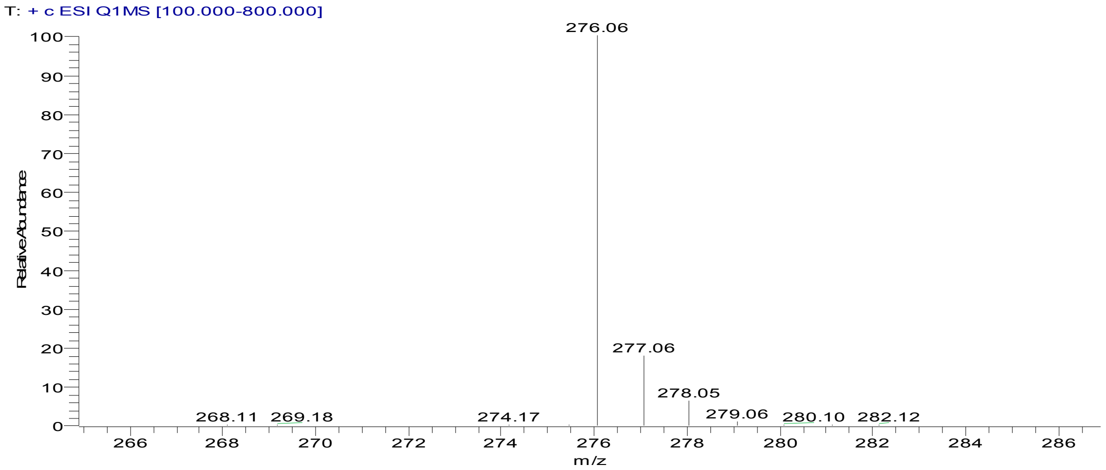

Figure S102. ESI-MS spectrum of compound (*Z*)-**4q.**

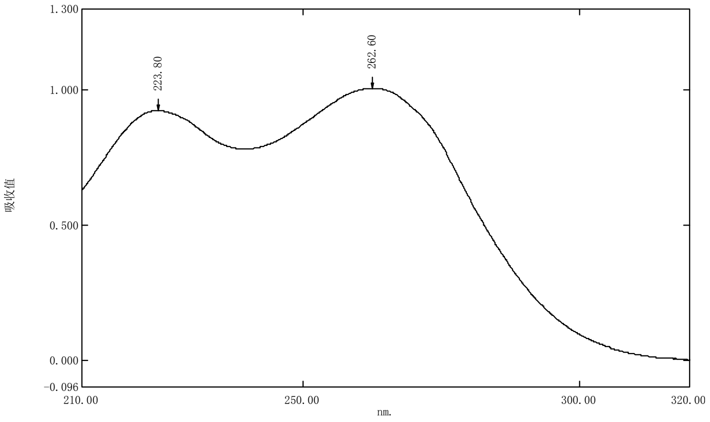

Figure S103. UV-vis spectrum of compound (*Z*)-**4r** in EtOH.

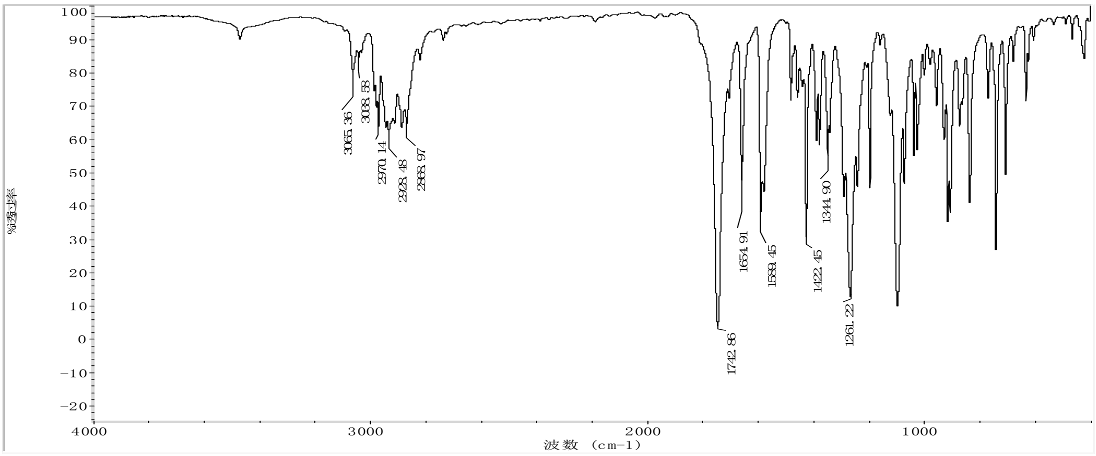

Figure S104. FTIR spectrum of compound (*Z*)-**4r**.

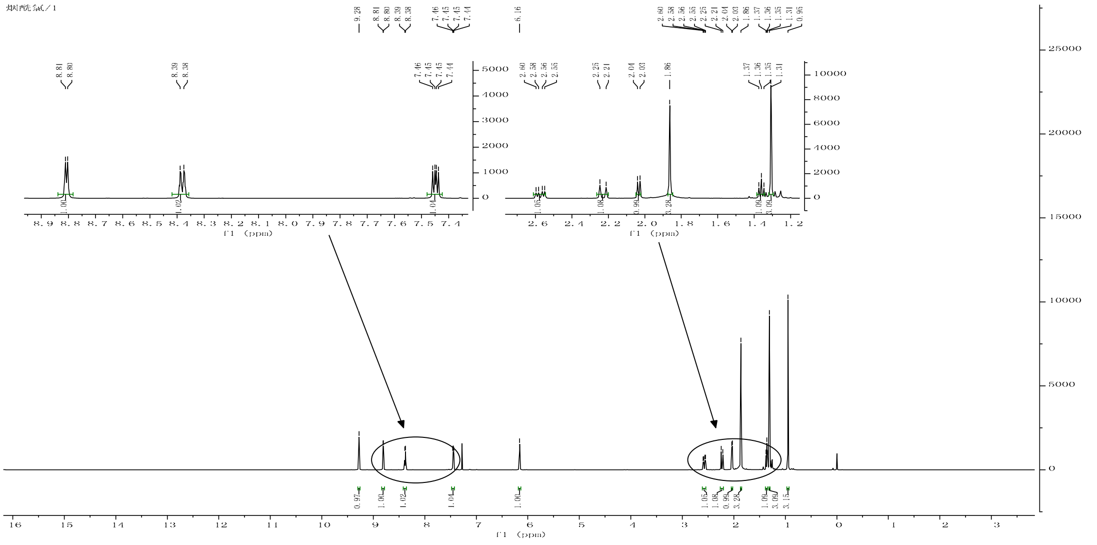

Figure S105. ^{1}H-NMR spectrum of compound (*Z*)-**4r** in $CDCl_3$.

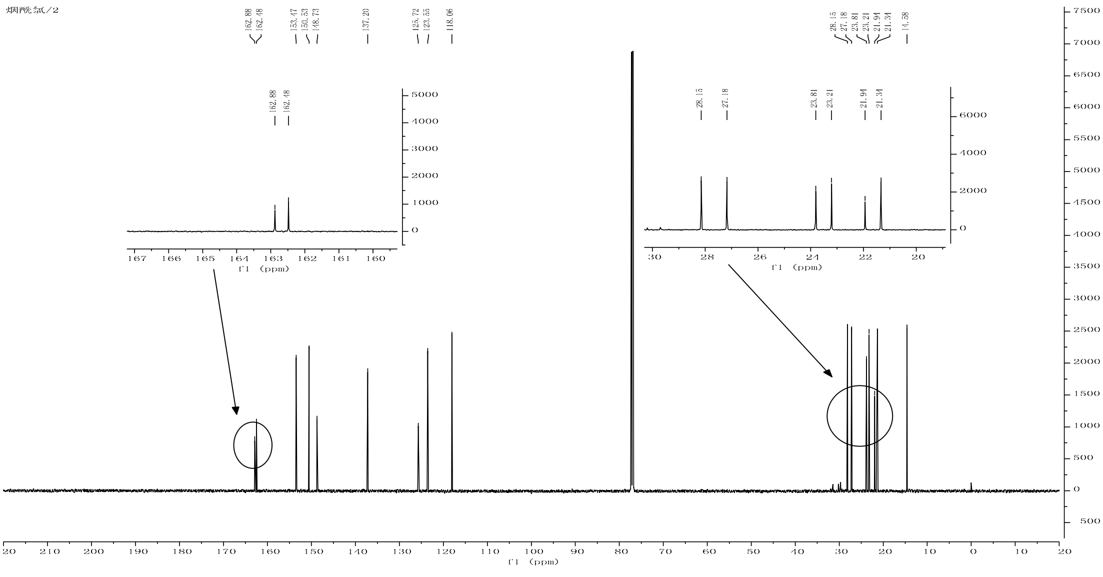

Figure S106. ^{13}C-NMR spectrum of compound (*Z*)-**4r** in $CDCl_3$.

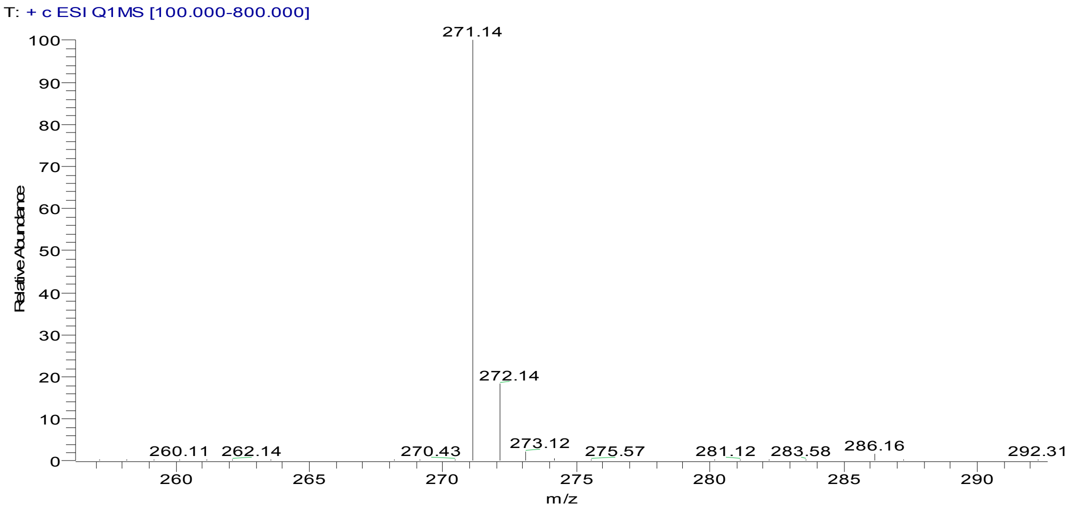

Figure S107. ESI-MS spectrum of compound (*Z*)-**4r.**

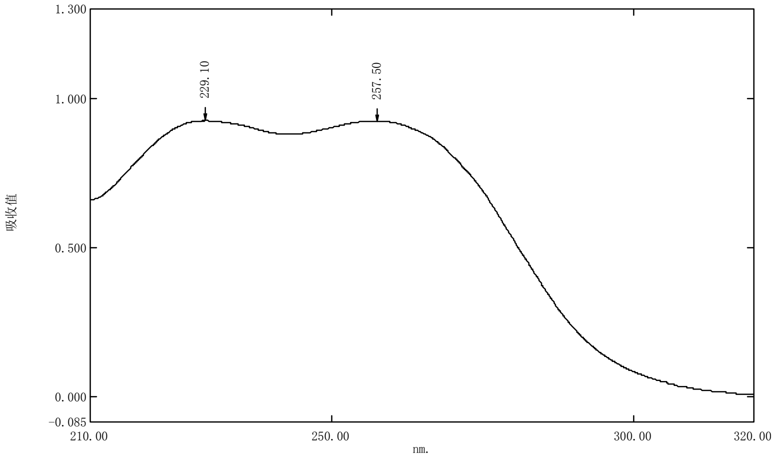

Figure S108. UV-vis spectrum of compound (*Z*)-**4s** in EtOH.

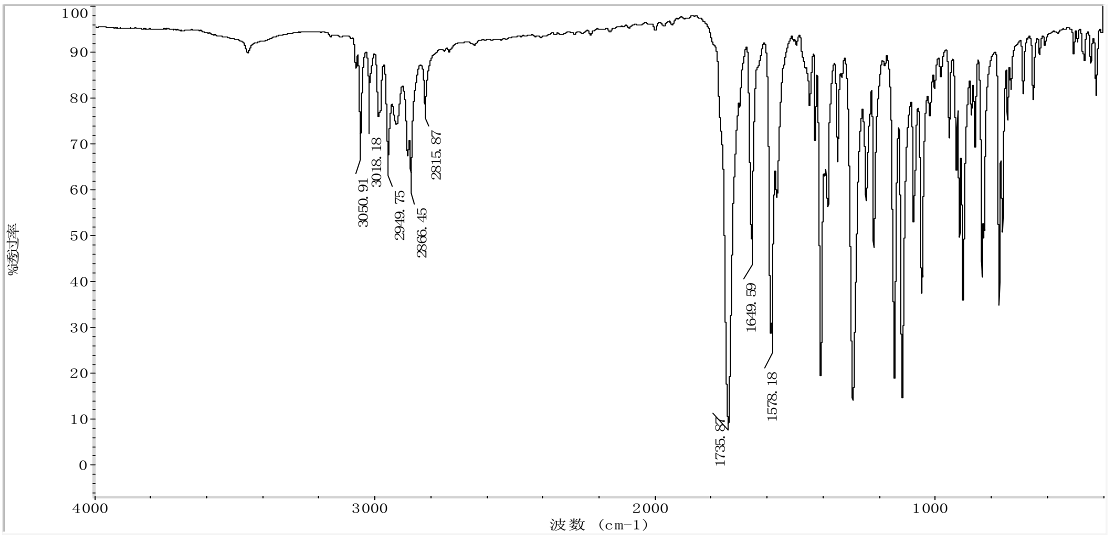

Figure S109. FTIR spectrum of compound (*Z*)-**4s**.

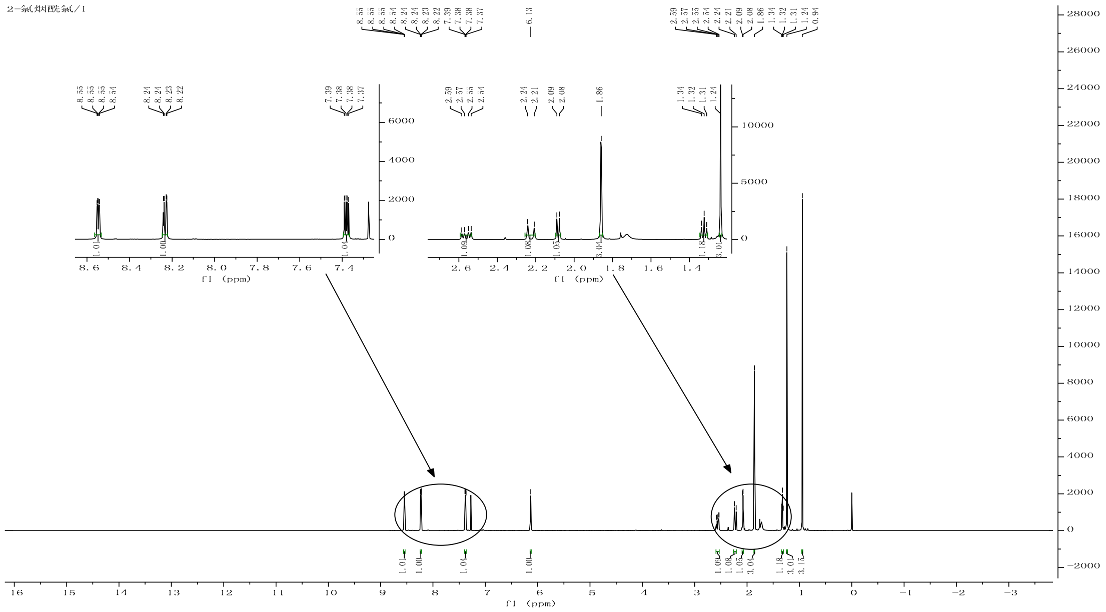

Figure S110. ^{1}H-NMR spectrum of compound **4s** in $CDCl_3$.

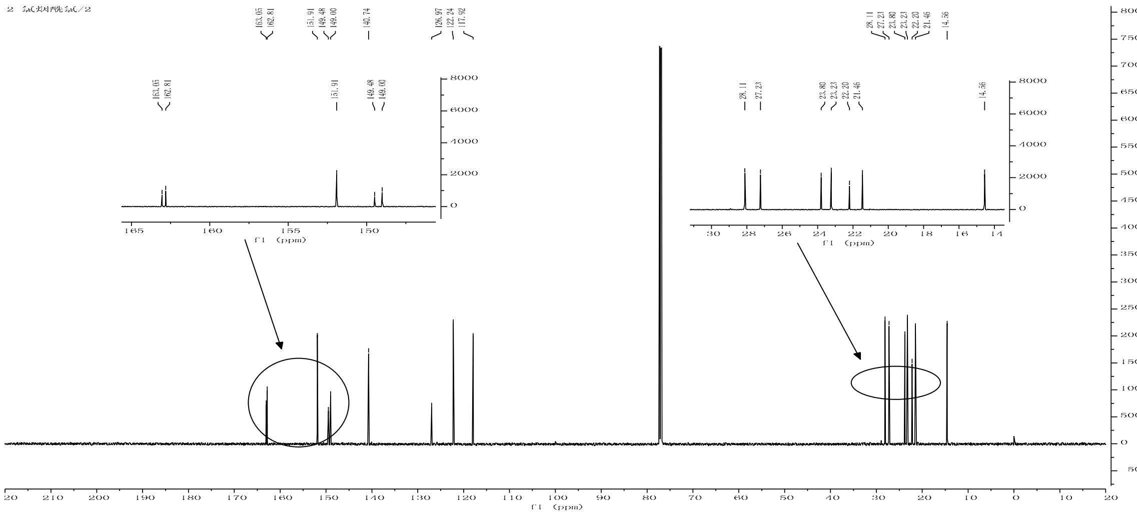

Figure S111. ^{13}C-NMR spectrum of compound (*Z*)-**4s** in $CDCl_3$.

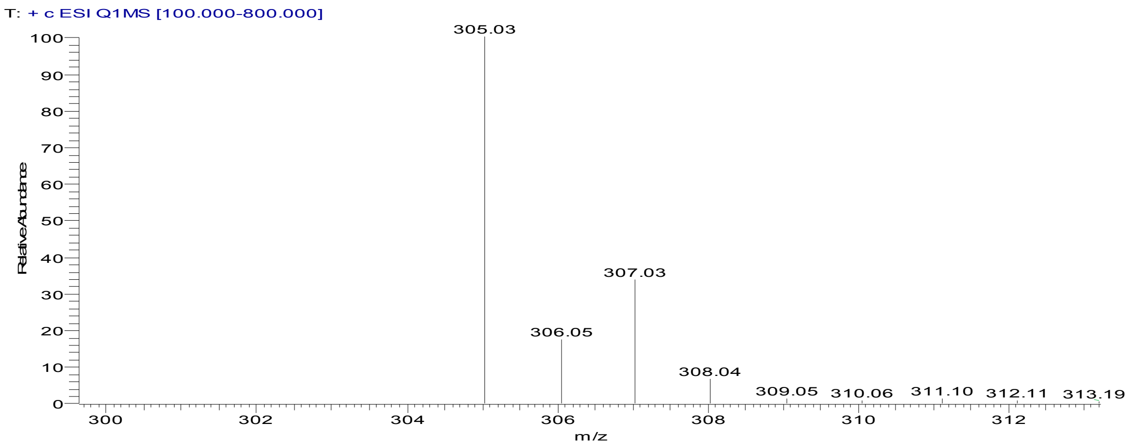

Figure S112. ESI-MS spectrum of compound (*Z*)-**4s.**

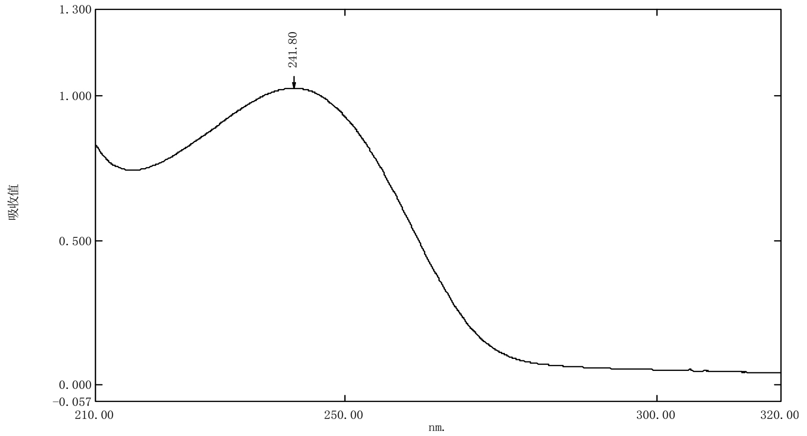

Figure S113. UV-vis spectrum of compound (*Z*)-**4t** in EtOH.

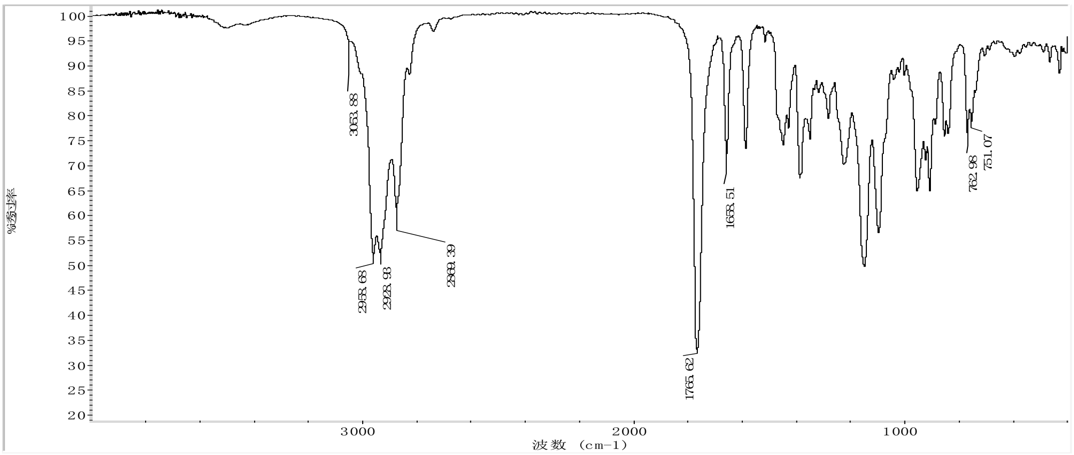

Figure S114. FTIR spectrum of compound (*Z*)-**4t**.

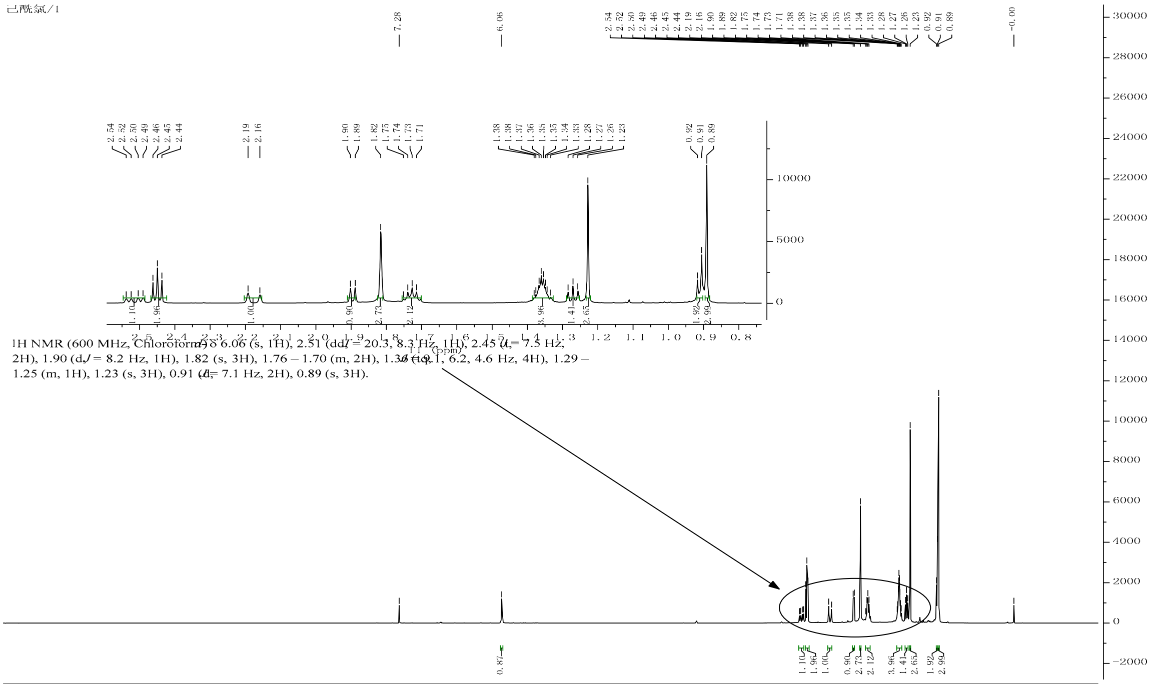

Figure S115. ^{1}H-NMR spectrum of compound (*Z*)-**4**) in $CDCl_3$.

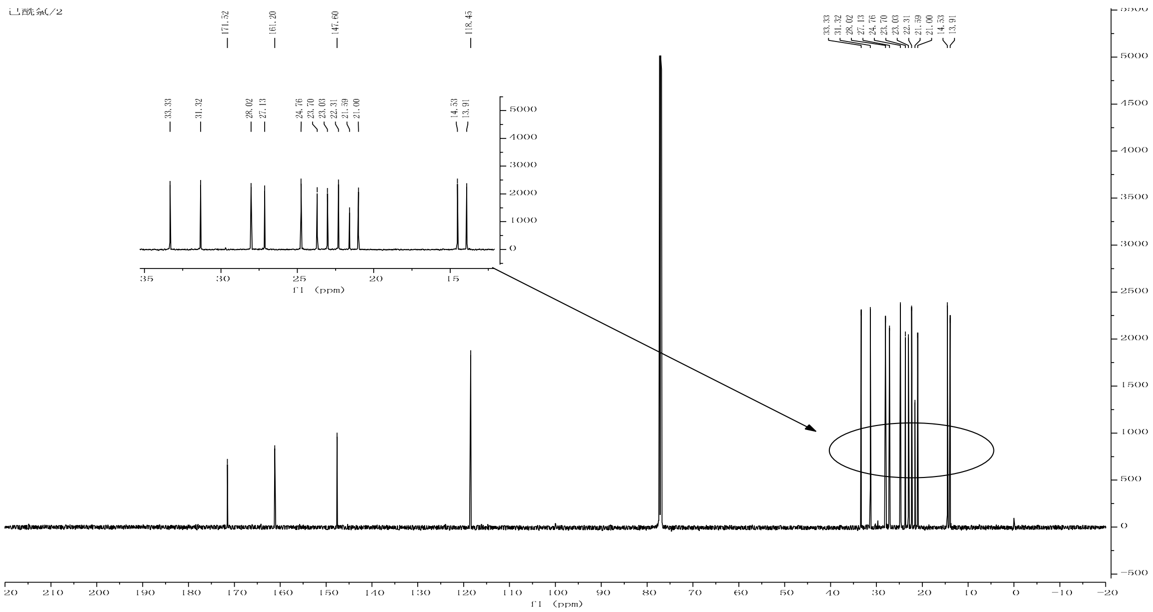

Figure S116. ^{13}C-NMR spectrum of compound (*Z*)-**4t** in $CDCl_3$.

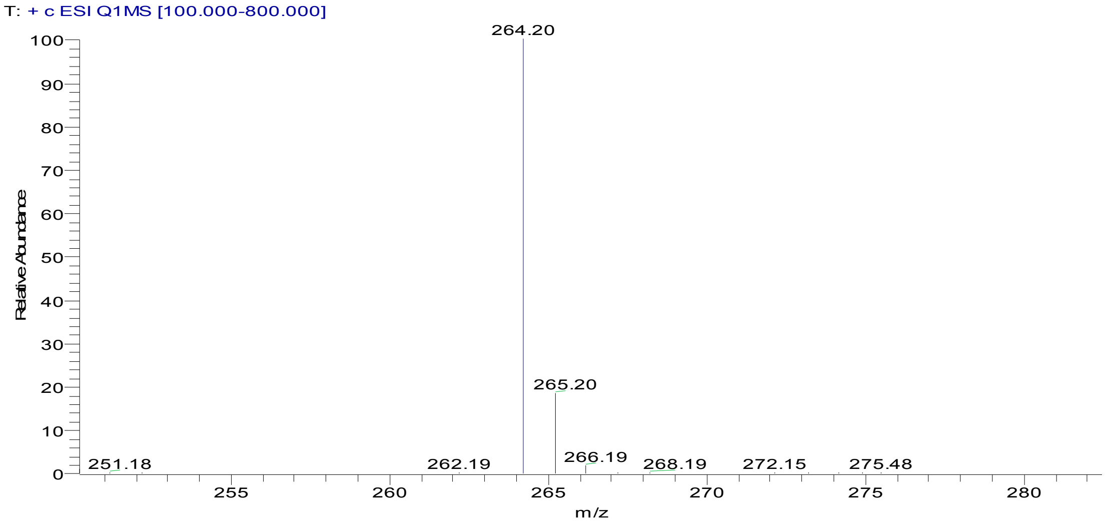

Figure S117. ESI-MS spectrum of compound (*Z*)-**4t.**

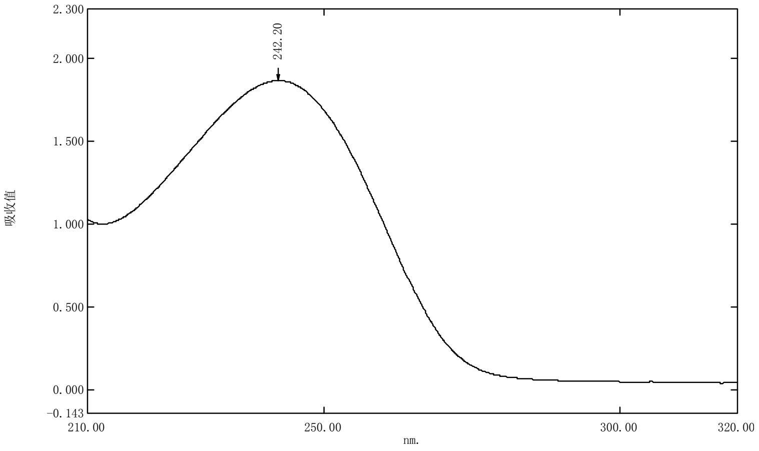

Figure S118. UV-vis spectrum of compound (*Z*)-**4u** in EtOH.

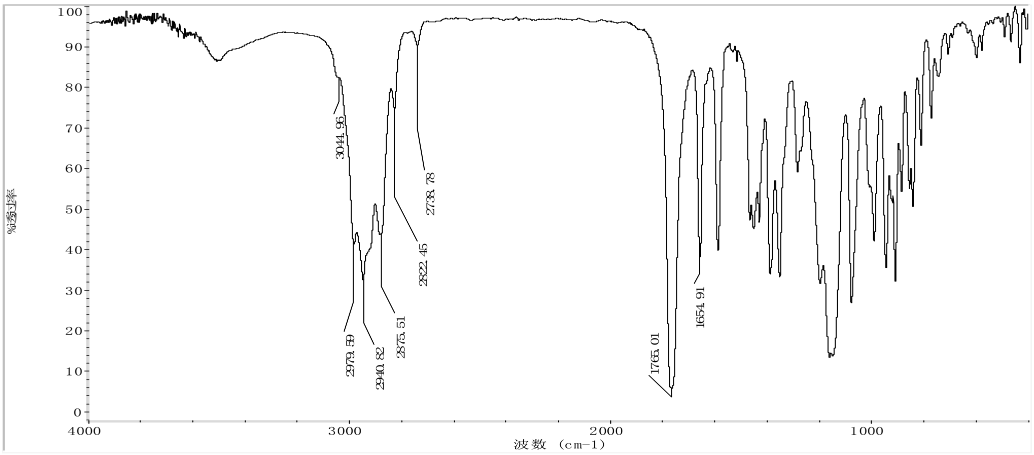

Figure S119. FTIR spectrum of compound (*Z*)-**4u**.

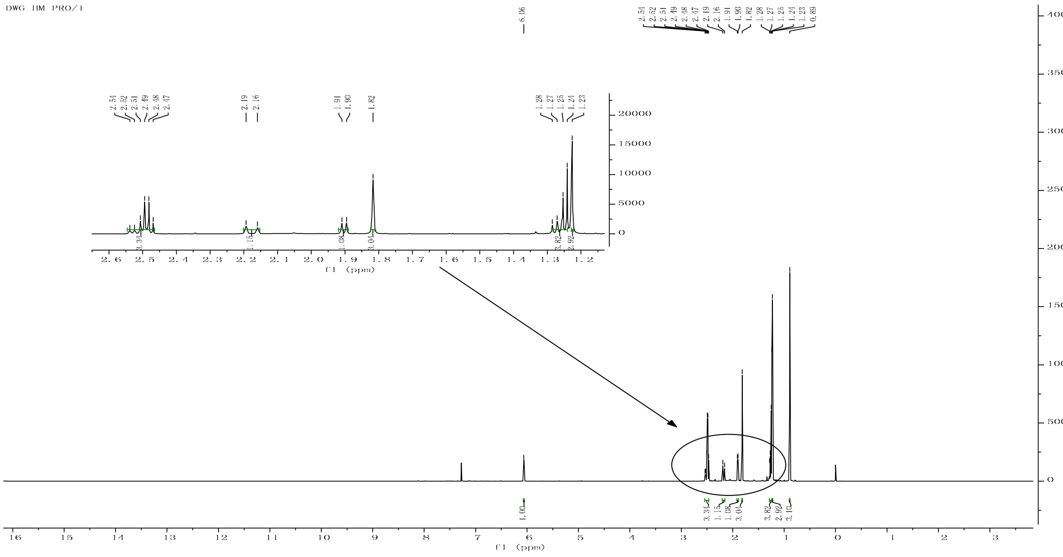

Figure S120. ^{1}H-NMR spectrum of compound (*Z*)-**4u** in $CDCl_3$.

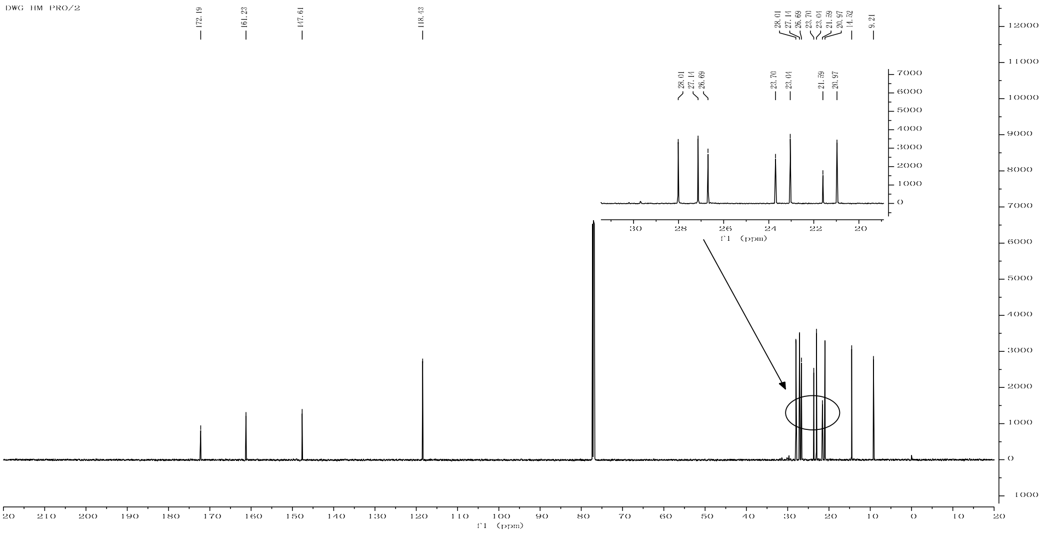

Figure S121. ^{13}C-NMR spectrum of compound (*Z*)-**4u** in $CDCl_3$.

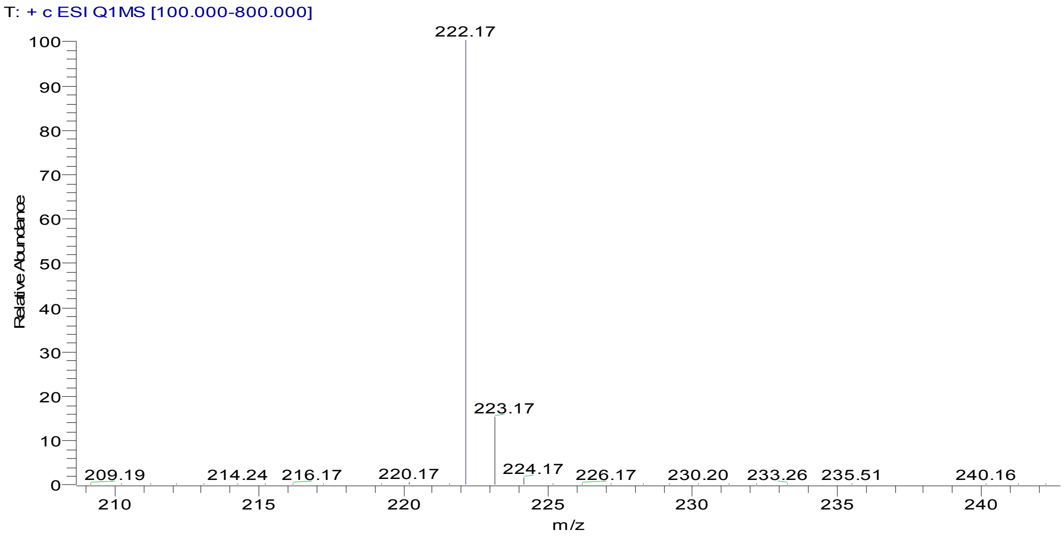

Figure S122. ESI-MS spectrum of compound (*Z*)-**4u.**

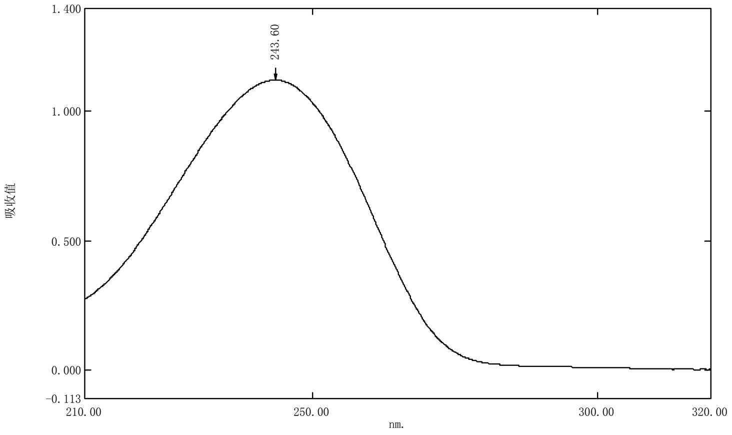

Figure S123. UV-vis spectrum of compound (*Z*)-**4v** in EtOH.

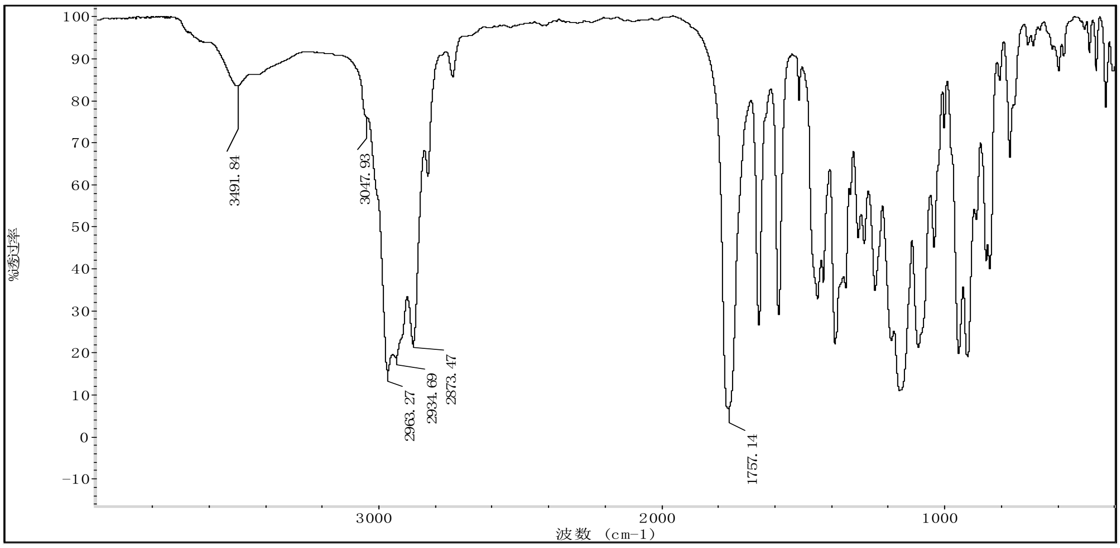

Figure S124. FTIR spectrum of compound (*Z*)-**4v**.

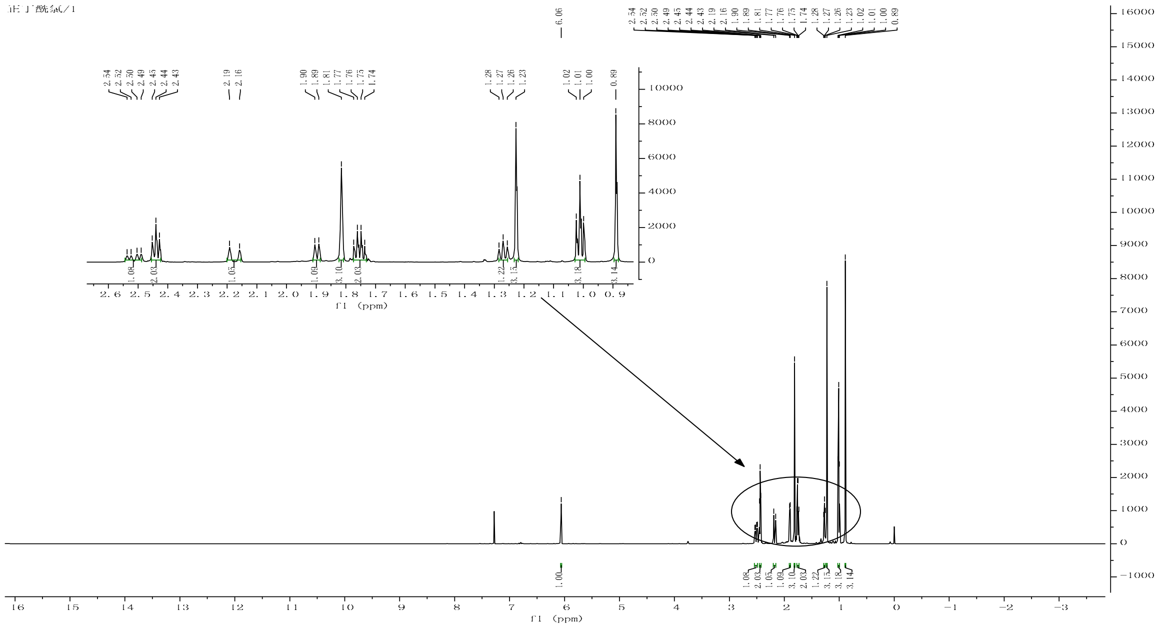

Figure S125. ^{1}H-NMR spectrum of compound (*Z*)-**4v** in $CDCl_3$.

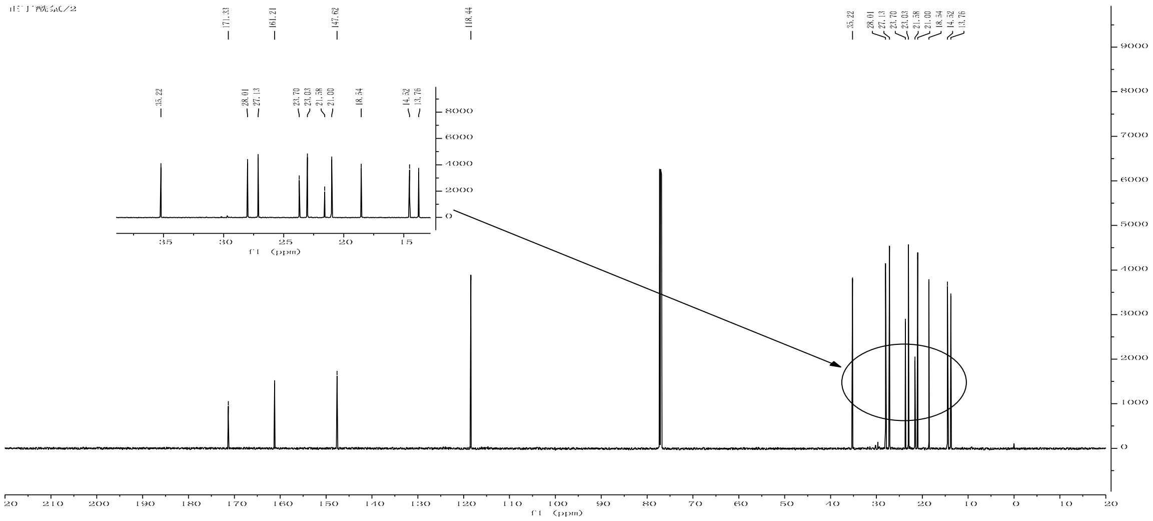

Figure S126 ^{13}C-NMR spectrum of compound (*Z*)-**4v** in $CDCl_3$.

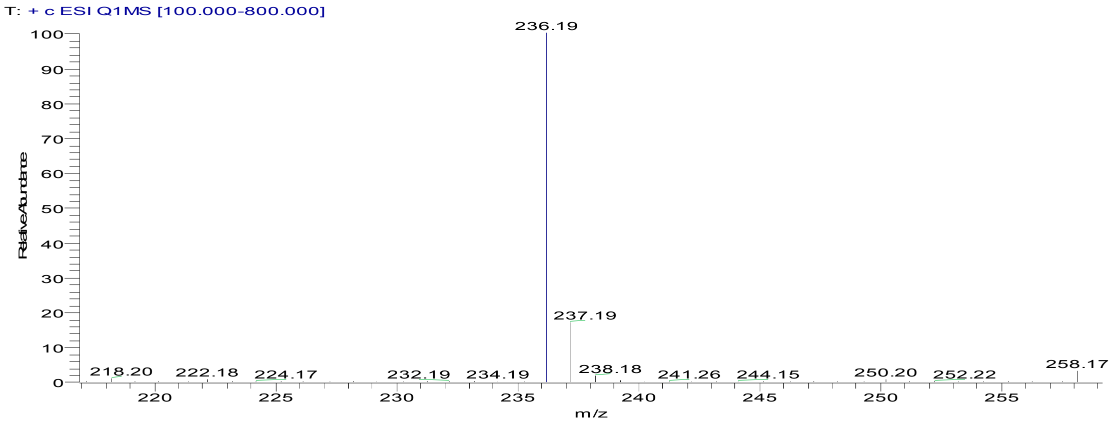

Figure S127. ESI-MS spectrum of compound (*Z*)-**4v.**

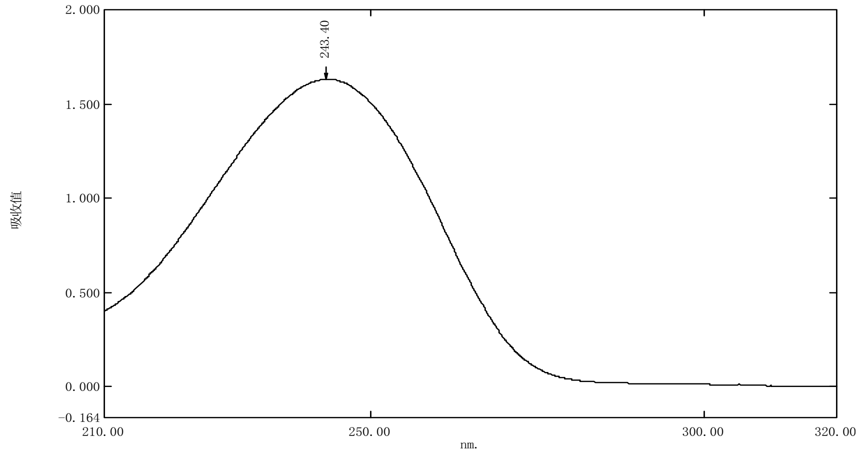

Figure S128. UV-vis spectrum of compound (*Z*)-**4w** in EtOH.

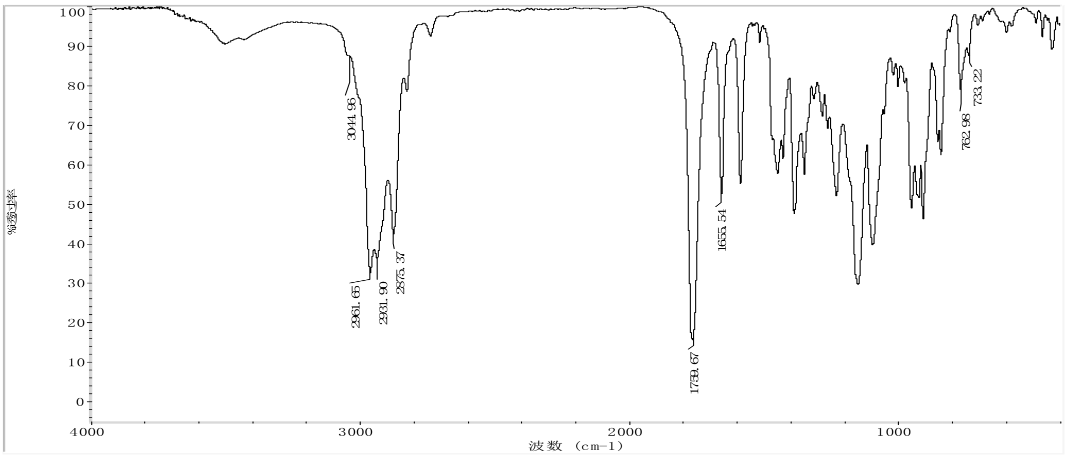

Figure S129. FTIR spectrum of compound (*Z*)-**4w**.

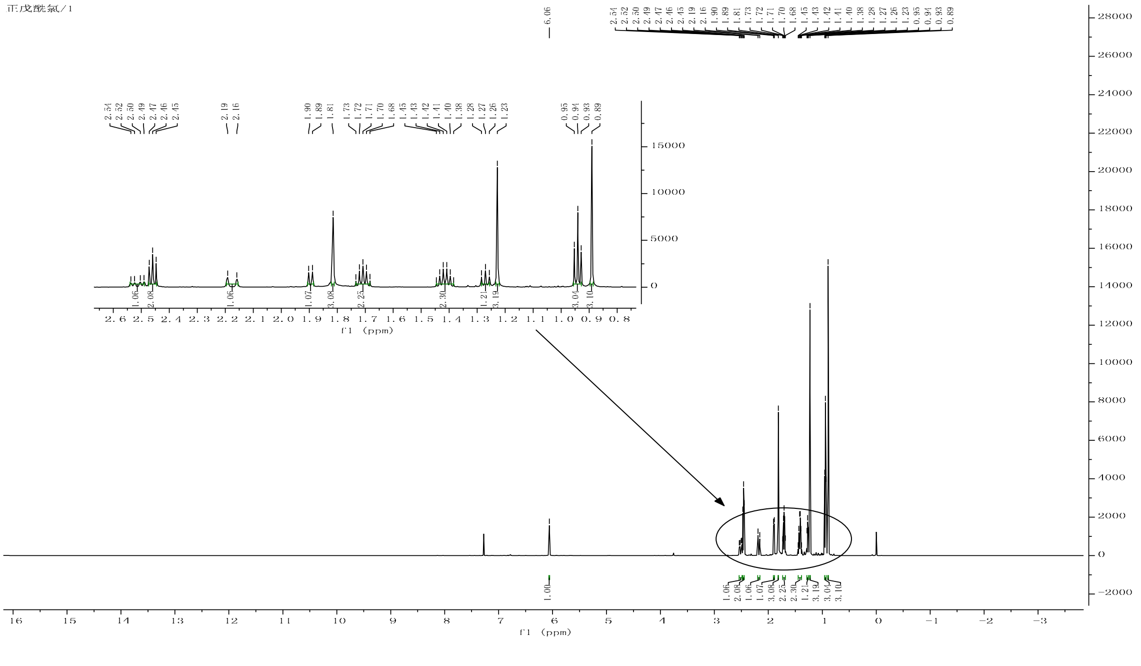

Figure S130. ^{1}H-NMR spectrum of compound (*Z*)-**4w** in $CDCl_3$.

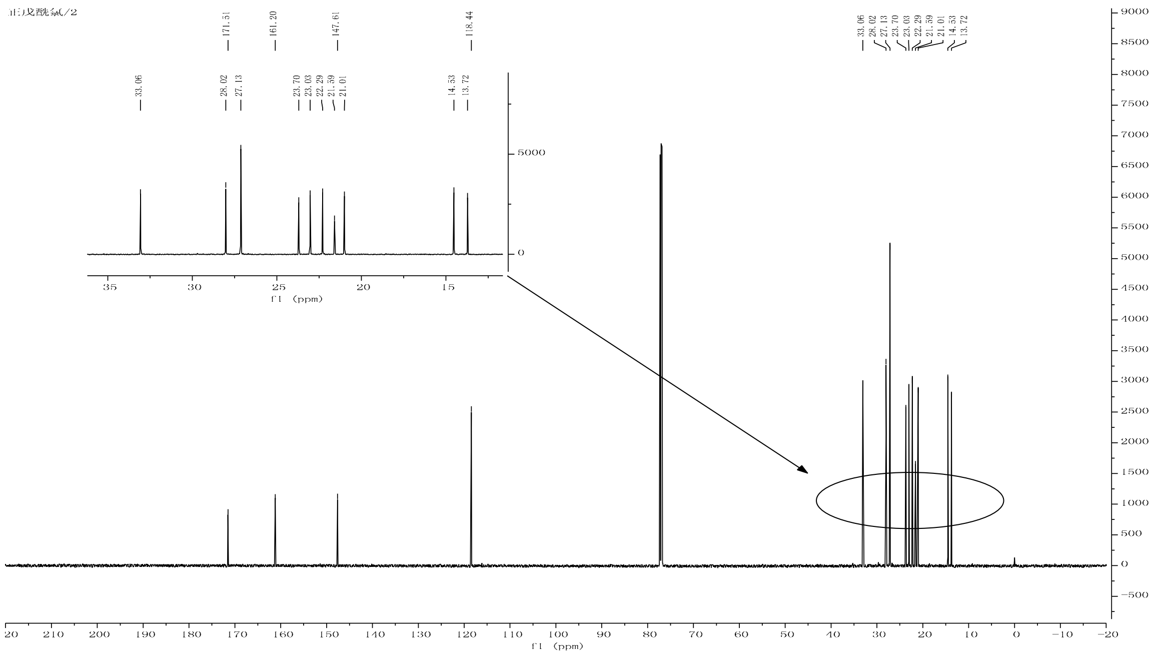

Figure S131. ^{13}C-NMR spectrum of compound (*Z*)-**4w** in $CDCl_3$.

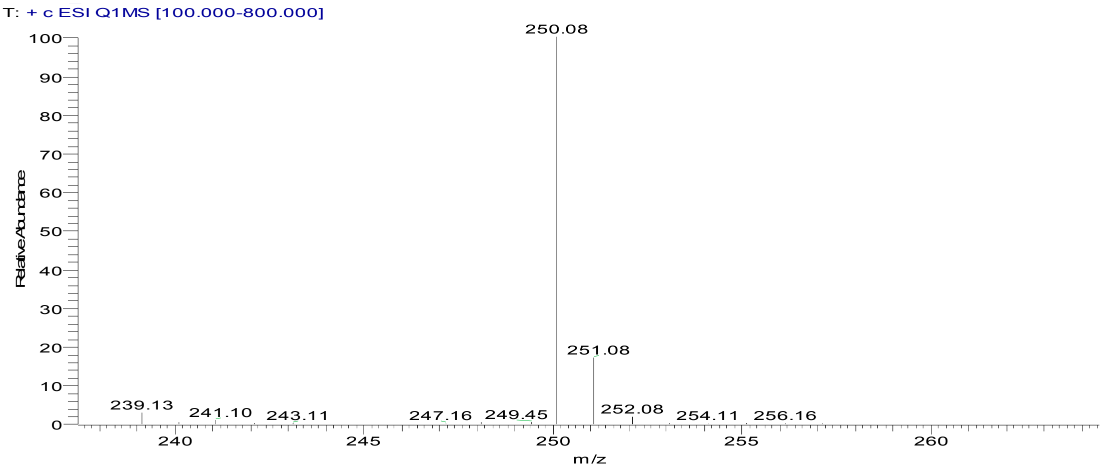

Figure S132. ESI-MS spectrum of compound (*Z*)-**4w.**

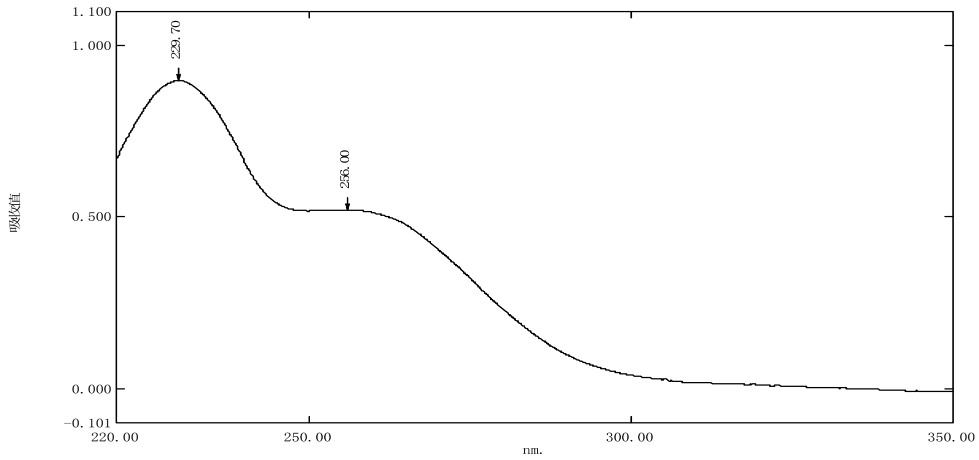

Figure S133. UV-vis spectrum of compound (*E*)-**4f′**.

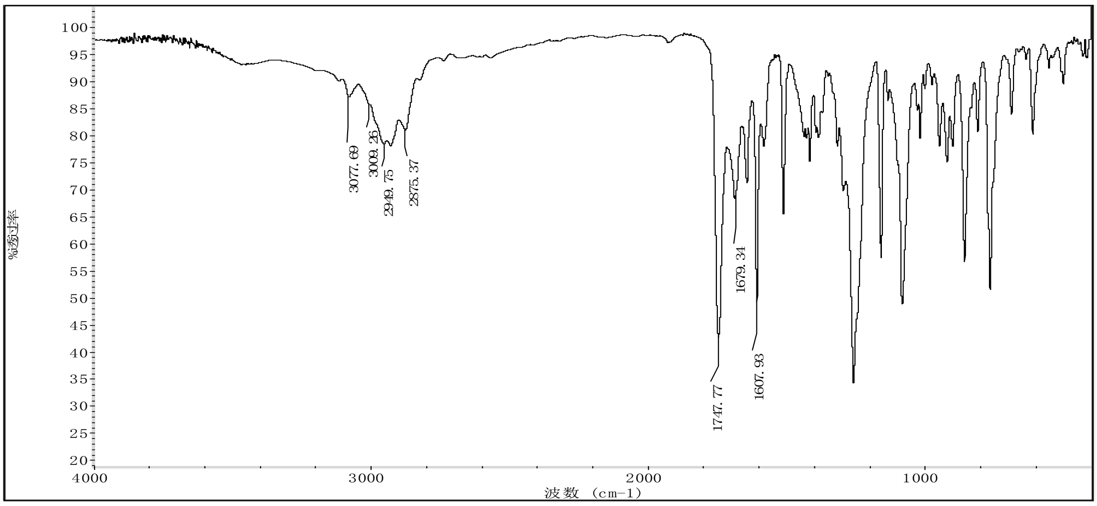

Figure S134. FTIR spectrum of compound (*E*)-**4f'**.

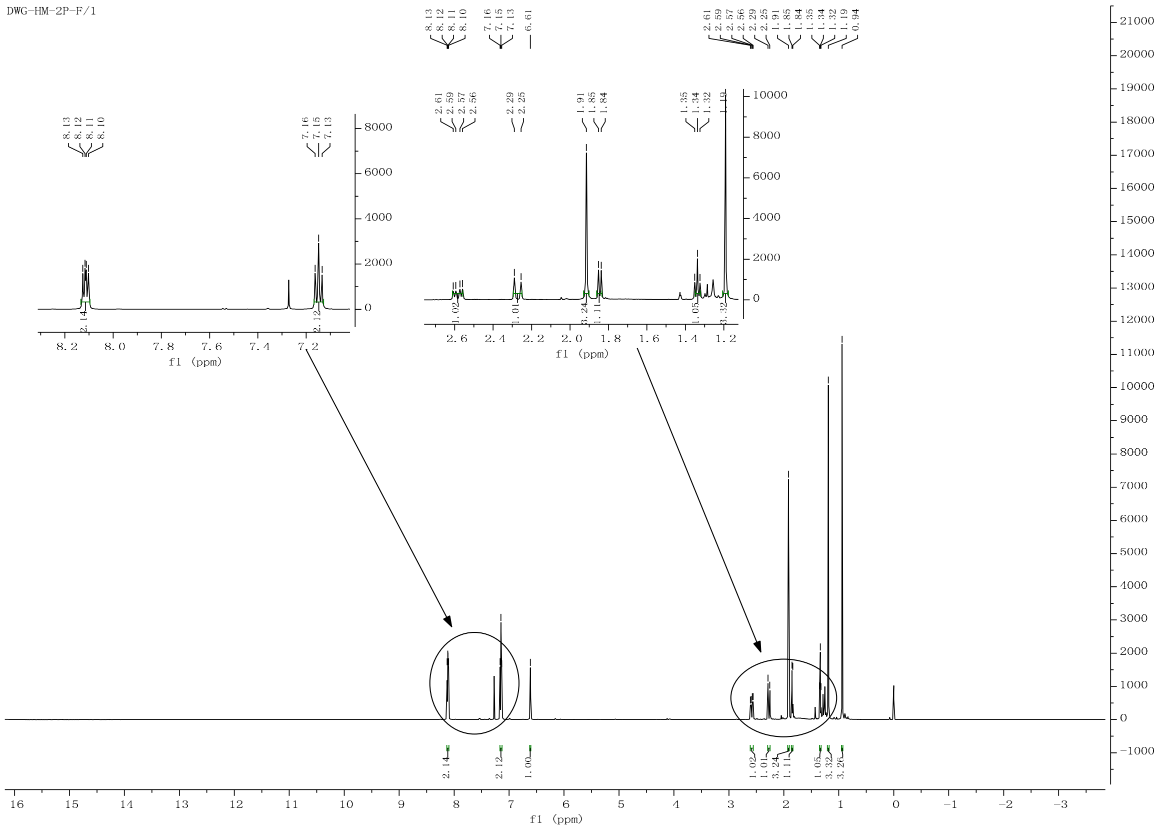

Figure S135. ^{1}H-NMR spectrum of compound (*E*)-**4f'** in $CDCl_3$.

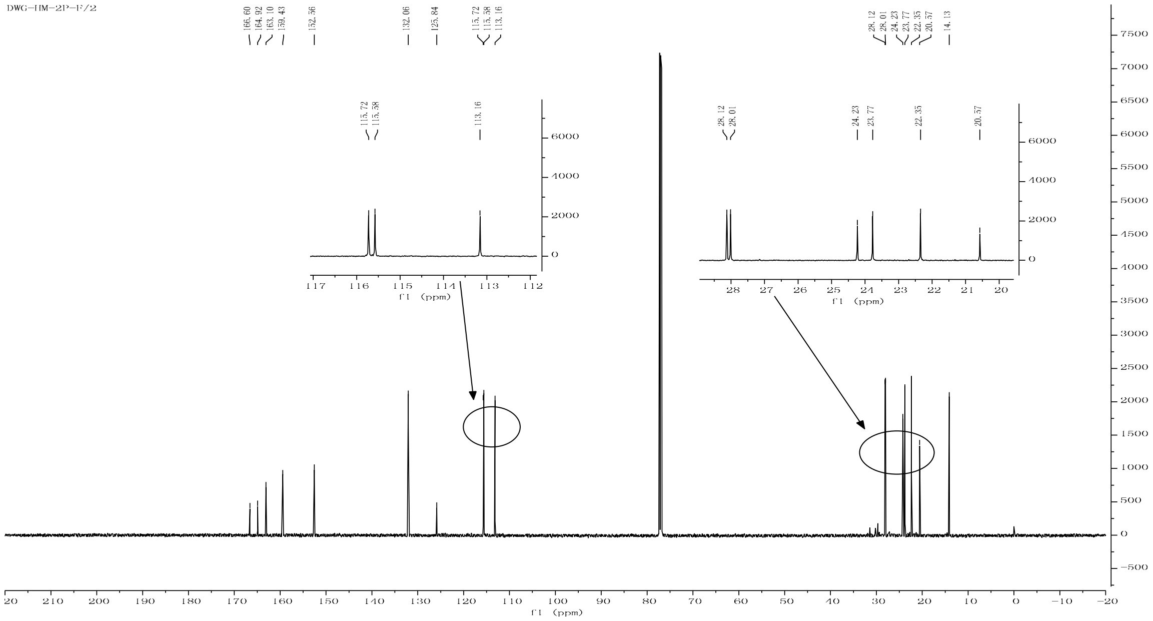

Figure S136. ^{13}C-NMR spectrum of compound (*E*)-**4f′** in $CDCl_3$.

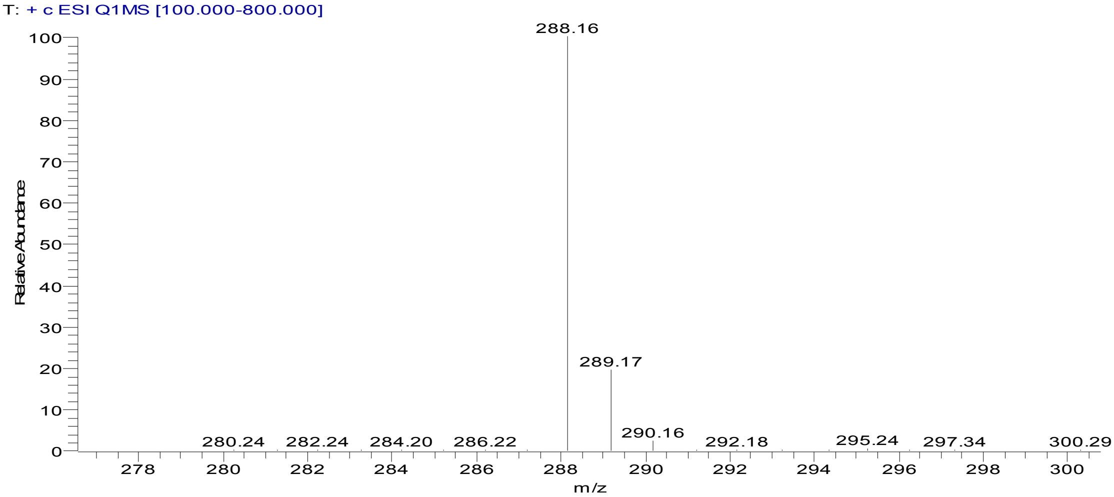

Figure S137. ESI-MS spectrum of compound (*E*)-**4f′**.

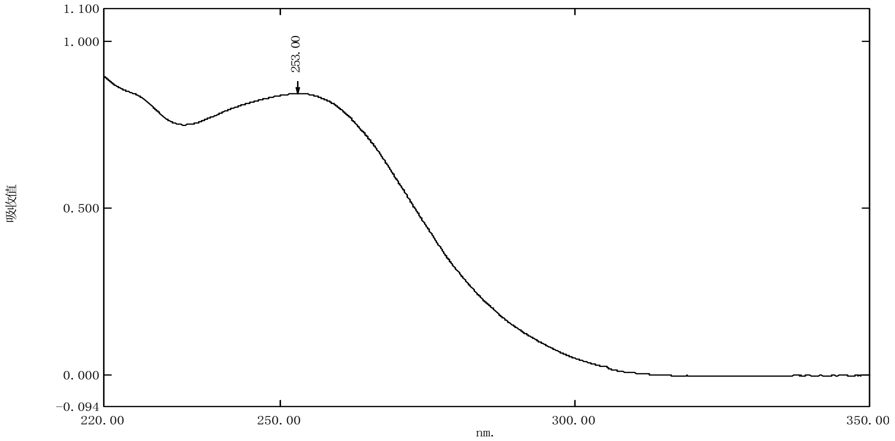

Figure S138. UV-vis spectrum of compound (*E*)-**4l′**.

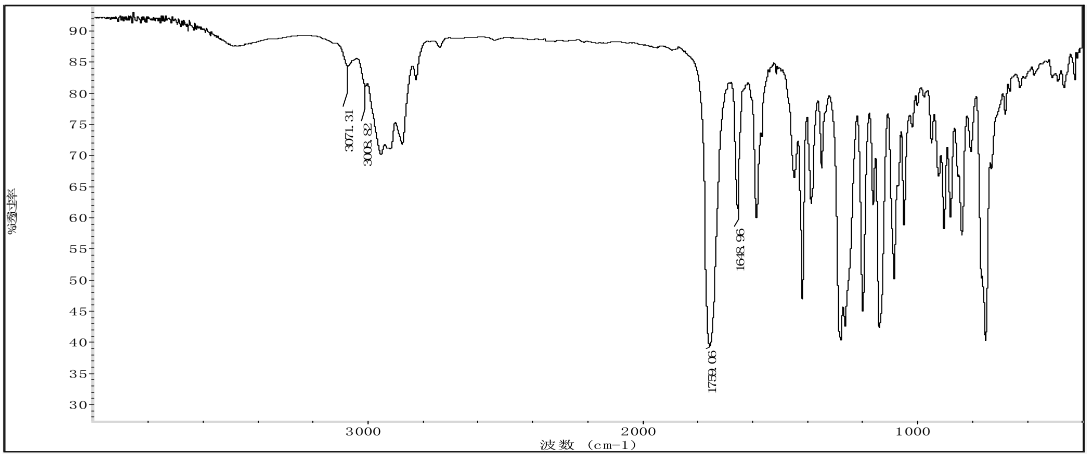

Figure S139. FTIR spectrum of compound (*E*)-**4l′.**

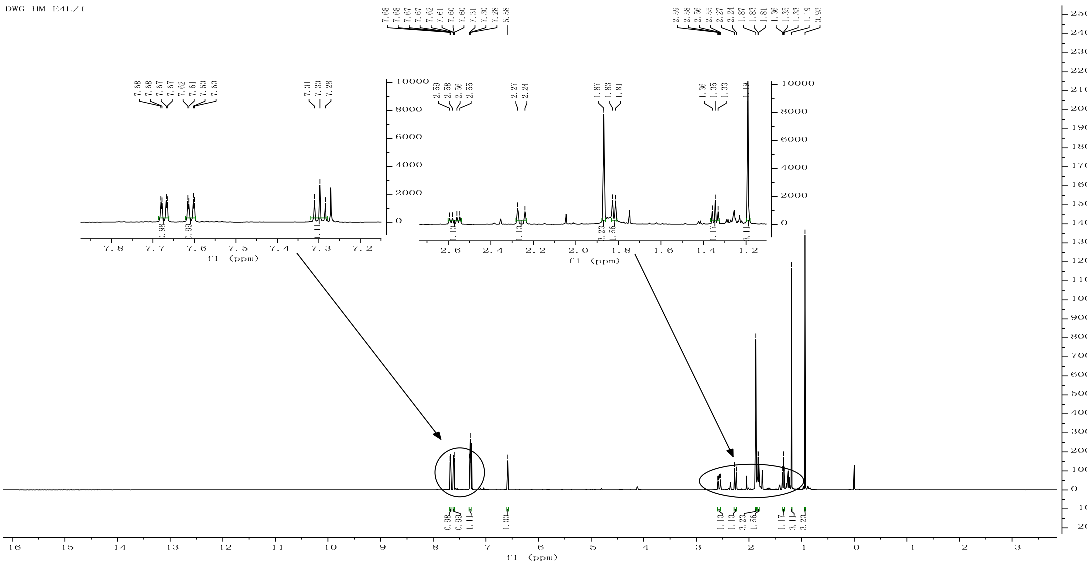

Figure S140. ^{1}H-NMR spectrum of compound (*E*)-**4l′** in $CDCl_3$.

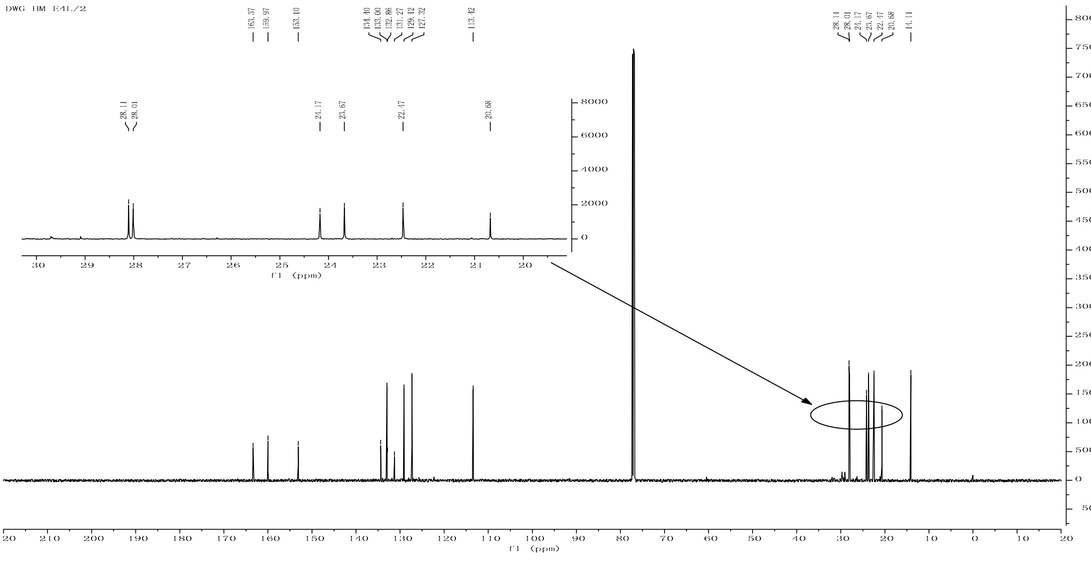

Figure S141. ^{13}C-NMR spectrum of compound (*E*)-**4l′** in $CDCl_3$.

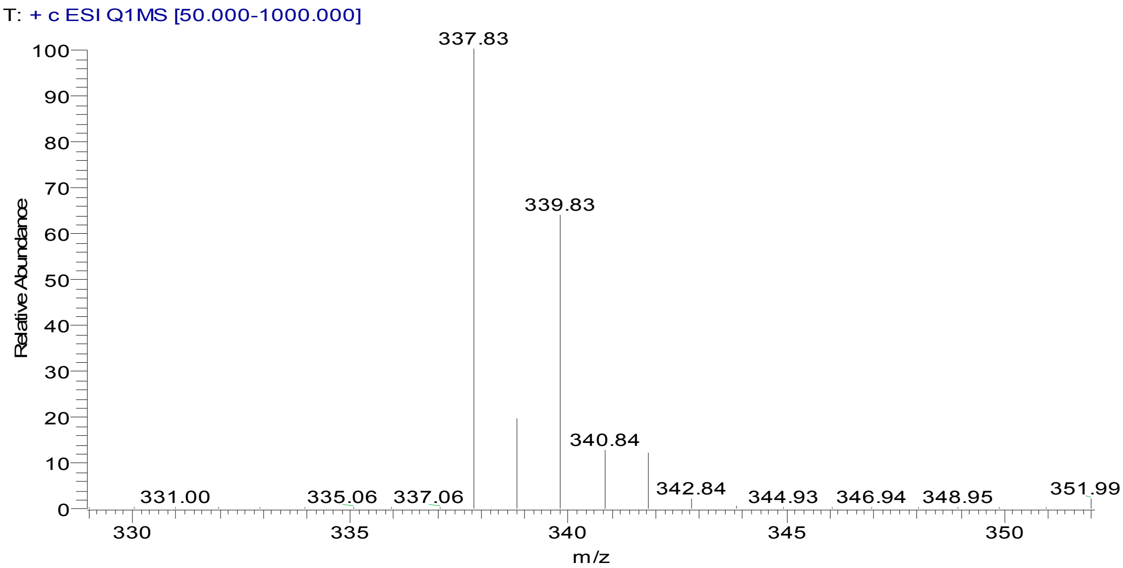

Figure S142. ESI-MS spectrum of compound (*E*)-**4l'**.

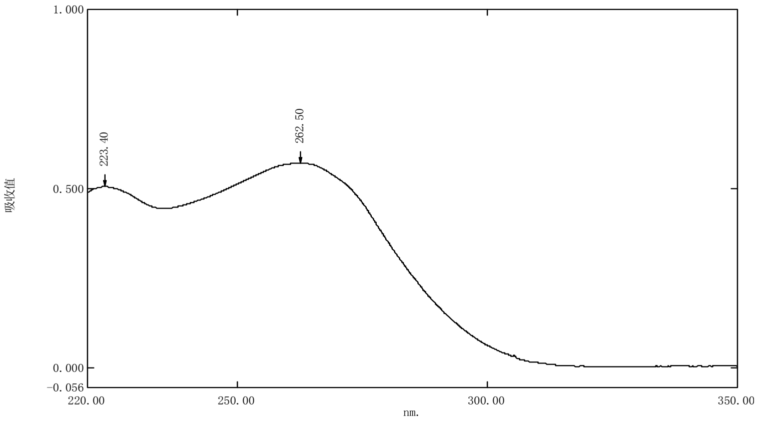

Figure S143. UV-vis spectrum of compound (*E*)-**4r'**.

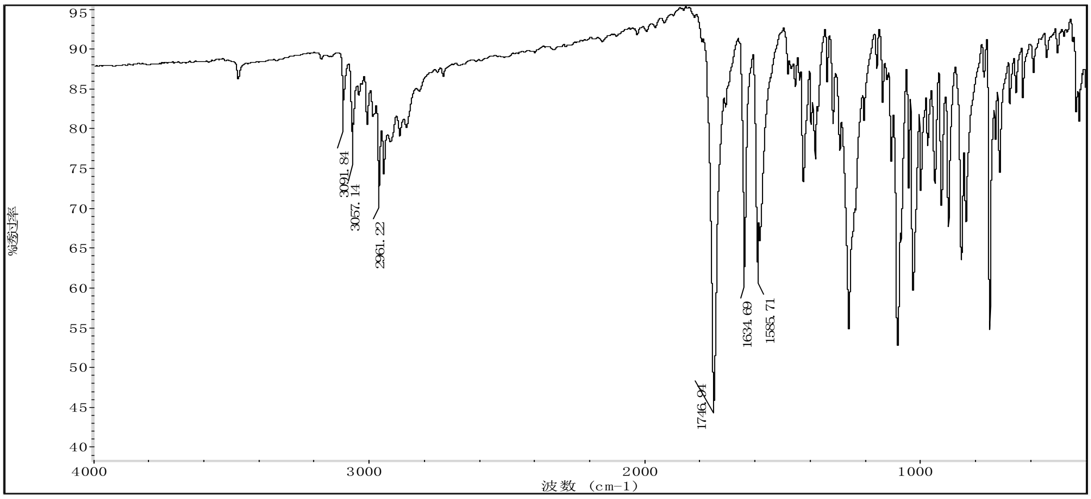

Figure S144. FTIR spectrum of compound (*E*)-**4r'**.

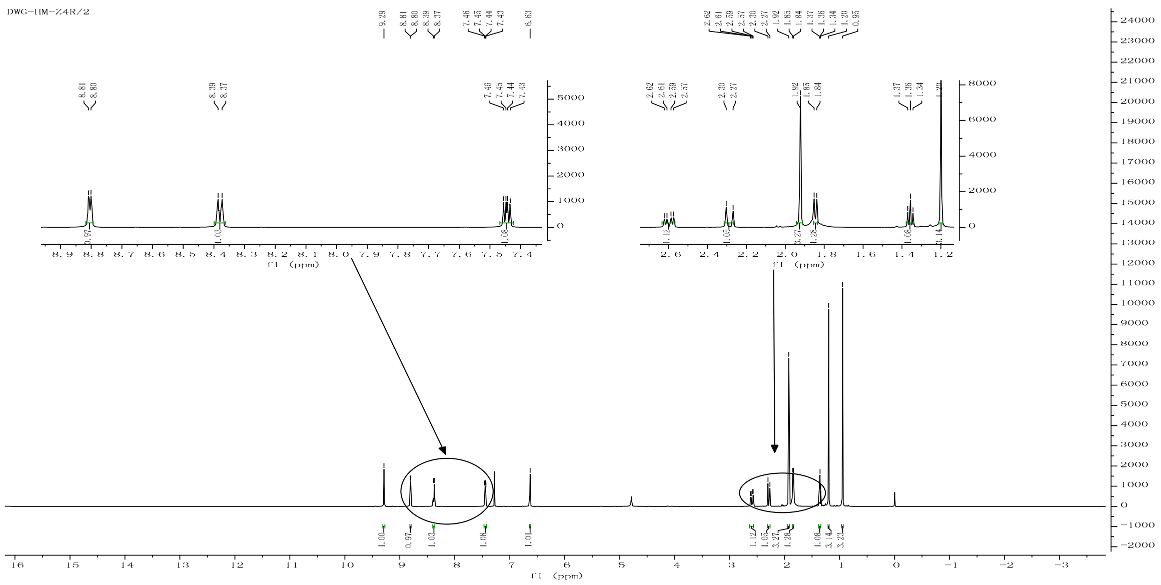

Figure S145. ^{1}H-NMR spectrum of compound (*E*)-**4r′** in $CDCl_3$.

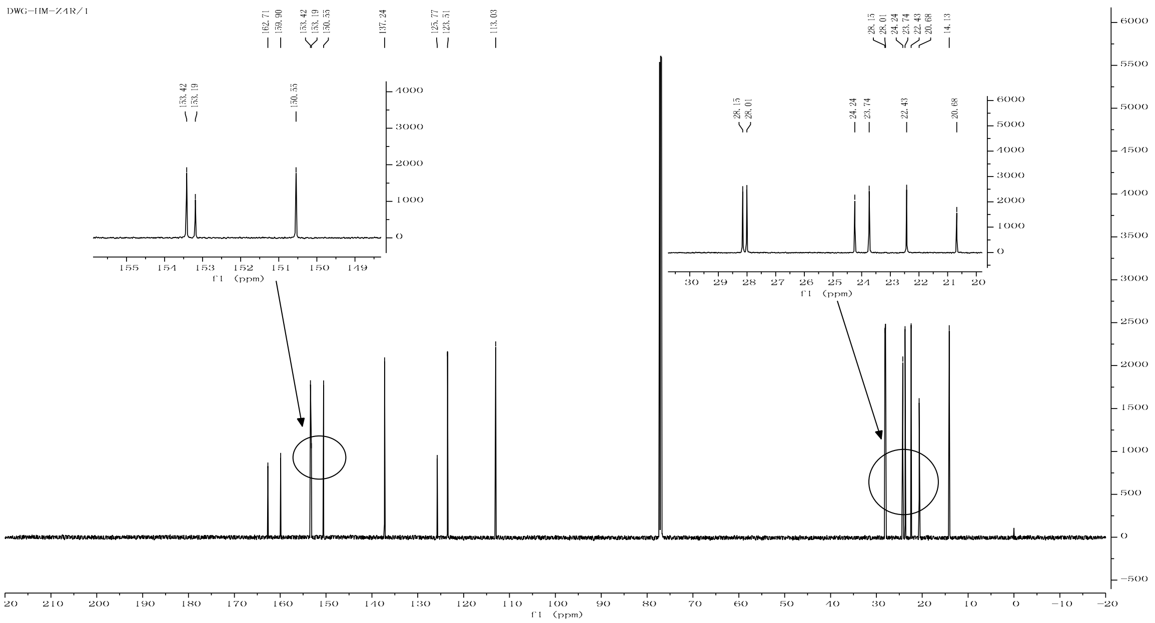

Figure S146. ^{13}C-NMR spectrum of compound (*E*)-**4r′** in $CDCl_3$.

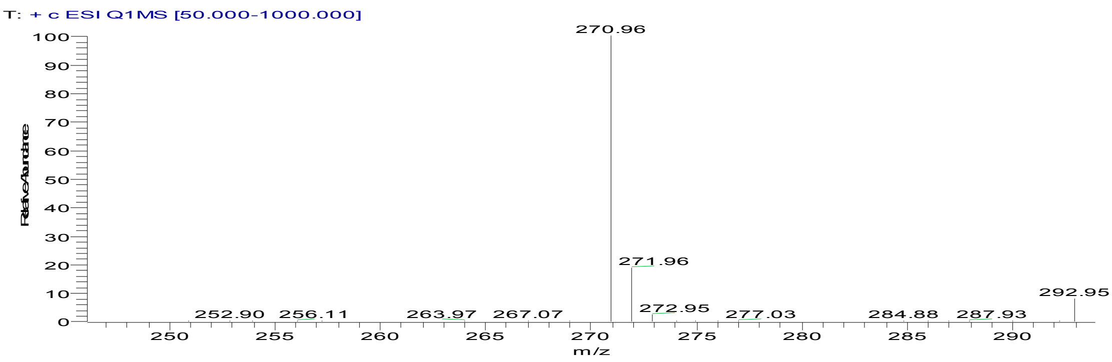

Figure S147. ESI-MS spectrum of compound (*E*)-**4r′**.
